# Presence of sputum IgG against eosinophilic inflammatory proteins in asthma

**DOI:** 10.3389/fimmu.2024.1423764

**Published:** 2024-07-18

**Authors:** Rundong Qin, Fei Long, Pingan Zhang, Renbin Huang, Hao Hu, Yubiao Guo, Zhenyu Zheng, Jing Xiao, Li He, Tao Peng, Jing Li

**Affiliations:** ^1^ State Key Laboratory of Respiratory Disease, National Clinical Research Center for Respiratory Disease, Guangzhou Institute of Respiratory Health; Department of Allergy and Clinical Immunology, The First Affiliated Hospital of Guangzhou Medical University, Guangzhou, Guangdong, China; ^2^ Sino-French Hoffmann Institute, School of Basic Medical Sciences, State Key Laboratory of Respiratory Disease, Guangzhou Medical University, Guangzhou, China

**Keywords:** autoimmune, asthma, autoantibodies, sputum, eosinophil (EOS)

## Abstract

**Background:**

Sputum immunoglobulin G (Sp-IgG) has been discovered to induce cytolytic extracellular trap cell death in eosinophils, suggesting a potential autoimmune mechanism contributing to asthma. This study aimed to explore the potential origin of Sp-IgG and identify clinically relevant subtypes of Sp-IgG that may indicate autoimmune events in asthma.

**Methods:**

This study included 165 asthmatic patients and 38 healthy volunteers. We measured Sp-IgG and its five subtypes against eosinophil inflammatory proteins (Sp-IgG_EPs_), including eosinophil peroxidase, eosinophil major basic protein, eosinophil-derived neurotoxin, eosinophil cationic protein, and Charcot-Leyden Crystal protein in varying asthma severity. Clinical and Mendelian randomization (MR) analyses were conducted. A positive Sp-IgG_EPs_ signature (Sp-IgG_EPs+_) was defined when any of the five Sp-IgG_EPs_ values exceeded the predefined cutoff thresholds, calculated as the mean values of healthy controls plus twice the standard deviation.

**Results:**

The levels of Sp-IgG and Sp-IgG_EPs_ were significantly elevated in moderate/severe asthma than those in mild asthma/healthy groups (all p < 0.05). Sp-IgG levels were positively correlated with airway eosinophil and Sp-IgG_EPs_. MR analysis showed causality between eosinophil and IgG (OR = 1.02, 95%CI = 1.00-1.04, p = 0.020), and elevated IgG was a risk factor for asthma (OR = 2.05, 95%CI = 1.00-4.17, p = 0.049). Subjects with Sp-IgG_EPs+_ exhibited worse disease severity and served as an independent risk factor contributing to severe asthma (adjusted-OR = 5.818, adjusted-95% CI = 2.193-15.431, adjusted-p < 0.001). Receiver operating characteristic curve analysis demonstrated that the combination of Sp-IgG_EPs+_ with non-allergic status, an ACT score < 15, and age ≥ 45 years, effectively predicted severe asthma (AUC = 0.84, sensitivity = 86.20%, specificity = 67.80%).

**Conclusion:**

This study identifies a significant association between airway eosinophilic inflammation, Sp-IgG, and asthma severity. The Sp-IgG_EPs_ panel potentially serves as the specific biomarker reflecting airway autoimmune events in asthma.

## Introduction

Asthma, a heterogeneous respiratory condition, exhibits a spectrum of phenotypes and diverse clinical manifestations (1, 2). Traditionally, the conventional framework for comprehending its pathogenesis involves disrupting the balanced T1/T2 immune response (3, 4). However, recent advances in research have revealed that the dysfunctional autoimmunity localized in the airways may play a significant pathological role in the development of asthma (5–8).

According to clinical guidelines, asthma severity is typically categorized as mild, moderate, or severe based on treatment intensity (9). Effectively managing severe asthma presents a significant challenge for healthcare providers. Individuals dealing with severe asthma often necessitate high doses of corticosteroids and biological interventions to achieve and maintain disease control (10). Patients who have severe asthma frequently manifest a variety of clinical characteristics, such as an absence of allergy history, late-onset asthma, resistance to corticosteroid treatment, and concurrent nasosinusitis (11, 12). The underlying explanation for the occurrence of these clinical features in severe asthma remains unclear.

It is worth noting that a concept of a “polyclonal” autoimmune event occurring within the airways of prednisone-dependent asthma patients has been proposed (8). This phenomenon is associated with heightened eosinophil activity and recurrent pulmonary infections, often accompanied by elevated levels of sputum (Sp) immunoglobulin G (IgG) autoantibodies against eosinophil peroxidase (Sp-IgG_EPX_). It is important to note that the presence of autoantibodies, such as those targeting cytokines, is common in healthy individuals and plays a role in various crucial immune functions. These autoantibodies can exist without leading to autoimmune diseases (13). However, sputum IgG autoantibodies (Sp-IgG) were found to directly induce cytolytic extracellular trap cell death in eosinophils, which contributed to asthma severity. A recent prospective clinical study involving 148 asthmatic patients has further substantiated the presence of airway autoreactivity in individuals with moderate to severe asthma despite ongoing anti-inflammatory treatment (5). These findings underscore the emerging significance of autoimmunity in the pathophysiology of asthma and raise important questions about its role in asthma severity.

IgG autoantibodies play a pivotal role in autoimmunity, and their presence is a hallmark feature (14, 15). In asthma, eosinophil degranulation is a key contributor to the disease’s pathophysiology (16). Eosinophil degranulation entails the release of five main types of inflammatory proteins, including eosinophil peroxidase (EPX), eosinophil major basic protein (MBP), eosinophil-derived neurotoxin (EDN), eosinophil cationic protein (ECP), and Charcot-Leyden Crystal protein (CLC) (17). These proteins contribute significantly to the impairment of respiratory epithelial function and are responsible for histopathological abnormalities seen in asthma patients (18, 19). A comprehensive examination of autoimmune events stemming from eosinophil degranulation in the airways could provide valuable insights into the role of autoimmune dysfunction in asthma.

In the present study, we first explored the relationship between eosinophilic inflammation and Sp-IgG at the clinical level. We then validated this relationship using Mendelian randomization (MR) analysis at the genetic level. MR analysis is a robust epidemiological method that estimates genetic variants linked to risk factors to determine the causal relationship between exposure and outcome (20). This method is effective because it prevents confounding factors and reverse causation bias from influencing the results. Afterward, we identified whether specific types of Sp-IgG against eosinophil-released proteins (Sp-IgG_EPs_), including EPX (Sp-IgG_EPX_), MBP (Sp-IgG_MBP_), EDN (Sp-IgG_EDN_), ECP (Sp-IgG_ECP_), and CLC (Sp-IgG_CLC_), present sufficient clinical relevance. These specific types of Sp-IgG could potentially serve as indicators of autoimmune events in asthma, providing an updated perspective for further studies on dysfunctional autoimmune responses in asthma.

## Methods

### Study design and subjects

We conducted a prospective observational study spanning from December 2017 to December 2020. During this period, we screened adult patients with asthma who were receiving treatment at the Department of Allergy and Clinical Immunology at The First Affiliated Hospital of Guangzhou Medical University. Control volunteers were recruited through local advertising efforts. All participants with asthma met the following inclusion criteria: (1) a confirmed diagnosis of asthma by an expert physician, supported by objective evidence (defined as meeting at least one of the following criteria: peak flow variation ≥ 20% over a 2-week period, bronchodilator reversibility ≥ 12% and > 200 mL, or airway hyperresponsiveness [methacholine PC_20_ ≤ 8 mg/mL]); (2) undergoing regular controller medication treatment for a minimum of six months. Subjects were excluded if they: (1) were undergoing maintenance with biologics or had taken biologics within the last 6 months (considering the potential impact on autoimmune response and the consistent clinical background of subjects); (2) had a physician-diagnosed autoimmune disease or exhibited current or past symptoms suggestive of an undiagnosed autoimmune disease; (3) suffered from other respiratory conditions, including emphysema, chronic obstructive pulmonary disease, bronchiectasis, pneumonia, or lung damage attributed to prior medical conditions such as pulmonary tuberculosis; (4) had a significant disease affecting other vital organs, such as cardiovascular conditions and malignancies. Healthy volunteers had no prior history of chronic disorders, including respiratory or autoimmune diseases.

Recruitment was conducted through a fixed team that included three highly experienced clinicians in the asthma field. Each subject was assessed independently by each clinician, and only when there was unanimous agreement among the three clinicians was the subject finally included. A standardized assessment was conducted for all subjects, encompassing age, body mass index (BMI), asthma duration, pulmonary function, Sp induction, allergy status, fractional exhaled nitric oxide (FeNO) levels, and blood examinations. Asthma treatment intensity was utilized as an indicator of disease severity and classified in accordance with the Global Initiative for Asthma (GINA) treatment steps (9), resulting in the following severity strata: GINA 1–2 (mild), GINA 3 (moderate), and GINA 4–5 (severe). Asthma control condition was assessed by using a validated questionnaire, the Asthma Control Test (ACT). The ACT comprises five questions pertaining to asthma symptoms, medication usage, and the impact of asthma on daily activities over the preceding four weeks (21). Each question is rated on a scale from 1 to 5, with higher scores indicating better asthma control. Asthma control is categorized as follows: well-controlled asthma (total score of 20 to 25), partially controlled asthma (total score of 16 to 19), and uncontrolled asthma (total score of less than 16).

The study received approval from the Ethics Review Board of the First Affiliated Hospital of Guangzhou Medical University (medical ethics year 2017, No. 25), and all participants provided written informed consent. All personal information was anonymized and stored securely, accessible only to authorized research personnel. Data was coded and de-identified before analysis to maintain confidentiality. Participants were informed about their right to withdraw from the study at any time without any consequences.

### Pulmonary function and FeNO

Pulmonary function tests were conducted by trained operators using a spirometer (MasterScreen PFT; Jaeger™, CareFusion, Hoechberg, Germany) in strict accordance with the guidelines established by the American Thoracic Society and the European Respiratory Society (22, 23). Various parameters were assessed, including the percent of predicted forced vital capacity (FVC% predicted), forced expiratory volume in 1 second (FEV_1_% predicted), and midflows (average forced expiratory flow during the mid (25%-75%) portion of the FVC maneuver). Fractional exhaled nitric oxide (FeNO) levels were measured using a portable, rapid-response chemiluminescent analyzer with an expiratory flow rate of 50 mL/s (NIOX System, Aerocrine, Sweden) (24, 25).

### Allergen sensitization

Allergen sensitization was assessed by detecting specific immunoglobulin E (IgE) responses to allergens using the ImmunoCAP^®^ assay (Phadia, Uppsala, Sweden). Subjects were classified as atopic if they exhibited at least one positive response to a common aeroallergen, which included house dust mites, cats, dogs, grass pollen, tree pollen, and a mixture of molds.

### Collection and processing of induced Sp and serum samples

Sp induction followed a previously established method (26). Briefly, patients received two puffs of salbutamol (100 μg/puff) 15 minutes before the induction procedure. Sp was induced in each subject by inhaling a 3% hypertonic saline solution for 15 minutes. To minimize potential oral contamination, patients rinsed their mouths with a 0.9% saline solution before expectorating Sp and blowing their noses. Sp samples were expectorated into a collection cup. The initial portion of Sp was discarded, and the inhalation procedure continued for an additional 15 minutes. Subsequently, eight volumes of phosphate-buffered saline (PBS) were added to the collected Sp, followed by vigorous mixing on a plate shaker for 15 minutes at 4°C. The Sp sample was then centrifuged at 3,000 revolutions per minute for 10 minutes at 4°C. Four volumes of the supernatant from the Sp were collected and stored at -80°C, and two volumes of dithiothreitol solution were added to the Sp for 15 minutes to solubilize mucus. After another round of centrifugation, A portion of the cell pellet is spread onto microscope slides to prepare smears. These smears are then fixed and stained using Hematoxylin and Eosin stains, which highlight different cell types. Under a light microscope, various cell types are identified based on their distinct morphological characteristics. Typically, 200-400 cells are counted to determine the relative percentages of neutrophils, eosinophils, macrophages, and lymphocytes. The percentage of each cell type is calculated by dividing the number of each cell type by the total number of cells counted and then multiplying by 100.

Peripheral whole blood samples were drawn from the enrolled patients, and a portion of these samples was used for peripheral blood cell analysis using an automated hematology analyzer (UniCel DxH 800; Beckman Coulter, Miami, Fla). The remaining samples were centrifuged at 3,000 revolutions per minute for 10 minutes at 4°C to obtain serum (Se) samples, which was then stored at -80°C for further analysis.

### Detection of total IgG levels in Se and Sp samples

Sp-IgG and Se IgG (Se-IgG) levels of our included patients were determined using commercial measurement kits (Thermo Fisher, Catalog BMS2091), following the manufacturer’s instructions.

### Detection of specific IgG autoantibodies

Elevated Sp-IgG levels indicate a generalized B cell response in the airways without specifying the exact antigen. Specific IgG autoantibody testing enables more accurate identification of the specific antigen triggering the autoimmune reaction, facilitating precise diagnosis. Five eosinophil-released proteins, specifically CLC (USBiological, Catolog 153962), EPX (Cloud-Clone Corp, Catolog RPJ138Hu01), MBP (USBiological, Catolog 153962), EDN (USBiological, Catolog 375071), and ECP (USBiological, Catolog 138728), were previously immobilized onto multiplex magnetic beads (Bio-Rad, Hercules, CA, USA). These beads were subsequently incubated with Sp supernatant and Se samples, which were diluted to respective concentrations of 1:10 and 1:180, at 37°C for a duration of 1 hour. After this incubation period, the beads underwent three wash cycles using the Bio-Plex Pro™ wash station (Bio-Rad). To detect the bound antibodies, biotin-conjugated anti-human IgG (ThermoFisher, Waltham, MA, USA) at a 1:1000 dilution was added to each well and incubated at 37°C for 1 hour. Following this step, the wells underwent an additional three wash cycles. Subsequently, streptavidin-R-phycoerythrin (Bio-Rad) at a 1:100 dilution was added to each well and incubated at 37°C for 15 minutes. The beads were once again washed three times and resuspended in assay buffer. The mean fluorescence intensity of each uniquely encoded microsphere was quantified using a Bio-Plex 200 instrument (Bio-Rad). The results were obtained using Bio-Plex Manager™ 6.0 software (Bio-Rad). The assay was validated according to the following steps: (1) Sensitivity: The lower limit of detection (LOD) was determined by measuring the mean fluorescence intensity (MFI) of blank samples plus three standard deviations. The LOD for each autoantibody was consistently below the lowest standard concentration used in the assay; (2) Specificity: Specificity was assessed by testing the mixed coupled beads and individual coupled bead assays with gradient-diluted ChromPure human IgG (Jackson, Catalog 009-000-003). The results of autoantigens detection showed no significant difference between the two assays.; (3) Validation: We conducted parallel testing with known positive and negative control samples to ensure accuracy.

### Clinical relevance analysis

Statistical analysis was performed using the SPSS software package (version 22.0; IBM Corp., Armonk, NY). Continuous variables are presented as numbers (%), median (interquartile range), or mean (standard deviation). Normality testing was employed to determine whether the data adhered to a normal distribution. Comparisons of continuous endpoints between asthmatic subjects and healthy controls were calculated based on the variable normality assumptions using independent-sample t-tests or Mann-Whitney U tests. Similarly, comparisons among asthma subgroups were conducted using ANOVA or Kruskal-Wallis tests. Categorical endpoints were analyzed using a χ2 test. Correlation analyses were conducted to evaluate the relationship between Sp-IgG_EPs_ and clinical parameters utilizing either Pearson’s correlation or Spearman’s correlation, depending on the normality assumptions of the variables. Additionally, Pearson’s partial correlation was applied to assess the relationship between Sp-IgG_EPs_ and clinical parameters while controlling for covariate effects. Univariate and multivariate logistic regression analyses were employed to ascertain the association between Sp-IgG_EPs_ and severe asthma. Except for the Sp-IgG_EPs_, covariates were included in the models based on statistical differences across asthma severity groups: Age (45 years) to distinguish middle-aged adults with significant physiological changes (27), BMI categories, age of asthma onset (18 years) to differentiate childhood vs. adult-onset asthma, sputum eosinophil count (3%) for identifying eosinophilic inflammation (28), and standard ACT score ranges for asthma control assessment (21). Receiver operating characteristic (ROC) curves were constructed to evaluate the ability of Sp-IgG_EPs_ to predict severe asthma. A P value of <0.05 was considered statistically significant.

### MR analysis

To validate our clinical observations, we conducted a two-step, two-sample Mendelian Randomization (MR) analysis using publicly available datasets. This investigation focused on the genome-wide association among eosinophil count, IgG levels, and asthma. We obtained Genome-Wide Association Studies (GWAS) data on eosinophil count (GWAS ID: ieu-b-33), comprising 563946 European individuals, from the GWAS catalog (https://gwas.mrcieu.ac.uk/datasets/ieu-b-33/). Similarly, GWAS data on IgG levels (GWAS ID: ebi-a-GCST006357), involving 1000 European individuals, were sourced from the same catalog (https://gwas.mrcieu.ac.uk/datasets/ebi-a-GCST006357/). Additionally, asthma-related GWAS data were retrieved from the Finnish database, encompassing 230909 European participants (5206 cases and 225703 controls), which can be accessed (https://storage.googleapis.com/finngen-public-data-r10/summary_stats/finngen_R10_ASTHMA_ALLERG.gz). Detailed information is provided in **Supplementary Table 1**.

The primary method used to estimate the causal association between eosinophil count, IgG levels, and asthma was the Inverse Variance Weighted (IVW) approach. This analysis was conducted in two steps. In the first step, we tested the causal effects of eosinophil count (as the exposure) on IgG production (as the outcome). In the second step, we examined the causal effect of IgG levels (as the exposure) on asthma (as the outcome).

Single-nucleotide polymorphisms (SNPs) were defined as instrumental variables (IVs) for this analysis (29). SNPs with significant associations for eosinophil count (p < 5 × 10^-8^) were included. Because few genetic variants were available, and based on previous studies, a relatively relaxed threshold (p < 1 × 10^-5^) was used to select SNPs for IgG levels. SNPs in linkage disequilibrium (LD) were excluded from the analysis, with the LD condition set to r² < 0.001 and a physical distance > 10,000 kb. To address potential weak instrument bias, we calculated the F-statistic for each SNP and excluded those with an F-statistic <10, as these were considered weak IVs that could introduce bias into the results. Cochran’s Q test was used to evaluate the heterogeneity among the SNPs. To assess the potential influence of individual SNPs on the results, we conducted a leave-one-out analysis, which involved sequentially excluding each SNP and performing the IVW method on the remaining SNPs. MR Pleiotropy RESidual Sum and Outlier (MR-PRESSO) and MR-Egger regression were employed to examine potential horizontal pleiotropy effects. MR-PRESSO detected significant outliers and corrected for horizontal pleiotropy by removing these outliers (30).

All MR analyses were conducted using the R packages ‘TwoSampleMR (V0.5.7)’ and ‘MendelianRandomization (V0.9.0)’. Results were reported as odds ratios (ORs) with 95% confidence intervals (CIs). All statistical analyses were performed in R (V.4.3.1). This MR analysis constitutes a secondary analysis of publicly available GWAS summary statistics. Ethical approval was obtained for each of the original GWAS studies, and no individual-level data were used, thereby obviating the need for new ethical review board approval.

## Results

### Patients

Following the initial screening, we successfully enrolled 209 asthmatic patients and 60 healthy volunteers. However, 44 asthmatic patients and 22 healthy controls were subsequently excluded due to the failure of Sp induction. Consequently, our final analysis included 165 asthmatic patients and 38 healthy volunteers. No significant differences in demographic features, including age, gender, and BMI, were observed between these two groups.

In accordance with the guidelines established by GINA, the 165 asthma patients were classified into three distinct categories: 68 with mild asthma, 37 with moderate asthma, and 60 with severe asthma. Among the severity subgroups, subjects with severe asthma exhibited the worst clinical features. These included the highest levels of sputum eosinophil count and daily dosage of inhaled corticosteroids, the highest frequency of acute exacerbations per year, the highest proportion of maintenance oral corticosteroid and long-acting muscarinic antagonist use, and the lowest lung function parameters. Detailed information comparing asthmatic subjects with healthy controls, as well as different severity groups, is presented in **Table 1**.

**Table 1:** The demographic and clinical characteristics of included subjects.

Overall				Patient subgroups			
Subjects’ characteristics	Healthy controls (N=38)	Asthmatic patients (N=165)	p-value	Mild asthma (N=68)	Moderate asthma (N=37)	Severe asthma (N=60)	p-value
Age (years)^&^	37.50 (21.75;35.00)	38.00 (16.89;25.67)	0.784	34.00 (25.00;43.00)	34.00 (31.50;45.00)	44.00 (36.00;51.00)	<0.001
Gender (male, %)^#^	23.00 (60.52)	105.00 (63.64)	0.612	43 (63.24)	25.00 (67.50)	37 (61.60)	0.838
Body mass index^&^	21.00 (18.23;23.00)	21.24 (16.89;25.67)	0.541	22.83 (17.99;27.51)	20.50 (18.13;24.32)	20.10 (14.92;25.60)	0.184
Age of asthma onset (years)^&^	–	30.00 (16.50;40.00)	–	27.50 (14.00;40.50)	29.50 (17.00;37.75)	32.00 (23.00;38.50)	0.120
Number of exacerbations in past year (%)							
0^#^	–	148.00 (89.70)	–	67.00 (98.53)	36.00 (97.30)	45.00 (75.00)	<0.001
1^#^	–	12.00 (72.73)		1.00 (1.47)	1.00 (2.70)	10.00 (16.67)	
≥2^#^	–	5.00 (3.03)		0.00 (0.00)	0.00 (0.00)	5.00 (8.33)	
Allergen sensitizations (%)^#^	0.00 (0.00)	74.00 (44.85)	<0.001	37.00 (54.40)	15.00 (40.50)	22.00 (36.60)	0.110
Serum total IgE (IU/mL)^&^	–	229.00 (109.25;391.50)	–	280.50 (102.60;557.50)	213.00 (104.00;400.00)	229.00 (126.00;334.25)	0.588
Dose of maintenance ICS (BDP; μg/d)^&^	–	400.00 (200.00; 800.00)	–	200.00 (200.00;200.00)	400.00 (400.00;400.00)	800.00 (800.00;800.00)	<0.001
Asthma control test score^&^	–	20.00 (16.00;24.00)	–	22.00 (19.00;24.00)	22.00 (17.00;24.00)	17.00 (17.00;21.00)	<0.001
FeNO (ppb)^&^	19.00 (15.00;23.00)	51.20 (28.00;87.50)	<0.001	55.50 (33.00;96.25)	47.50 (28.00;75.00)	49.00 (25.00;85.00)	0.213
Parameters of lung function							
FEV_1_ (% predicted)^&^	98.50 (91.35;103.91)	74.55 (54.48;88.75)	<0.001	88.00 (79.87;97.36)	76.00 (69.00;87.00)	49.10 (37.63;59.70)	<0.001
FEF_25-75_ (% predicted)^&^	92.95 (78.68;102.54)	36.20 (20.10;53.60)	<0.001	54.75 (40.67;69.70)	37.27 (31.80;43.50)	15.13 (10.60;23.00)	<0.001
FVC (% predicted)^&^	96.12 (90.37;101.43)	92.49 (81.33,103.74)	<0.001	98.89 (89.51;110.74)	98.80 (88.25;105.20)	78.25 (68.20;88.20)	<0.001
FEV_1_/FVC^&^	92.73 (85.40;96.73)	71.03 (60.23;78.99)	<0.001	78.94 (73.74;87.16)	71.15 (65.08;77.79)	56.50 (46.66;64.35)	<0.001
Peripheral eosinophil count (10^9^/L)^&^	0.10 (0.05;011)	0.32 (0.16;0.50)	<0.001	0.32 (0.20;0.43)	0.30 (0.11;0.41)	0.39 (0.14;0.62)	0.420
Induced sputum							
Neutrophil (%)^&^	35.31 (20.28;48.47)	44.00 (24.04;63.87)	<0.001	48.00 (26.76;62.07)	33.60 (14.47;52.53)	43.80 (23.59;73.40)	0.722
Eosinophil (%)^&^	0.00 (0.00;0.68)	17.03 (4.43;48.93)	<0.001	9.60 (2.80;28.00)	27.84 (11.00;62.00)	27.00 (4.61;67.09)	0.004
Lymphocyte (%)^&^	1.50 (0.79;3.43)	0.50 (0.00;1.55)	<0.001	1.34 (0.03;2.33)	0.20 (0.00;0.63)	0.40 (0.00;1.00)	0.034
Macrophage (%)^&^	61.77 (50.93;75.58)	18.33 (6.22;41.83)	<0.001	31.20 (14.40;44.00)	22.35 (10.41;43.23)	8.00 (3.69;23.60)	<0.001
Combination of medication							
LABA (%)^#^	–	161.00 (97.58)	–	64.00 (94.12)	37.00 (100.00)	60.00 (100.00)	0.054
Oral CS (%)^#^	–	10.00 (6.06)	–	0.00 (0.00)	0.00 (0.00)	10.00 (16.67)	<0.001
LTRA (%)^#^	–	15.00 (9.09)	–	0.00 (0.00)	0.00 (0.00)	15.00 (25.00)	<0.001
LAMA (%)^#^	–	20.00 (12.12)	–	0.00 (0.00)	0.00 (0.00)	20.00 (33.33)	<0.001

The symbols “&” and “#” indicate data representation as ± median (interquartile range) and percentage (%), respectively. Comparisons of continuous endpoints between asthmatic subjects and healthy controls were calculated using Mann-Whitney U tests. Similarly, comparisons among asthma subgroups were conducted using Kruskal-Wallis tests. Categorical endpoints were analyzed using a χ2 test. FEV_1_, forced expiratory flow in 1 second; FVC, forced vital capacity; FEF_25-75_, forced expiratory flow between 25 and 75% of vital capacity; IgE, immunoglobulin E; FeNO, Fractional exhaled nitric oxide; CS, corticosteroid; ICS, inhaled corticosteroid; BDP, Beclomethasone; LABA, Long-Acting Beta2-Agonist; LTRA, Leukotriene Receptor Antagonist; LAMA, Long-Acting Muscarinic Antagonist.

The meaning of the symbol “-” is "not applicable."

### Sp-IgG correlated with airway eosinophilic inflammation and asthma severity

**Figure 1:**
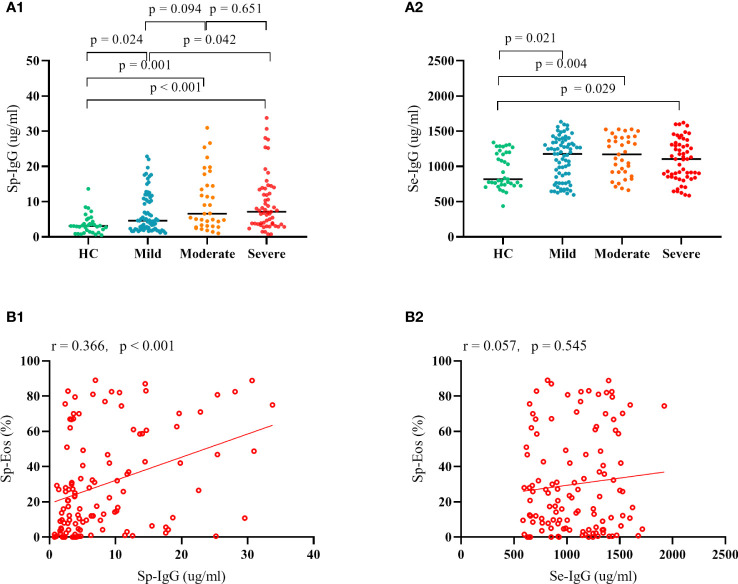
The clinical relevance of IgG in asthma. Plot **(A1)** and Plot **(A2)** depict the comparison in Sp-IgG and Se-IgG between healthy controls and patients with different severity of asthma; Plot **(B1)** and Plot **(B2)** illustrate the correlation between Sp-IgG and Sp-Eos, as well as Se-IgG and Sp-Eos, in asthmatic subjects. Sp, sputum; Se, serum; IgG, immunoglobulin G; Eos, eosinophil.

We observed a significant elevation in the levels of Sp-IgG and Se-IgG in patients with asthma, irrespective of its severity (mild, moderate, or severe), compared to the healthy control group (Sp-IgG: p = 0.024, p = 0.001, and p < 0.001, and Se-IgG: p = 0.021, p = 0.004, and p = 0.029, respectively). Furthermore, subjects with severe asthma exhibited significantly higher levels of Sp-IgG compared to those with mild asthma (p = 0.042). However, we did not observe any variation in Se-IgG levels among patients with different severity levels. To investigate the potential source of elevated Sp-IgG, we conducted a correlation analysis between Sp-IgG and airway inflammation cells, including eosinophils, neutrophils, lymphocytes, and macrophages. Our findings revealed a positive correlation between Sp-IgG and the presence of eosinophils in the airways (r = 0.366, p < 0.001), suggesting that eosinophil infiltration is the primary contributor to the increased levels of Sp-IgG in the airways (**Figure 1**). The correlations between Sp-IgG and neutrophils, lymphocytes, and macrophages are depicted in **Supplementary Figure 1**.

### MR analysis reveals causal link between eosinophils, IgG, and asthma

**Figure 2:**
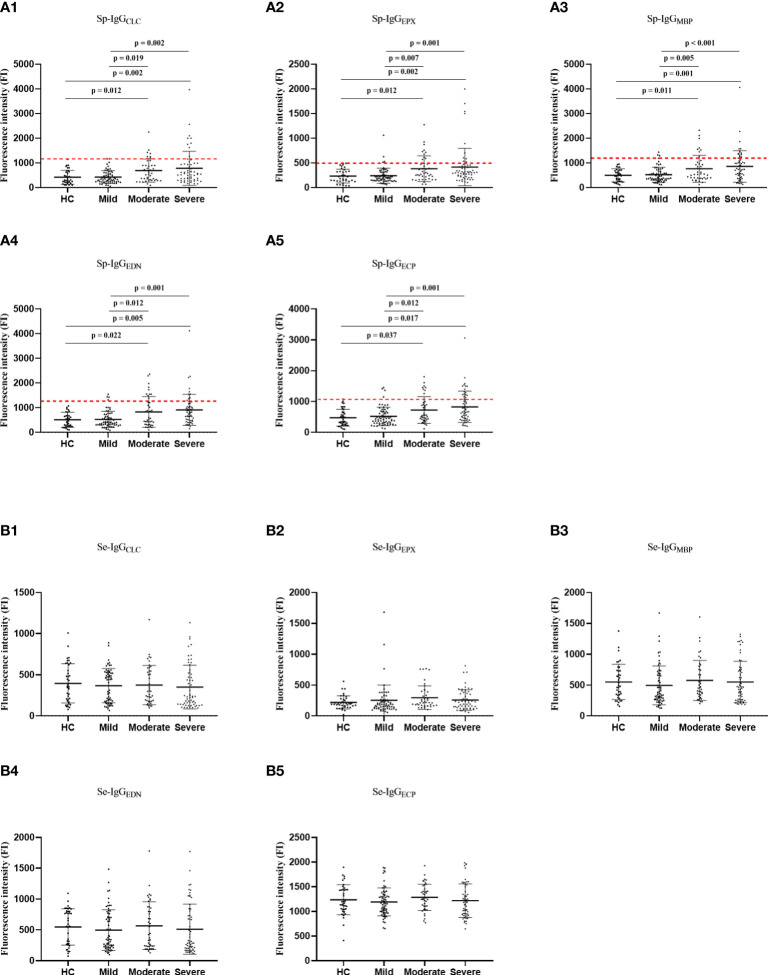
The comparisons in Sp-IgG_EPs_ **(A)** and Se-IgG_EPs_ **(B)** between healthy controls and patients with different severity of asthma. HC, healthy control; Sp, sputum; Se, serum; IgG_EPX_, IgG autoantibodies against eosinophil peroxidase; IgG_MBP_, IgG autoantibodies against major basic protein; IgG_EDN_, IgG autoantibodies against eosinophil neurotoxin; IgG_ECP_, IgG autoantibodies against eosinophil cationic protein; IgG_CLC_, IgG autoantibodies against Charcot-Leyden Crystal protein.

**Figure 3:**
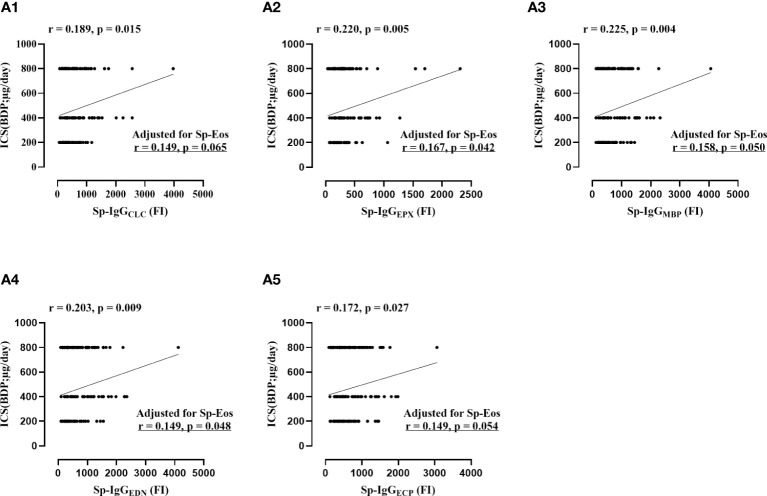
The correlations between the dosage of daily inhaled corticosteroid and sputum autoantibodies against eosinophil released proteins, including Sp-IgG_CLC_ (Plot **A1**), Sp-IgG_EPX_ (Plot **A2**), Sp-IgG_MBP_ (Plot **A3**), Sp-IgG_EDN_ (Plot **A4**), and Sp-IgG_ECP_ (Plot **A5**). Sp, sputum; IgG_EPX_: IgG autoantibodies against eosinophil peroxidase; IgG_MBP_, IgG autoantibodies against major basic protein; IgG_EDN_, IgG autoantibodies against eosinophil neurotoxin; IgG_ECP_, IgG autoantibodies against eosinophil cationic protein; IgG_CLC_, IgG autoantibodies against Charcot-Leyden Crystal protein; ICS, inhaled corticosteroid; BDP, Beclomethasone Dipropionat;. Sp-Eos, sputum eosinophil count.

**Figure 4:**
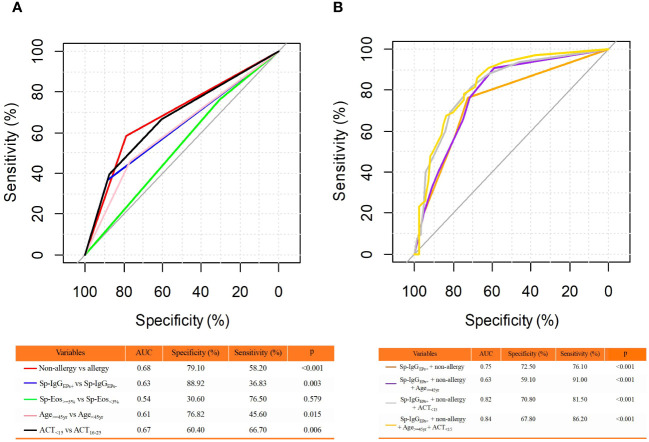
Receiver Operating Characteristic (ROC) Curve Analysis for Predicting Severe Asthma. **(A)** ROC Analysis of Individual Clinical Features; **(B)** ROC Analysis of Combinations of Clinical Features. The clinical features included in the analysis were binary classification variables. AUC, area under the curve; Sp-IgG_EPs+_, a positive test of sputum autoantibodies against eosinophil released proteins; Sp-Eos, sputum eosinophil count; yr, year; ACT, asthma control test.

Leveraging publicly available Genome-Wide datasets, we pinpointed 371 independent SNPs associated with eosinophils as exposure for IgG and 13 independent SNPs associated with IgG as exposure for asthma. The F-statistics for all instrumental variables (IVs) exceeded 10, indicating effective mitigation of weak instrument bias. Initially, a significant correlation emerged between eosinophil levels and genetic susceptibilities linked to elevated IgG production (OR = 1.02, 95% CI = 1.00-1.04, p = 0.020). This finding reinforces the speculation that airway IgG results from airway eosinophilic inflammation. Furthermore, elevated IgG levels were significantly correlated with an increased risk of asthma (OR = 2.05, 95% CI = 1.00-4.17, p = 0.049). Cochran’s Q test and pleiotropy test did not show evidence of heterogeneity and horizontal pleiotropy in any of the reported results (all p > 0.05). The results of the two-step, two-sample MR analysis are presented in **Supplementary Table 2**. The detailed results of the characteristics of SNPs, MR-PRESSO analysis, Single SNP analysis, Leave-one-out analysis, and the scatter plot of SNPs effect are presented in the **Supplementary Tables 3**–**14** and **Figures 2**–**4**.

### The association between Sp-IgG_EPs_ and the severity of asthma

Regarding specific Sp-IgG, the levels of Sp-IgG were positively correlated with the five types of Sp-IgG_EPs_, including Sp-IgG_CLC_ (p = 0.246 and p = 0.005), Sp-IgG_EPX_ (p = 0.339 and p < 0.001), Sp-IgG_EDN_ (p = 0.264 and p < 0.001), Sp-IgG_ECP_ (p = 0.349 and p < 0.001) and Sp-IgG_MBP_ (p = 0.319 and p < 0.001) (**Supplementary Figure 5**). We observed a noteworthy increase in the levels of all five types of Sp-IgG_EPs_ among individuals with moderate and severe asthma groups compared to those with mild asthma (Sp-IgG_CLC_: p = 0.019 and p = 0.002; Sp-IgG_EPX_: p = 0.007 and p = 0.001; Sp-IgG_MBP_: p = 0.005 and p < 0.001; Sp-IgG_EDN_: p = 0.012 and p = 0.001; and Sp-IgG_ECP_: p = 0.012 and p = 0.001, respectively), as well as the healthy controls (Sp-IgG_CLC_: p = 0.012 and p = 0.002; Sp-IgG_EPX_: p = 0.012 and p = 0.002; Sp-IgG_MBP_: p = 0.011 and p = 0.001; Sp-IgG_EDN_: p = 0.022 and p = 0.005; and Sp-IgG_ECP_: p = 0.037 and p = 0.017, respectively). However, no statistically significant differences were observed in the levels of the five types of serum autoantibodies to eosinophil-released proteins (Se-IgG_EPs_) among the various groups (**Figure 2**).

Furthermore, we identified positive correlations between the concentrations of Sp-IgG_EPs_ and the daily dosage of inhaled corticosteroid (ICS) (Sp-IgG_CLC_: r = 0.189, p = 0.015; Sp-IgG_EPX_: r = 0.220, p = 0.005; Sp-IgG_MBP_: r = 0.225, p = 0.004; Sp-IgG_EDN_: r = 0.203, p = 0.009; and Sp-IgG_ECP_: r = 0.172, p = 0.027). Additionally, significant correlations were found between the concentrations of Sp-IgG_EPX_, Sp-IgG_EDN_, and Sp-IgG_ECP_ and the levels of eosinophils in the airways (Sp-IgG_EPX_: r = 0.187, p = 0.019; Sp-IgG_EDN_: r = 0.158, p = 0.049; and Sp-IgG_ECP_: r = 0.169, p = 0.034, respectively). After adjusting for sputum eosinophil count, the concentrations of Sp-IgG_EPX_, Sp-IgG_EDN_, and Sp-IgG_MBP_ remained significantly correlated with the daily dosage of inhaled corticosteroids (**Figure 3**).

### Robust inter-correlation among Sp-IgG_EPs_

Strong correlations were evident among the five types of Sp-IgG_EPs_, with correlation coefficients ranging from 0.75 to 0.98. In contrast, correlations among Se-IgG_EPs_ were less pronounced, with coefficients ranging from 0.64 to 0.96. Notably, the correlations between Sp-IgG_EPs_ and Se-IgG_EPs_ were relatively weak, with correlation coefficients spanning from 0.27 to 0.56 (**Supplementary Figure 6**).

### The clinical significance of the Sp-IgG_EPs_ panel in asthma

In light of the robust correlation among Sp-IgG_EPs_, measuring the panel of Sp-IgG_EPs_ might provide a more comprehensive assessment of autoimmune events caused by eosinophil inflammation, particularly when obtaining high-quality Sp samples, is challenging. In this study, individuals demonstrating a positive Sp-IgG_EPs_ panel (Sp-IgG_EPs+_) were identified as those with values exceeding predefined cutoff thresholds for any of the five types of Sp-IgG_EPs_. These thresholds were established by calculating two times the standard deviation above the mean values of healthy controls, as illustrated by the red dashed lines in **Figure 2**. Out of the 165 subjects included in our study, 36 (21.81%) exhibited Sp-IgG_EPs+_, while 129 (78.19%) did not. Subjects with Sp-IgG_EPs+_ presented with a higher proportion of females, an increased frequency of exacerbations in the past year, a higher dosage of maintenance ICS, a greater percentage of long-acting muscarinic antagonist usage, more pronounced impairment in small airways, and a higher proportion of moderate-severe asthma (**Table 2**).

**Table 2:** The comparison between positive and negative Sp-IgG_EPs_ groups.

Subjects’ characteristics	Sp-IgG_EPs+_ (N=36)	Sp-IgG_EPs-_ (N=129)	p-value
Age (years)^&^	44.00 (29.50;50.75)	37.00 (29.00;45.00)	0.238
Gender (female, %)^#^	25.00 (69.44)	90.00 (54.88)	0.045
Body mass index^&^	19.09 (14.91;24.83)	21.54 (17.65;25.91)	0.072
Age of asthma onset (years)^&^	30.00 (13.75;44.25)	29.00 (20.00;38.50)	0.811
Number of exacerbations in past year (%)			
0^#^	25.00 (69.44)	121.00 (93.80)	<0.001
1^#^	6.00 (16.67)	6.00 (4.65)	
≥2^#^	3.00 (8.33)	2.00 (1.55)	
Allergen sensitizations (%)^#^	20.00 (55.56)	112.00 (68.29)	0.144
Serum total IgE (IU/mL)^&^	166.00 (97.05;322.50)	213.00 (297.00)	0.210
Dose of maintenance ICS (BDP; μg/d)^&^	400.00 (400.00;800.00)	400.00 (200.00;800.00)	0.005
Asthma severity			
Mild^#^	5.00 (13.89)	63.00 (48.83)	<0.001
Moderate^#^	14.00 (38.89)	23.00 (17.83)	
Severe^#^	17.00 (47.22)	43.00 (33.33)	
Asthma control test score^#^	20.00 (17.00;23.00)	20.50 (16.00;24.00)	0.447
FeNO (ppb)^#^	49.50 (29.25;86.50)	53.00 (27.50;90.00)	0.843
Parameters of lung function			
FEV_1_ (% predicted)^&^	71.35 (47.05;83.36)	75.60 (57.00;90.00)	0.077
FEF_25-75_ (% predicted)^&^	29.65 (14.08;39.65)	37.55 (22.30;54.85)	0.016
FVC (% predicted)^&^	91.93 (76.80;108.75)	94.21 (81.85;103.61)	0.575
FEV_1_/FVC^&^	67.36 (56.89;79.40)	72.00 (62.10;78.96)	0.175
Peripheral eosinophil count (10^9^/L)^&^	0.32 (0.20;0.54)	0.32 (0.14;0.50)	0.958
Induced sputum			
Neutrophil (%)^&^	50.54 (30.72;65.38)	43.40 (22.58;62.05)	0.125
Eosinophil (%)^&^	15.91 (6.58;61.50)	14.80 (4.05;42.00)	0.223
Lymphocyte (%)^&^	0.48 (0.00;1.00)	0.55 (0.00;1.89)	0.270
Macrophage (%)^&^	10.50 (5.00;22.88)	22.37 (7.57;42.49)	0.036
Combination of medication			
LABA (%)^#^	36.00 (100.00)	125.00 (96.90)	0.577
Oral CS (%)^#^	3.00 (8.33)	7.00 (5.43)	0.456
LTRA (%)^#^	5.00 (13.89)	10.00 (7.75)	0.323
LAMA (%)^#^	10.00 (27.78)	10.00 (7.75)	0.003

Individuals demonstrating a positive panel of specific IgG autoantibodies against eosinophil released proteins (Sp-IgG_EPs+_) were identified as those with values exceeding predefined cutoff thresholds for any of the five types of Sp-IgG_EPs,_ including Sp-IgG_EPX_, Sp-IgG_MBP_, Sp-IgG_EDN_, Sp-IgG_ECP_, and Sp-IgG_CLC_. These thresholds were established by calculating two times the standard deviation above the mean values of healthy controls. The symbols “*”, “&”, and “#” indicate data representation as mean ± standard deviation, median (interquartile range), and percentage (%), respectively. ± .Comparisons of continuous endpoints between asthmatic subjects and healthy controls were calculated based on the variable normality assumptions using independent-sample t-tests or Mann-Whitney U tests. FEV_1_, forced expiratory flow in 1 second; FVC, forced vital capacity; FEF_25-75_, forced expiratory flow between 25 and 75% of vital capacity; IgE, immunoglobulin E; FeNO, Fractional exhaled nitric oxide; ICS, inhaled corticosteroid; BDP, Beclomethasone; LABA, Long-Acting Beta2-Agonist; LTRA, Leukotriene Receptor Antagonist; LAMA, Long-Acting Muscarinic Antagonist.

Utilizing a univariate logistic model, we determined that Sp-IgG_EPs+_ constituted a risk factor for severe asthma (OR = 4.598, 95% CI = 2.071-10.211, p < 0.001). Following the adjustment for covariates, including age, allergy status, and disease control condition, SpAb-EPs_+_ retained its status as a risk factor for severe asthma (adjusted- OR = 5.818, adjusted- 95% CI = 2.193-15.431, adjusted- p < 0.001) (**Table 3**).

**Table 3:** Univariate and multivariate logistic models for Sp-IgG_EPs+_ to predict severe asthma.

	Univariate logistic model			Multivariate logistic model		
Variables	OR	95(CI%)	p	Adjusted-OR	95(CI%)	Adjusted-p
Age >= 45 (years)						
No	Reference			Reference		
Yes	2.780	1.416-5.458	**0.003**	2.299	1.018-5.192	**0.045**
BMI						
>23.5	Reference					
18-23.5	0.802	0.369-1.745	0.579			
<18	2.148	0.985-4684	0.055			
Asthma onset (years)						
< 18	Reference					
>= 18	1.128	0.502-2.535	0.770			
Allergy						
No	Reference			Reference		
Yes	0.189	0.094-0.382	**<0.001**	0.239	0.107-0.536	**0.001**
Sputum eosinophil (%)						
<3	Reference					
>=3	1.434	0.708-2.905	0.317			
Asthma control test score						
<=15	Reference			Reference		
16-20	0.175	0.075-0.406	**0.014**	0.161	0.059-0.435	**0.014**
21-25	0.320	0.129-0.794	**<0.001**	0.268	0.093-0.767	**<0.001**
Sp-IgG_EPs+_						
No	Reference			Reference		
Yes	4.598	2.071-10.211	**<0.001**	5.818	2.193-15.431	**<0.001**

Furthermore, we conducted a ROC analysis to assess the predictive capacity of Sp-IgG_EPs+_ for severe asthma. In predicting severe asthma, the Area Under the Curve (AUC) for Sp-IgG_EPs+_ was 0.63 (specificity = 88.82%, sensitivity = 36.83%). However, the combination of Sp-IgG_EPs+_ with the absence of allergies resulted in a notable increase in AUC to 0.75 (specificity = 72.50%, sensitivity = 76.10%). Moreover, the combination of Sp-IgG_EPs+_ with the absence of allergies, age ≥ 45 years, or an ACT score < 15 each exhibited higher AUC values. Ultimately, the combination of a Sp-IgG_EPs+_, no allergies, age ≥ 45 years, and an ACT score < 15 yielded the highest AUC value of 0.84 (specificity = 67.80%, sensitivity = 86.20%) (**Figure 4**).

## Discussion

To the best of our knowledge, our study first time presents significant associations between airway eosinophilic inflammation, Sp-IgG, and asthma severity by employing both clinical and MR analysis, highlighting the important role of eosinophil-medicated IgG in asthma pathogenesis. Additionally, our research also, for the first time, identified crucial clinical relevance of a panel of Sp-IgG_EPs_ in asthma, presenting a high potentiality to serve as a specific biomarker reflecting airway autoimmune events in asthma.

IgG, a crucial antibody type generated by the B cell immune system in response to foreign invaders or self-antigens, plays a pivotal role in immune responses (31). Despite the role of Sp-IgG in targeting self-antigen clearance (e.g., eosinophilic inflammatory proteins or mediators) and potentially serving as indicators of airway inflammation, *in vivo* experiments have shown that increased IgG autoantibodies can trigger EETosis, contributing to heightened airway inflammation. It is reasonable to consider that the excess production of Sp-IgG, related to dysfunctional B cell responses (8), plays a pathogenic role in asthma. This finding was further validated using MR analysis. MR analysis revealed a causal effect of eosinophils on IgG production, with elevated IgG levels identified as a risk factor for asthma. Unlike traditional observational studies, MR leverages genetic variation as a proxy for exposure, mitigating issues like confounding and reverse causation. By mimicking a randomized controlled trial, MR provides robust evidence for causal inference in epidemiological research. Its advantages include overcoming confounding biases inherent in observational studies, providing insights into potential therapeutic targets, and informing public health interventions based on causal relationships identified through genetic instruments (20). Additionally, MR analyses are less prone to measurement error and recall bias, enhancing the reliability of causal inference compared to traditional observational methods (30). Therefore, combined with the findings from clinical observation and MR analysis, we suggest a potential causal link between eosinophilic inflammation, IgG production, and the development of asthma. Our findings offer updated evidence supporting a concealed autoimmune event marked by an intensified B cell immune response within the airways, predominantly instigated by excessive eosinophil infiltration.

IgG autoantibodies, a distinct subset of IgG, possess the unique ability to recognize and bind to self-antigens (32). This subset of antibodies has been found to be associated with triggering eosinophil degranulation (33), a process contributing to the pathogenesis of asthma. However, the specific type of Sp-IgG that effectively represents autoimmune processes in asthma remains the subject of ongoing investigation.

Over the past decades, a variety of autoantibodies has been detected in the Se samples of individuals with asthma (34–41). However, the clinical relevance of most of these Se autoantibodies within the context of asthma has remained limited. Our previous research has uncovered that Sp autoantibodies primarily respond to localized airway inflammation (42), while Se autoantibodies levels can be influenced by various factors, including age (43, 44). In our current study, we observed no significant variations in Se-IgG_EPs_ across different asthma groups. Conversely, individuals with moderate to severe asthma displayed significantly higher levels of Sp-IgG_EPs_ compared to those with mild asthma. Notably, these elevated Sp-IgG_EPs_ levels were positively correlated with the daily dosage of ICS, independently of sputum eosinophil counts. Previous research has linked elevated Sp-IgG_EPs_ with steroid resistance, increased airway eosinophil degradation, and a higher frequency of exacerbations (5, 8). Our current study provided evidence that these five distinct Sp-IgG_EPs_, including Sp-IgG_EPX_, Sp-IgG_EDN_, Sp-IgG_ECP_, and Sp-IgG_CLC_, all demonstrated similar clinical significance and exhibited robust inter-correlations among them. As a result, the measurement of this panel of Sp-IgG_EPs_ could offer a comprehensive assessment of airway auto-reactivity associated with eosinophil degranulation. This approach has the potential to improve sensitivity and decrease the likelihood of false-negative diagnoses of airway auto-reactivity in asthma, especially when acquiring high-quality sputum samples is challenging due to the intricate procedure of sputum induction and sample processing.

Asthma is a complex and heterogeneous disease with a variety of underlying mechanisms (45, 46). In our study of 165 subjects, only 36 individuals (21.81%) presented Sp-IgG_EPs+_, indicating that the presence of dysfunctional airway autoimmunity is not common in asthma, which is not easy to capture in clinical practice. Compared to the subjects without Sp-IgG_EPs+_, those individuals with Sp-IgG_EPs+_ were more likely to be females. Notably, autoimmune diseases often exhibit a gender bias, with a higher prevalence among women at a ratio of 2 to 1 (47). However, further validation is necessary to confirm whether females with asthma are indeed more susceptible to airway auto-reactivity. Furthermore, patients with Sp-IgG_EPs+_ experienced a higher frequency of exacerbations and had more severe asthma. It has been postulated that chronic airway inflammation initiates an adaptive immune response, leading to the accumulation of various chemokines, including B-cell activating factors and B-cell chemoattractants. This process supports the formation of ectopic lymphoid structures, resulting in the local generation of Sp-IgG in the airways (48). Sp-IgG has been found to directly trigger eosinophil extracellular trap cell death (EETosis) (8). EETosis causes damage to the airway epithelium by releasing potent eosinophilic inflammatory mediators (49). The persistent inflammation can create a feedback loop, where inflammatory cytokines and other mediators continuously activate B cells, leading to sustained production of Sp-IgG and ongoing tissue damage. (The hypothesis of this vicious cycle is visually depicted in **Supplementary Figure 7**). To date, the specific mechanism by which Sp-IgG triggers EETosis remains unknown. In autoimmune diseases, Fc receptors, particularly Fc gamma receptors (FcγRs), and their interactions with autoantibodies and immune complexes, play a central role in autoimmune diseases (50). Evidence shows that low-density eosinophils are more correlated with the severity of asthma and express abundant FcγRs (51). Therefore, the upstream signaling pathway involving FcγRs in eosinophils might be responsible for Sp-IgG-induced EETosis. Moreover, our understanding of the association between the B cell system, production of Sp-IgG, and asthma is very limited. The mechanisms by which B cells secrete Sp-IgG include loss of B cell tolerance, B cell receptor signaling, genetic factors, defective regulatory mechanisms, cytokine dysregulation, and environmental triggers (52). Given that asthma is a chronic inflammatory disease, the most plausible explanation might be the excessive accumulation of inflammatory mediators in the airways, leading to an overactive B cell autoimmune response. Further experimental studies are needed to address this issue and elucidate the precise mechanisms involved.

Numerous risk factors are associated with severe asthma, and our investigation has identified Sp-IgG_EPs+_ as an independent risk factor contributing to the severity of this condition. However, the presence of Sp-IgG_EPs+_ alone does not offer sufficient predictive capability for severe asthma, potentially due to the substantial heterogeneity inherent to asthma. Yet, when combined with other clinical features, including a non-atopic history, advanced age (age > 45 years), and uncontrolled disease status (ACT < 15), Sp-IgG_EPs+_ demonstrates the ideal potential for predicting severe asthma. Based on our results, it is reasonable to assume that older individuals with non-allergic severe asthma and suboptimal disease control were more likely to manifest airway auto-reactivity. Indeed, a portion of patients with refractory severe asthma cannot be explained by the T1/T2 imbalance paradigm associated with allergies or chronic bacterial infections. Therefore, a comprehensive exploration of airway auto-reactivity may be necessary for this particular group of patients.

Strengths of our study include demonstrating a significant association between airway eosinophilic inflammation, elevated Sp-IgG levels, and asthma severity. The panel of Sp-IgG_EPs_ provided a comprehensive assessment of autoimmune events related to airway eosinophilic inflammation. Beyond proposing Sp-IgG_EPs_ as indicators of severity, our findings also enrich knowledge about the pathophysiology of asthma and pave the way for further studies targeting autoimmune responses in asthma, potentially identifying distinct autoimmune asthma endotypes. However, our current study has several limitations. Firstly, it included patients with cross-sectional assessments, which rendered us unable to evaluate the dynamic changes in Sp-IgG as well as Sp-IgG_EPs_ under regular and intensified treatments, including biological therapy. Consequently, we were unable to assess the long-term prognosis of airway auto-reactivity in asthma. Secondly, we could not conclude that there were no other types of specific Sp-IgG that exhibited stronger clinical relevance than our panel of Sp-IgG_EPs_ in asthma, particularly for those patients with neutrophilic and paucigranulocytic asthma. Further investigations are needed to explore the clinical relevance of Sp-IgG_EPs_ in different asthma phenotypes. Additionally, conducting a large-scale clinical study is necessary to establish the normal reference range of Sp-IgG_EPs_ and promote their clinical applications. Finally, although we found the important clinical relevance of Sp-IgG_EPs_ in asthma, we could not conclude the pathological functions of Sp-IgG_EPs_. Further experimental studies are warranted.

In conclusion, our findings illuminated a significant association between airway eosinophilic inflammation, elevated Sp-IgG, and risk of asthma. We identified the strong clinical relevance of Sp-IgG_EPs_ in asthma, which highlights its potential to serve specific biomarker reflecting hidden autoimmune events in the airways.

## Data availability statement

The original contributions presented in the study are included in the article/**Supplementary Material**. Further inquiries can be directed to the corresponding authors.

## Ethics statement

The studies involving humans were approved by the Ethics Review Board of the First Affiliated Hospital of Guangzhou Medical University (medical ethics year 2017, No. 25). The studies were conducted in accordance with the local legislation and institutional requirements. The participants provided their written informed consent to participate in this study.

## Author contributions

RQ: Formal analysis, Funding acquisition, Methodology, Project administration, Validation, Writing – original draft, Writing – review & editing. FL: Methodology, Project administration, Software, Supervision, Validation, Writing – original draft, Writing – review & editing. PZ: Investigation, Methodology, Project administration, Writing – original draft, Writing – review & editing. RH: Conceptualization, Data curation, Investigation, Project administration, Resources, Writing – original draft. HH: Investigation, Methodology, Project administration, Writing – original draft. YG: Investigation, Methodology, Project administration, Resources, Writing – original draft. ZZ: Conceptualization, Investigation, Methodology, Project administration, Software, Writing – original draft. JX: Methodology, Project administration, Software, Supervision, Writing – original draft. LH: Data curation, Project administration, Resources, Validation, Writing – original draft. TP: Investigation, Methodology, Project administration, Validation, Visualization, Writing – original draft, Writing – review & editing. JL: Data curation, Formal analysis, Funding acquisition, Investigation, Methodology, Project administration, Resources, Validation, Visualization, Writing – original draft, Writing – review & editing.
