# Supplementary material for: Presence of sputum IgG against eosinophilic inflammatory proteins in asthma
Source: Front Immunol. 2024 Jul 18;15:1423764. doi: 10.3389/fimmu.2024.1423764 (PMC11291201; doi:10.3389/fimmu.2024.1423764)
Supplement: Supplementary file 1 [file Image_1.pdf]

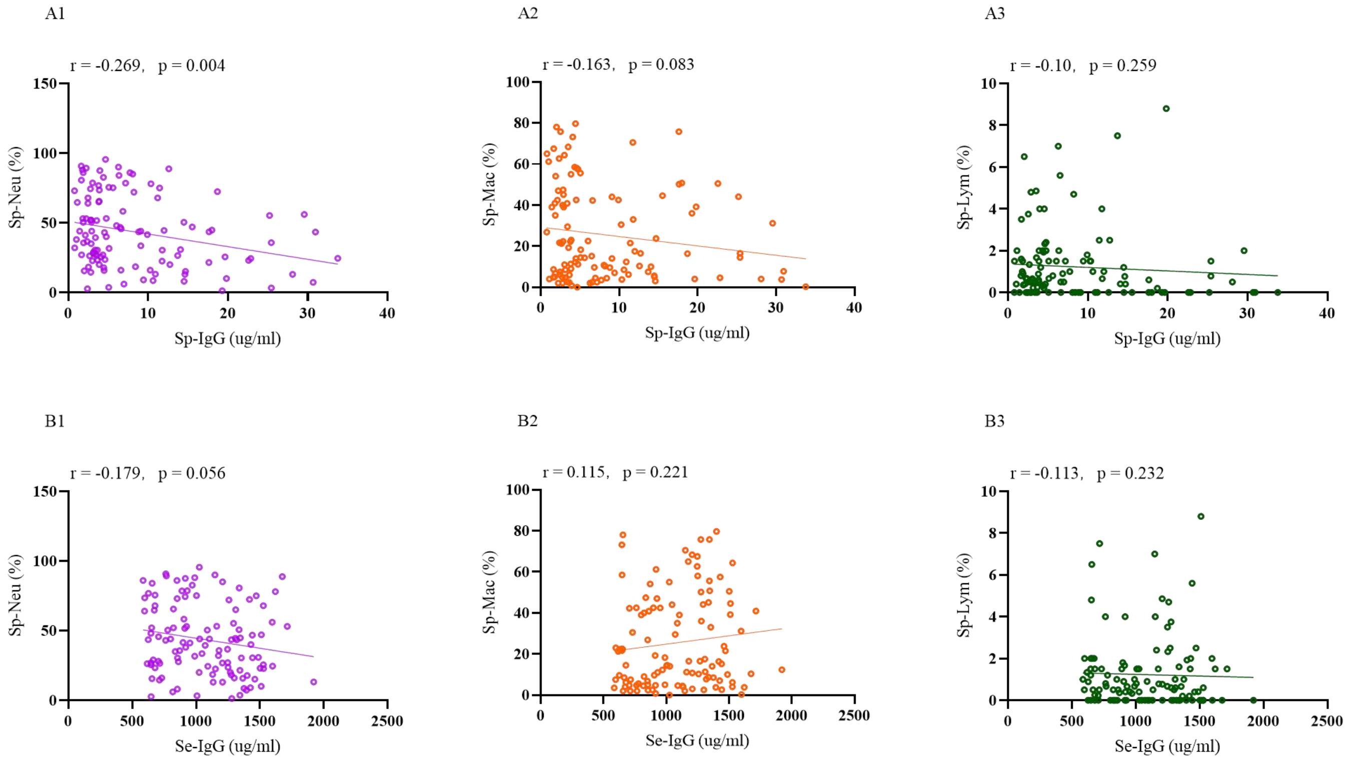

Supplementary Figure 1. Correlation between Sp-IgG and Se-IgG with Airway Inflammatory Cells. Panel A1-A3 depict the correlations between Sp-IgG and sputum neutrophil, macrophage, and lymphocyte counts, respectively. Panel B1-B3 illustrate the correlations between Se-IgG and sputum neutrophil, macrophage, and lymphocyte counts, respectively. Abbreviations: Sp: sputum; Se: serum; Neu: neutrophil; Mac: macrophage, Lym: lymphocyte.

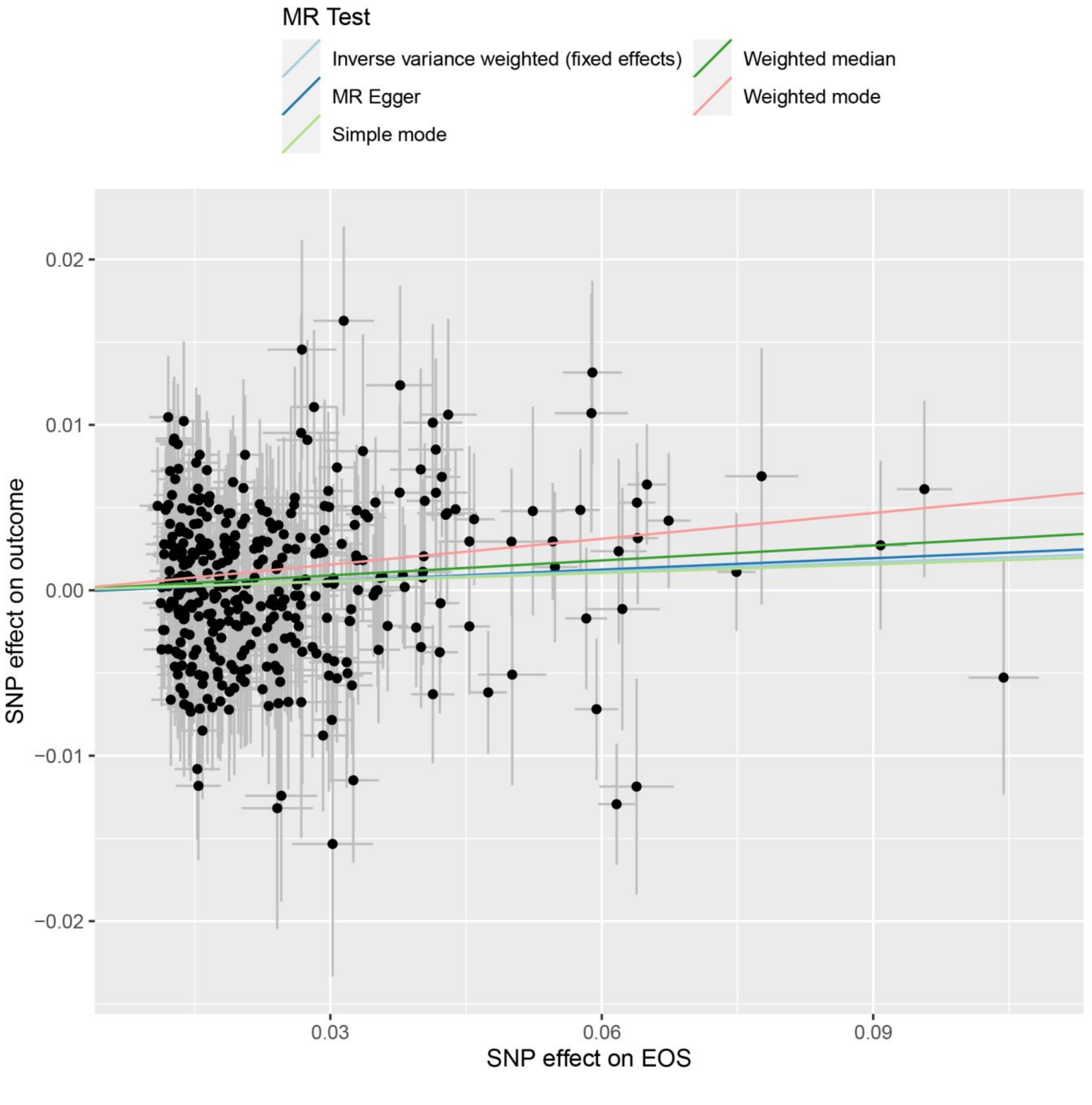

Supplementary Figure 2. Scatter plot of EOS-IgG.

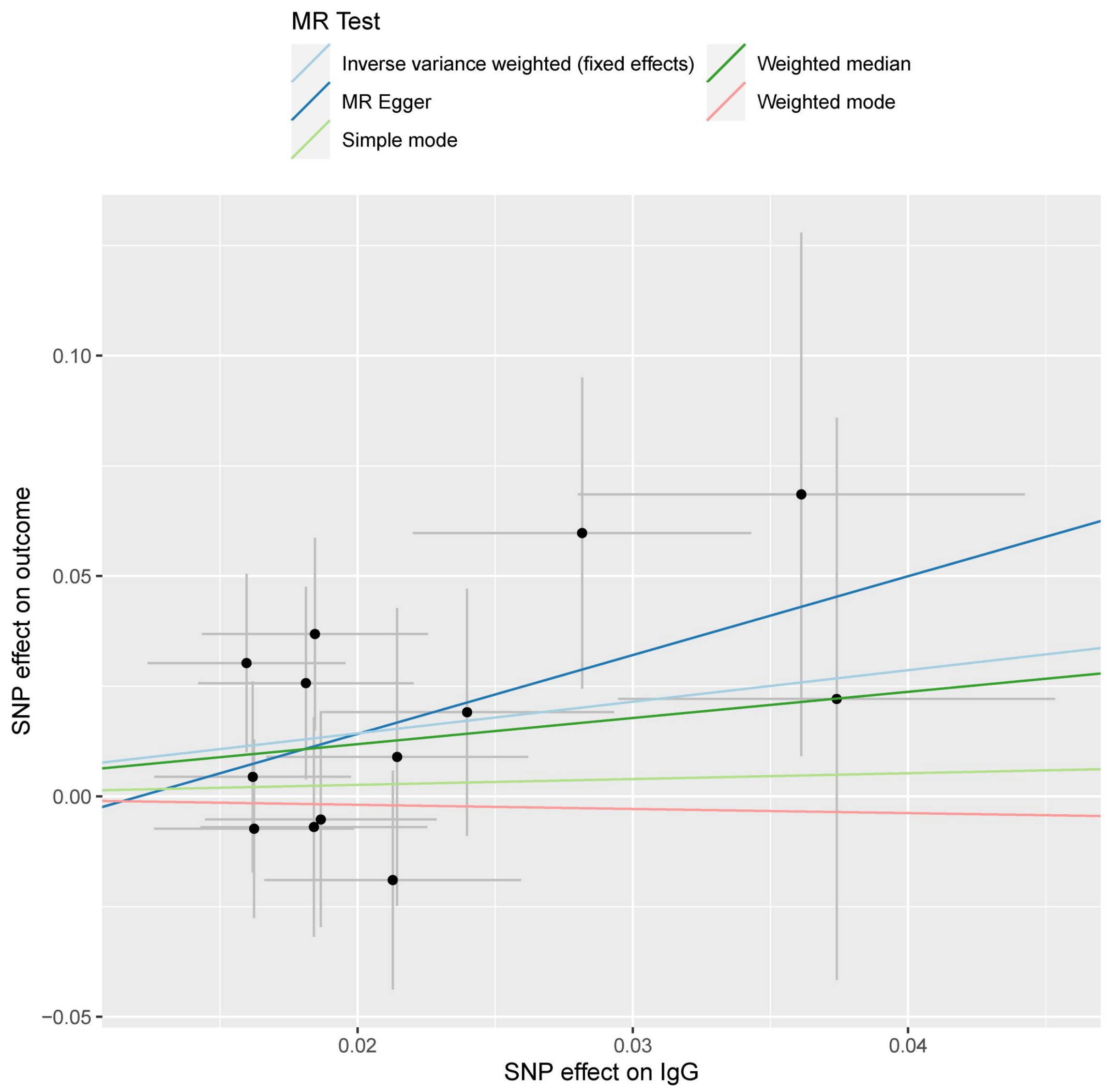

Supplementary Figure 3. Scatter plot of IgG-Asthma.

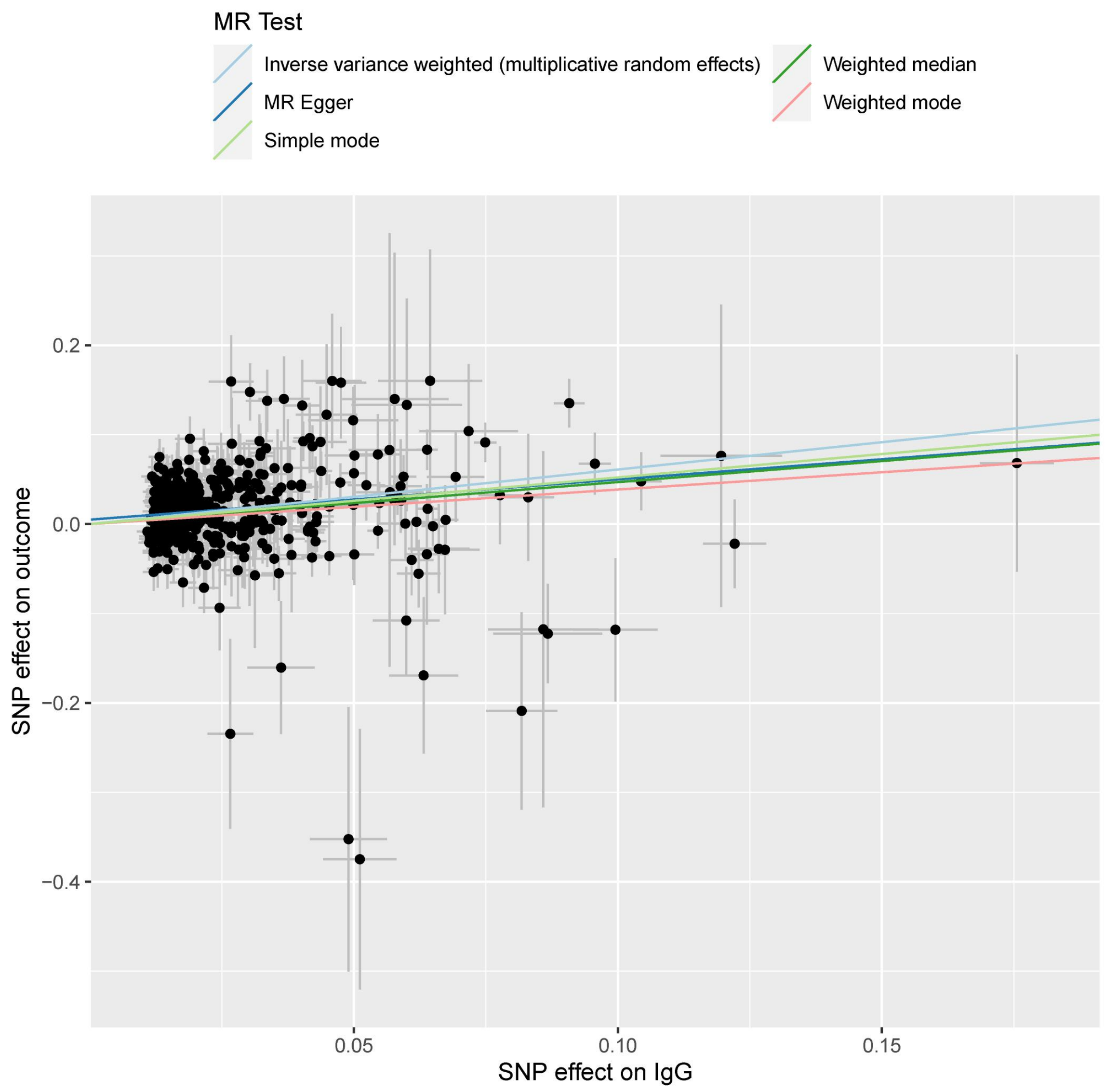

Supplementary Figure 4. Scatter plot of EOS-Asthma

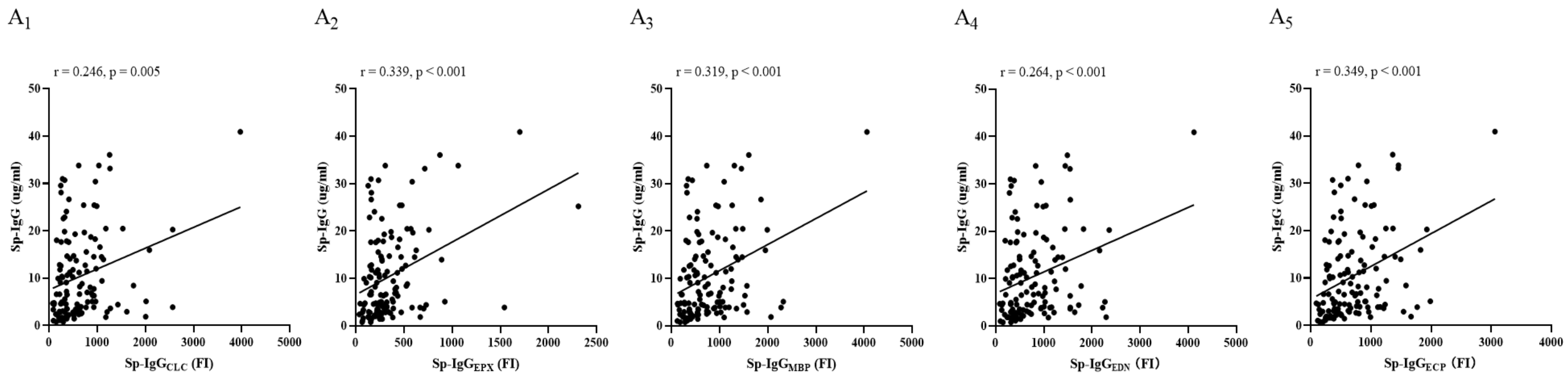

Supplementary Figure 5. Correlations between Sp-IgG$_{EPs}$ and Sp-IgG. Plot A1 to A5 depict the correlations between various sputum autoantibodies (Sp-IgG$_{EPs}$) and total sputum IgG (Sp-IgG). Abbreviations: Sp-IgG$_{ECP}$, sputum autoantibodies against eosinophil cationic protein; Sp-IgG$_{EDN}$, sputum autoantibodies against eosinophil-derived neurotoxin; Sp-IgG$_{MBP}$, sputum autoantibodies against eosinophil major basic protein; Sp-IgG$_{EPX}$, sputum autoantibodies against eosinophil peroxidase; Sp-IgG$_{CLC}$, sputum autoantibodies against Charcot-Leyden Crystal protein; Se-IgG: serum autoantibodies.

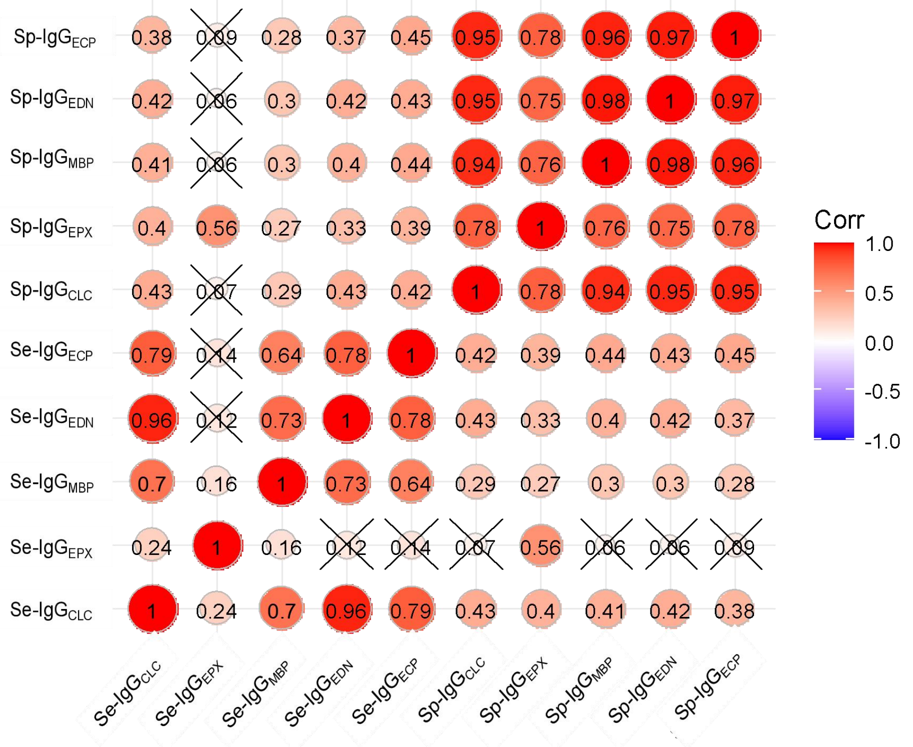

Supplementary Figure 6. Correlation Matrix of Autoantibodies. The numbers inside each circle represent the correlation coefficient between different autoantibodies. Figures with a cross indicate correlations that lack statistical significance. Sp-IgG$_{ECP}$, sputum autoantibodies against eosinophil cationic protein; Sp-IgG$_{EDN}$, sputum autoantibodies against eosinophil-derived neurotoxin; Sp-IgG$_{MBP}$, sputum autoantibodies against eosinophil major basic protein; Sp-IgG$_{EPX}$, sputum autoantibodies against eosinophil peroxidase; Sp-IgG$_{CLC}$, sputum autoantibodies against Charcot-Leyden Crystal protein; Se-IgG: serum autoantibodies.

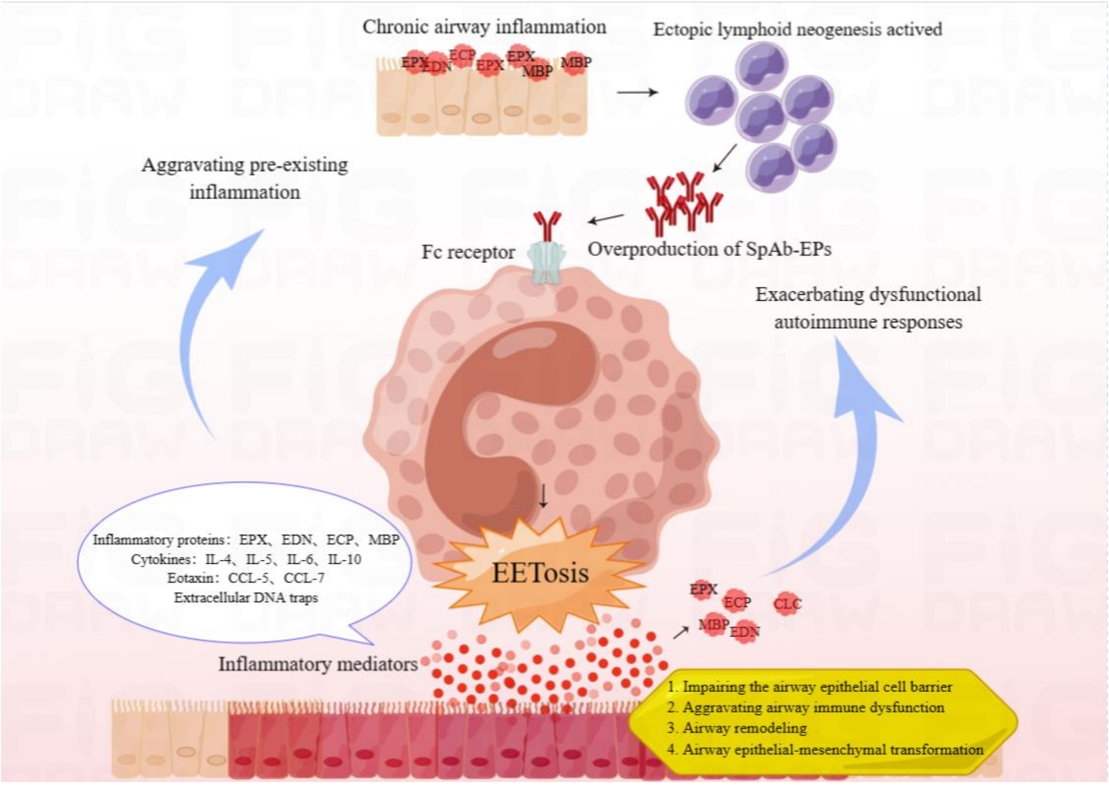

Supplementary Figure 7. The hypothesis of a vicious cycle driven by dysfunctional autoimmune responses in asthma. During chronic eosinophilic inflammation in the airways, bronchial ectopic B lymphocytes become activated and produce IgG autoantibodies against eosinophil-released proteins present (Sp-IgG_{EPs}) in the airways. The accumulation of these autoantibodies results in their binding to eosinophils through Fc receptors on the cell surface, initiating the process known as eosinophil extracellular DNA trap cell death (EETosis). EETosis, in turn, leads to the release of a substantial quantity of inflammatory mediators. This exacerbates the pre-existing inflammation, disrupts the balance of the airway immune system, impairs the airway epithelial cell barrier, intensifies the airway epithelial-mesenchymal transformation, and contributes to airway remodeling. These processes collectively play integral roles in the pathogenesis and progression of severe eosinophilic asthma.
