# Supplementary material for: Presence of sputum IgG against eosinophilic inflammatory proteins in asthma
Source: Front Immunol. 2024 Jul 18;15:1423764. doi: 10.3389/fimmu.2024.1423764 (PMC11291201; doi:10.3389/fimmu.2024.1423764)
Supplement: Supplementary file 2 [file Table_1.docx]

Supplementary Table 1: Characteristics of data sources of exposure and outcome included in our study.

| Exposures or outcome | GWAS ID | Identified SNPs | Participants included in analysis | Source |
| --- | --- | --- | --- | --- |
| Eosinophil cell count | ieu-b-33 | 371 | 563946 European | https://gwas.mrcieu.ac.uk/datasets/ieu-b-33/ |
| IgG levels | ebi-a-GCST006357 | 13 | 1000 European | https://gwas.mrcieu.ac.uk/datasets/ebi-a-GCST006357/ |
| Asthma | NA | NA | 230909 European (5206 cases and 225703 controls) | https://storage.googleapis.com/finngen-public-data-r10/summary_stats/finngen_R10_ASTHMA_ALLERG.gz |

| Supplementary Table 2 . MR estimates the causal relationships between eosinophil, IgG, and asthma. | | | | |
| --- | --- | --- | --- | --- |
| Step 1 | | | | |
| Exposure: eosinophil (sample size = 563946) | No.SNPs | OR | 95% CI | p |
| Outcome: IgG (sample size = 1000) |  |  |  |  |
| IVW | 371 | 1.02 | 1.00-1.04 | 0.020 |
| MR Egger | 371 | 1.02 | 0.99-1.06 | 0.197 |
| Weighted median | 371 | 1.03 | 1.00-1.06 | 0.020 |
| Weighted mode | 371 | 1.05 | 1.01-1.09 | 0.009 |
| Simple mode | 371 | 1.02 | 0.95-1.09 | 0.624 |
| Step 2 | | | | |
| Exposure: IgG ( sample size = 1000) | No.SNPs | OR | 95% CI | p |
| Outcome: asthma (n =5206 cases and 225703 control participants) |  |  |  |  |
| IVW | 13 | 2.05 | 1.00-4.17 | 0.049 |
| MR Egger | 13 | 5.98 | 0.21-173.54 | 0.320 |
| Weighted median | 13 | 1.81 | 0.66-4.93 | 0.246 |
| Weighted mode | 13 | 0.91 | 0.14-6.03 | 0.924 |
| Simple mode | 13 | 1.14 | 0.19-6.76 | 0.887 |

##

Abbreviations: IgG: immunoglobulin G; IVW: inverse variance weighting; SNPs: single-nucleotide polymorphisms; OR: odds ratio; CI: confidence interval

## Supplementary Table 3: Characteristics of SNPs used as instrumental variables for eosinophil cell count (EOS-IgG).

|  | SNPs | effect_allele | other_allele | eaf | beta | se | p | F-Statistic |
| --- | --- | --- | --- | --- | --- | --- | --- | --- |
| 1 | rs1004870 | T | C | 0.589 | 0.026 | 0.002 | 1.270E-38 | 169.025 |
| 2 | rs10059018 | T | G | 0.201 | -0.023 | 0.002 | 3.380E-21 | 89.428 |
| 3 | rs10062687 | G | T | 0.233 | 0.024 | 0.002 | 9.471E-25 | 105.654 |
| 4 | rs10100356 | A | G | 0.227 | -0.013 | 0.002 | 1.910E-08 | 31.618 |
| 5 | rs10165678 | A | G | 0.758 | -0.015 | 0.002 | 1.900E-10 | 40.639 |
| 6 | rs10174238 | A | G | 0.766 | 0.013 | 0.002 | 3.410E-08 | 30.498 |
| 7 | rs10195713 | T | C | 0.864 | 0.025 | 0.003 | 2.740E-17 | 71.619 |
| 8 | rs1036332 | C | A | 0.737 | -0.033 | 0.002 | 1.610E-47 | 209.857 |
| 9 | rs1037674 | T | G | 0.292 | 0.013 | 0.002 | 1.630E-09 | 36.418 |
| 10 | rs1039341 | T | C | 0.311 | -0.024 | 0.002 | 8.790E-29 | 124.044 |
| 11 | rs10472984 | G | C | 0.340 | -0.035 | 0.002 | 7.621E-63 | 280.201 |
| 12 | rs1047891 | A | C | 0.315 | 0.017 | 0.002 | 1.550E-15 | 63.632 |
| 13 | rs1057258 | T | C | 0.178 | -0.032 | 0.003 | 2.520E-35 | 153.974 |
| 14 | rs1059091 | G | A | 0.321 | 0.034 | 0.002 | 3.080E-56 | 249.834 |
| 15 | rs10745763 | T | G | 0.423 | 0.013 | 0.002 | 2.410E-11 | 44.687 |
| 16 | rs10777378 | A | G | 0.536 | -0.022 | 0.002 | 1.280E-29 | 127.829 |
| 17 | rs10782957 | T | C | 0.622 | 0.023 | 0.002 | 2.810E-28 | 121.820 |
| 18 | rs10876550 | A | G | 0.559 | 0.014 | 0.002 | 4.920E-12 | 47.781 |
| 19 | rs10900595 | A | C | 0.713 | 0.015 | 0.002 | 1.590E-12 | 50.022 |
| 20 | rs10930337 | T | C | 0.285 | 0.015 | 0.002 | 2.030E-11 | 45.034 |
| 21 | rs10962640 | G | C | 0.237 | 0.013 | 0.002 | 2.310E-08 | 31.273 |
| 22 | rs1099448 | T | C | 0.466 | 0.018 | 0.002 | 8.320E-19 | 78.497 |
| 23 | rs10995240 | C | G | 0.368 | -0.044 | 0.002 | 1.549E-101 | 457.737 |
| 24 | rs11065822 | T | G | 0.354 | 0.065 | 0.002 | 1.000E-200 | 925.739 |
| 25 | rs11071528 | C | G | 0.807 | 0.014 | 0.003 | 1.340E-08 | 32.304 |
| 26 | rs11071559 | T | C | 0.131 | -0.022 | 0.003 | 4.940E-14 | 56.824 |
| 27 | rs11079340 | C | T | 0.322 | 0.012 | 0.002 | 7.490E-09 | 33.472 |
| 28 | rs11088236 | T | C | 0.453 | 0.018 | 0.002 | 5.170E-20 | 84.037 |
| 29 | rs111759324 | T | C | 0.111 | -0.043 | 0.003 | 3.560E-42 | 185.373 |
| 30 | rs112036266 | T | C | 0.176 | 0.017 | 0.003 | 1.480E-10 | 41.134 |
| 31 | rs11204682 | T | G | 0.223 | -0.041 | 0.002 | 4.890E-67 | 299.558 |
| 32 | rs11236813 | C | G | 0.102 | -0.030 | 0.003 | 4.780E-20 | 84.205 |
| 33 | rs11255507 | G | T | 0.178 | 0.020 | 0.003 | 1.050E-14 | 59.875 |
| 34 | rs113105190 | C | T | 0.068 | -0.033 | 0.004 | 5.190E-17 | 70.369 |
| 35 | rs113542380 | A | G | 0.075 | -0.027 | 0.004 | 1.550E-12 | 50.049 |
| 36 | rs11555542 | C | T | 0.063 | 0.064 | 0.004 | 2.120E-54 | 241.503 |
| 37 | rs11571404 | T | C | 0.203 | 0.015 | 0.002 | 5.570E-10 | 38.525 |
| 38 | rs11578794 | T | A | 0.728 | -0.014 | 0.002 | 1.530E-09 | 36.549 |
| 39 | rs11628569 | G | A | 0.290 | -0.017 | 0.002 | 4.700E-14 | 56.955 |
| 40 | rs11647946 | A | G | 0.708 | -0.013 | 0.002 | 1.200E-09 | 37.034 |
| 41 | rs11684770 | G | A | 0.270 | -0.015 | 0.002 | 3.100E-11 | 44.173 |
| 42 | rs11695281 | T | C | 0.514 | -0.023 | 0.002 | 4.090E-31 | 134.665 |
| 43 | rs11701475 | C | T | 0.223 | 0.015 | 0.002 | 1.060E-09 | 37.264 |
| 44 | rs11702918 | T | C | 0.103 | -0.022 | 0.003 | 1.060E-11 | 46.290 |
| 45 | rs1170439 | C | T | 0.779 | 0.023 | 0.002 | 7.860E-21 | 87.757 |
| 46 | rs117068593 | T | C | 0.190 | -0.026 | 0.003 | 1.170E-24 | 105.266 |
| 47 | rs1178016 | T | C | 0.495 | 0.011 | 0.002 | 1.170E-08 | 32.582 |
| 48 | rs11786536 | A | G | 0.165 | -0.027 | 0.003 | 5.110E-23 | 97.765 |
| 49 | rs117955557 | T | G | 0.256 | -0.030 | 0.002 | 1.440E-40 | 178.070 |
| 50 | rs118013485 | A | G | 0.067 | -0.034 | 0.004 | 1.540E-16 | 68.229 |
| 51 | rs11869228 | T | C | 0.841 | 0.021 | 0.003 | 3.450E-14 | 57.557 |
| 52 | rs11886928 | G | T | 0.308 | -0.014 | 0.002 | 1.860E-11 | 45.197 |
| 53 | rs11931711 | T | C | 0.282 | 0.019 | 0.002 | 3.480E-18 | 75.689 |
| 54 | rs12100034 | A | G | 0.359 | -0.017 | 0.002 | 2.600E-16 | 67.218 |
| 55 | rs12154498 | C | A | 0.854 | -0.033 | 0.003 | 7.761E-31 | 133.448 |
| 56 | rs121564 | T | G | 0.270 | -0.017 | 0.002 | 1.260E-13 | 55.024 |
| 57 | rs12208103 | T | C | 0.378 | -0.031 | 0.002 | 6.830E-50 | 220.685 |
| 58 | rs12361586 | G | A | 0.146 | -0.015 | 0.003 | 2.480E-08 | 31.137 |
| 59 | rs12408934 | A | G | 0.103 | -0.041 | 0.003 | 2.360E-36 | 158.690 |
| 60 | rs12470046 | C | T | 0.319 | -0.012 | 0.002 | 6.930E-09 | 33.613 |
| 61 | rs12487980 | A | C | 0.634 | 0.013 | 0.002 | 1.500E-09 | 36.587 |
| 62 | rs12530946 | G | A | 0.613 | 0.043 | 0.002 | 3.990E-97 | 437.532 |
| 63 | rs12540285 | G | A | 0.221 | 0.015 | 0.002 | 1.010E-09 | 37.365 |
| 64 | rs12545733 | C | T | 0.703 | -0.031 | 0.002 | 8.900E-45 | 197.294 |
| 65 | rs1257192 | G | A | 0.812 | 0.015 | 0.003 | 8.810E-09 | 33.147 |
| 66 | rs12581511 | G | C | 0.173 | -0.018 | 0.003 | 2.070E-11 | 44.968 |
| 67 | rs12681644 | T | C | 0.212 | -0.019 | 0.002 | 1.230E-15 | 64.125 |
| 68 | rs12705849 | A | G | 0.407 | -0.021 | 0.002 | 2.550E-24 | 103.664 |
| 69 | rs12820863 | T | C | 0.352 | 0.022 | 0.002 | 1.070E-25 | 110.005 |
| 70 | rs12878610 | C | T | 0.476 | -0.013 | 0.002 | 3.950E-11 | 43.710 |
| 71 | rs12882281 | C | T | 0.239 | -0.014 | 0.002 | 7.370E-09 | 33.492 |
| 72 | rs12941068 | A | G | 0.286 | -0.014 | 0.002 | 9.590E-10 | 37.456 |
| 73 | rs1296535 | T | C | 0.777 | -0.019 | 0.002 | 3.350E-15 | 62.134 |
| 74 | rs12978850 | T | C | 0.194 | -0.014 | 0.003 | 3.990E-08 | 30.206 |
| 75 | rs13073683 | C | T | 0.399 | 0.016 | 0.002 | 1.320E-14 | 59.422 |
| 76 | rs13105682 | G | T | 0.060 | -0.027 | 0.004 | 8.220E-10 | 37.770 |
| 77 | rs13120371 | G | A | 0.327 | 0.015 | 0.002 | 3.000E-13 | 53.273 |
| 78 | rs13138355 | T | C | 0.186 | -0.055 | 0.003 | 2.350E-102 | 461.852 |
| 79 | rs13139941 | G | A | 0.803 | -0.014 | 0.002 | 2.840E-08 | 30.869 |
| 80 | rs13207791 | G | A | 0.089 | 0.026 | 0.004 | 4.300E-13 | 52.567 |
| 81 | rs13226583 | T | A | 0.116 | -0.062 | 0.003 | 1.590E-88 | 398.258 |
| 82 | rs1323650 | T | G | 0.681 | -0.014 | 0.002 | 2.530E-10 | 40.058 |
| 83 | rs13511 | C | T | 0.577 | -0.016 | 0.002 | 4.130E-16 | 66.273 |
| 84 | rs1353286 | G | T | 0.453 | 0.020 | 0.002 | 3.090E-24 | 103.303 |
| 85 | rs1365623 | T | C | 0.372 | 0.012 | 0.002 | 1.710E-08 | 31.857 |
| 86 | rs1395269 | G | T | 0.301 | -0.024 | 0.002 | 1.060E-27 | 119.155 |
| 87 | rs139640694 | G | A | 0.093 | 0.025 | 0.003 | 3.540E-13 | 52.967 |
| 88 | rs1406449 | C | T | 0.462 | -0.012 | 0.002 | 8.690E-10 | 37.662 |
| 89 | rs1414517 | G | C | 0.187 | -0.025 | 0.003 | 1.360E-22 | 95.789 |
| 90 | rs1427499 | G | A | 0.710 | 0.019 | 0.002 | 1.300E-17 | 73.113 |
| 91 | rs144569746 | T | C | 0.102 | 0.059 | 0.003 | 4.230E-72 | 322.760 |
| 92 | rs1448187 | C | T | 0.700 | 0.016 | 0.002 | 7.890E-12 | 46.877 |
| 93 | rs14713 | A | G | 0.205 | -0.014 | 0.002 | 3.530E-08 | 30.438 |
| 94 | rs1471816 | A | G | 0.503 | 0.011 | 0.002 | 3.940E-08 | 30.214 |
| 95 | rs1479918 | T | A | 0.260 | -0.019 | 0.002 | 1.050E-16 | 68.961 |
| 96 | rs1519602 | G | T | 0.648 | 0.013 | 0.002 | 7.430E-10 | 37.951 |
| 97 | rs1529745 | G | C | 0.175 | 0.016 | 0.003 | 2.900E-09 | 35.302 |
| 98 | rs1539174 | G | C | 0.237 | 0.038 | 0.002 | 1.120E-59 | 265.764 |
| 99 | rs1547258 | C | T | 0.708 | -0.019 | 0.002 | 1.490E-18 | 77.408 |
| 100 | rs159963 | A | C | 0.582 | 0.019 | 0.002 | 9.881E-22 | 91.844 |
| 101 | rs1672753 | T | C | 0.811 | -0.017 | 0.003 | 6.741E-12 | 47.174 |
| 102 | rs1684578 | T | G | 0.416 | 0.016 | 0.002 | 5.150E-16 | 65.805 |
| 103 | rs1689510 | C | G | 0.338 | 0.027 | 0.002 | 1.060E-36 | 160.287 |
| 104 | rs16956811 | G | T | 0.081 | -0.029 | 0.004 | 1.750E-15 | 63.428 |
| 105 | rs17061503 | A | G | 0.307 | 0.036 | 0.002 | 8.041E-61 | 270.791 |
| 106 | rs17175830 | A | G | 0.238 | 0.032 | 0.002 | 4.130E-42 | 185.132 |
| 107 | rs17293632 | T | C | 0.236 | 0.030 | 0.002 | 8.921E-37 | 160.639 |
| 108 | rs174548 | G | C | 0.314 | -0.023 | 0.002 | 4.080E-27 | 116.421 |
| 109 | rs17482472 | A | G | 0.099 | -0.031 | 0.003 | 5.339E-21 | 88.520 |
| 110 | rs17516457 | C | T | 0.417 | -0.064 | 0.002 | 1.000E-200 | 1012.110 |
| 111 | rs175705 | G | C | 0.719 | 0.038 | 0.002 | 8.800E-67 | 298.275 |
| 112 | rs17653687 | G | A | 0.179 | -0.018 | 0.003 | 4.060E-12 | 48.165 |
| 113 | rs17668272 | T | G | 0.118 | -0.042 | 0.003 | 7.480E-42 | 183.870 |
| 114 | rs17682575 | T | C | 0.201 | -0.018 | 0.002 | 1.330E-12 | 50.349 |
| 115 | rs17689159 | C | T | 0.315 | -0.014 | 0.002 | 1.400E-11 | 45.740 |
| 116 | rs17849501 | T | C | 0.052 | -0.027 | 0.004 | 2.100E-09 | 35.933 |
| 117 | rs1800692 | G | A | 0.588 | -0.018 | 0.002 | 8.921E-19 | 78.373 |
| 118 | rs180506 | A | G | 0.776 | -0.023 | 0.002 | 5.820E-22 | 92.918 |
| 119 | rs1828803 | A | C | 0.392 | 0.013 | 0.002 | 1.280E-10 | 41.388 |
| 120 | rs1861489 | A | T | 0.782 | 0.013 | 0.002 | 4.140E-08 | 30.136 |
| 121 | rs201798 | A | G | 0.615 | 0.020 | 0.002 | 5.710E-23 | 97.510 |
| 122 | rs2025489 | G | A | 0.526 | 0.016 | 0.002 | 2.420E-15 | 62.791 |
| 123 | rs2089979 | G | A | 0.416 | -0.015 | 0.002 | 1.250E-13 | 55.016 |
| 124 | rs214080 | G | A | 0.580 | 0.013 | 0.002 | 1.370E-10 | 41.282 |
| 125 | rs2182885 | A | G | 0.605 | 0.026 | 0.002 | 2.050E-38 | 168.181 |
| 126 | rs2223043 | G | A | 0.307 | 0.019 | 0.002 | 3.230E-17 | 71.323 |
| 127 | rs2228467 | C | T | 0.062 | 0.062 | 0.004 | 8.831E-52 | 229.450 |
| 128 | rs2239633 | A | G | 0.484 | 0.035 | 0.002 | 1.500E-68 | 306.531 |
| 129 | rs2253427 | C | T | 0.778 | -0.014 | 0.002 | 8.680E-09 | 33.169 |
| 130 | rs2399441 | C | T | 0.351 | -0.019 | 0.002 | 8.080E-20 | 83.163 |
| 131 | rs2410732 | A | C | 0.760 | 0.014 | 0.002 | 5.540E-09 | 34.035 |
| 132 | rs2419313 | A | G | 0.143 | -0.021 | 0.003 | 5.210E-13 | 52.193 |
| 133 | rs2431097 | T | C | 0.486 | 0.015 | 0.002 | 5.950E-14 | 56.479 |
| 134 | rs2497318 | T | C | 0.443 | -0.030 | 0.002 | 1.960E-49 | 218.570 |
| 135 | rs2502995 | C | T | 0.570 | 0.022 | 0.002 | 1.060E-27 | 119.149 |
| 136 | rs2505521 | T | C | 0.829 | -0.015 | 0.003 | 1.450E-08 | 32.165 |
| 137 | rs2566133 | C | T | 0.584 | -0.014 | 0.002 | 1.060E-12 | 50.823 |
| 138 | rs2579505 | C | T | 0.638 | 0.042 | 0.002 | 5.790E-94 | 423.333 |
| 139 | rs2646438 | A | G | 0.566 | -0.021 | 0.002 | 1.200E-24 | 105.121 |
| 140 | rs2713548 | T | C | 0.633 | 0.013 | 0.002 | 1.020E-09 | 37.355 |
| 141 | rs2788211 | C | T | 0.817 | 0.016 | 0.003 | 4.790E-10 | 38.809 |
| 142 | rs2807740 | T | C | 0.770 | 0.035 | 0.002 | 1.050E-50 | 224.427 |
| 143 | rs2817377 | A | G | 0.537 | -0.012 | 0.002 | 2.920E-08 | 30.817 |
| 144 | rs28362902 | A | G | 0.133 | 0.017 | 0.003 | 1.660E-08 | 31.908 |
| 145 | rs2838317 | C | A | 0.550 | 0.013 | 0.002 | 1.200E-10 | 41.549 |
| 146 | rs28383314 | C | T | 0.624 | 0.062 | 0.002 | 6.383E-199 | 905.718 |
| 147 | rs28421324 | T | A | 0.102 | 0.036 | 0.003 | 1.570E-27 | 118.334 |
| 148 | rs2847266 | T | C | 0.717 | -0.014 | 0.002 | 1.220E-10 | 41.494 |
| 149 | rs2850542 | T | G | 0.556 | -0.013 | 0.002 | 6.500E-11 | 42.740 |
| 150 | rs28532037 | A | G | 0.907 | -0.030 | 0.003 | 4.190E-18 | 75.353 |
| 151 | rs2864936 | C | A | 0.805 | -0.014 | 0.003 | 1.710E-08 | 31.861 |
| 152 | rs2887502 | T | C | 0.590 | -0.014 | 0.002 | 1.740E-11 | 45.289 |
| 153 | rs2894401 | G | A | 0.705 | -0.021 | 0.002 | 6.160E-21 | 88.215 |
| 154 | rs290430 | T | C | 0.345 | -0.012 | 0.002 | 8.310E-09 | 33.253 |
| 155 | rs2920505 | A | G | 0.602 | -0.030 | 0.002 | 9.469E-49 | 215.575 |
| 156 | rs295 | C | A | 0.235 | 0.016 | 0.002 | 1.160E-11 | 46.108 |
| 157 | rs295273 | A | G | 0.256 | 0.021 | 0.002 | 1.500E-19 | 81.897 |
| 158 | rs2979489 | A | G | 0.742 | 0.014 | 0.002 | 1.330E-09 | 36.831 |
| 159 | rs2992333 | A | G | 0.599 | -0.034 | 0.002 | 1.350E-63 | 283.609 |
| 160 | rs301162 | G | A | 0.853 | 0.026 | 0.003 | 1.160E-20 | 86.962 |
| 161 | rs3024971 | G | T | 0.107 | -0.040 | 0.003 | 5.040E-36 | 157.182 |
| 162 | rs3093023 | A | G | 0.434 | 0.013 | 0.002 | 2.270E-10 | 40.257 |
| 163 | rs3096309 | T | C | 0.824 | 0.015 | 0.003 | 1.360E-08 | 32.304 |
| 164 | rs3110791 | C | T | 0.623 | 0.015 | 0.002 | 7.490E-14 | 56.006 |
| 165 | rs3218148 | A | G | 0.540 | -0.018 | 0.002 | 7.079E-19 | 78.836 |
| 166 | rs33982662 | A | C | 0.285 | 0.020 | 0.002 | 1.190E-20 | 86.909 |
| 167 | rs34212866 | G | A | 0.221 | 0.020 | 0.002 | 5.740E-17 | 70.187 |
| 168 | rs34290285 | A | G | 0.257 | -0.046 | 0.002 | 4.730E-91 | 409.690 |
| 169 | rs34363176 | G | C | 0.244 | 0.013 | 0.002 | 8.430E-09 | 33.238 |
| 170 | rs34448954 | T | C | 0.106 | -0.025 | 0.003 | 3.870E-15 | 61.851 |
| 171 | rs34466956 | T | C | 0.568 | -0.014 | 0.002 | 2.600E-11 | 44.532 |
| 172 | rs34495 | T | G | 0.304 | -0.020 | 0.002 | 3.670E-20 | 84.674 |
| 173 | rs34505104 | G | A | 0.306 | -0.026 | 0.002 | 1.220E-33 | 146.309 |
| 174 | rs34631302 | G | A | 0.645 | -0.012 | 0.002 | 9.660E-09 | 32.963 |
| 175 | rs346835 | T | C | 0.328 | -0.027 | 0.002 | 7.549E-37 | 160.918 |
| 176 | rs350836 | T | C | 0.710 | -0.017 | 0.002 | 6.411E-15 | 60.854 |
| 177 | rs35249183 | G | A | 0.100 | 0.040 | 0.003 | 1.460E-32 | 141.322 |
| 178 | rs35409523 | A | G | 0.076 | 0.050 | 0.004 | 1.430E-40 | 178.080 |
| 179 | rs36084354 | A | G | 0.092 | -0.045 | 0.003 | 1.170E-39 | 173.824 |
| 180 | rs3731211 | A | T | 0.720 | 0.018 | 0.002 | 2.340E-15 | 62.855 |
| 181 | rs3742704 | C | A | 0.092 | 0.019 | 0.003 | 1.540E-08 | 32.048 |
| 182 | rs3746420 | C | G | 0.061 | -0.032 | 0.004 | 2.260E-14 | 58.368 |
| 183 | rs3747869 | C | A | 0.901 | 0.020 | 0.003 | 2.310E-09 | 35.743 |
| 184 | rs3757114 | C | A | 0.474 | -0.025 | 0.002 | 6.150E-37 | 161.367 |
| 185 | rs3785356 | T | C | 0.298 | 0.030 | 0.002 | 1.700E-43 | 191.504 |
| 186 | rs3786586 | G | A | 0.155 | 0.032 | 0.003 | 4.420E-31 | 134.593 |
| 187 | rs3790163 | G | A | 0.792 | -0.015 | 0.003 | 1.350E-09 | 36.794 |
| 188 | rs3804590 | T | G | 0.317 | 0.018 | 0.002 | 3.760E-17 | 70.988 |
| 189 | rs3812206 | T | C | 0.300 | -0.012 | 0.002 | 2.070E-08 | 31.485 |
| 190 | rs3823536 | A | G | 0.467 | -0.018 | 0.002 | 3.160E-19 | 80.493 |
| 191 | rs3824867 | G | A | 0.712 | 0.017 | 0.002 | 1.770E-14 | 58.868 |
| 192 | rs3846855 | A | G | 0.183 | 0.028 | 0.003 | 5.010E-28 | 120.631 |
| 193 | rs3850107 | G | C | 0.294 | -0.014 | 0.002 | 3.940E-10 | 39.210 |
| 194 | rs3950296 | G | C | 0.244 | 0.024 | 0.002 | 8.300E-25 | 105.900 |
| 195 | rs397187 | C | T | 0.430 | -0.012 | 0.002 | 1.280E-09 | 36.890 |
| 196 | rs4074672 | T | C | 0.369 | 0.015 | 0.002 | 1.480E-12 | 50.140 |
| 197 | rs410867 | G | A | 0.217 | -0.059 | 0.002 | 4.121E-134 | 607.773 |
| 198 | rs412884 | C | T | 0.672 | 0.058 | 0.002 | 2.218E-164 | 746.797 |
| 199 | rs4142528 | A | T | 0.672 | -0.075 | 0.002 | 1.000E-200 | 1247.717 |
| 200 | rs4148757 | C | A | 0.210 | -0.017 | 0.002 | 2.240E-12 | 49.345 |
| 201 | rs4240624 | A | G | 0.909 | -0.020 | 0.003 | 3.620E-09 | 34.872 |
| 202 | rs4280242 | T | C | 0.754 | -0.033 | 0.002 | 1.030E-45 | 201.607 |
| 203 | rs4310436 | A | G | 0.120 | 0.019 | 0.003 | 9.150E-10 | 37.563 |
| 204 | rs4347868 | A | G | 0.238 | 0.013 | 0.002 | 1.690E-08 | 31.873 |
| 205 | rs4409785 | C | T | 0.172 | 0.016 | 0.003 | 4.250E-10 | 39.050 |
| 206 | rs460631 | G | A | 0.885 | 0.026 | 0.003 | 1.880E-16 | 67.847 |
| 207 | rs4618204 | C | T | 0.444 | 0.018 | 0.002 | 1.090E-19 | 82.572 |
| 208 | rs4652560 | T | A | 0.618 | -0.015 | 0.002 | 5.230E-14 | 56.743 |
| 209 | rs4675190 | T | C | 0.591 | -0.012 | 0.002 | 7.570E-09 | 33.453 |
| 210 | rs4680250 | G | C | 0.693 | -0.016 | 0.002 | 4.850E-13 | 52.331 |
| 211 | rs4703589 | C | T | 0.533 | 0.014 | 0.002 | 3.260E-13 | 53.107 |
| 212 | rs4703730 | T | C | 0.517 | -0.016 | 0.002 | 6.910E-16 | 65.248 |
| 213 | rs4721559 | T | C | 0.278 | -0.017 | 0.002 | 6.560E-15 | 60.831 |
| 214 | rs4722171 | G | A | 0.592 | -0.027 | 0.002 | 1.830E-41 | 182.202 |
| 215 | rs473739 | G | T | 0.319 | -0.014 | 0.002 | 7.070E-11 | 42.556 |
| 216 | rs4746153 | C | G | 0.185 | 0.016 | 0.003 | 8.580E-10 | 37.669 |
| 217 | rs4849903 | T | C | 0.633 | 0.021 | 0.002 | 9.949E-25 | 105.526 |
| 218 | rs4870977 | C | G | 0.871 | -0.019 | 0.003 | 2.400E-10 | 40.179 |
| 219 | rs4908835 | C | T | 0.161 | 0.019 | 0.003 | 1.800E-12 | 49.780 |
| 220 | rs4931002 | A | C | 0.779 | -0.022 | 0.002 | 1.730E-19 | 81.664 |
| 221 | rs495149 | T | C | 0.164 | 0.024 | 0.003 | 1.150E-19 | 82.413 |
| 222 | rs496475 | G | T | 0.387 | 0.035 | 0.002 | 1.830E-66 | 296.675 |
| 223 | rs556063 | G | A | 0.574 | 0.013 | 0.002 | 1.680E-10 | 40.850 |
| 224 | rs55868524 | A | G | 0.606 | 0.013 | 0.002 | 9.911E-11 | 41.908 |
| 225 | rs55879743 | T | C | 0.066 | 0.078 | 0.004 | 7.070E-82 | 367.528 |
| 226 | rs55977204 | C | T | 0.113 | 0.020 | 0.003 | 3.600E-10 | 39.387 |
| 227 | rs56117721 | A | T | 0.072 | -0.104 | 0.004 | 6.637E-160 | 726.323 |
| 228 | rs56179563 | A | G | 0.389 | 0.018 | 0.002 | 3.870E-18 | 75.516 |
| 229 | rs56268488 | A | G | 0.086 | -0.021 | 0.004 | 4.140E-09 | 34.602 |
| 230 | rs56330463 | C | T | 0.553 | 0.039 | 0.002 | 5.380E-86 | 386.646 |
| 231 | rs574183 | G | A | 0.389 | -0.014 | 0.002 | 1.200E-11 | 46.028 |
| 232 | rs5747308 | C | A | 0.505 | 0.016 | 0.002 | 4.819E-15 | 61.441 |
| 233 | rs57633475 | G | A | 0.122 | -0.025 | 0.003 | 6.361E-16 | 65.404 |
| 234 | rs57834782 | A | T | 0.245 | -0.058 | 0.002 | 7.780E-141 | 638.842 |
| 235 | rs58745116 | A | G | 0.391 | -0.015 | 0.002 | 8.209E-14 | 55.852 |
| 236 | rs58833930 | T | C | 0.113 | -0.023 | 0.003 | 1.030E-13 | 55.395 |
| 237 | rs594479 | C | T | 0.701 | 0.012 | 0.002 | 1.200E-08 | 32.525 |
| 238 | rs60175411 | A | G | 0.167 | -0.030 | 0.003 | 1.250E-28 | 123.391 |
| 239 | rs60600003 | G | T | 0.100 | 0.043 | 0.003 | 3.050E-38 | 167.318 |
| 240 | rs6080761 | A | G | 0.419 | 0.018 | 0.002 | 4.750E-20 | 84.194 |
| 241 | rs6103572 | C | T | 0.729 | -0.024 | 0.002 | 5.959E-27 | 115.689 |
| 242 | rs6139104 | T | A | 0.093 | -0.019 | 0.003 | 1.780E-08 | 31.760 |
| 243 | rs6141755 | T | G | 0.236 | -0.017 | 0.002 | 8.300E-13 | 51.298 |
| 244 | rs61426394 | C | G | 0.067 | -0.025 | 0.004 | 6.990E-10 | 38.090 |
| 245 | rs61798836 | C | T | 0.170 | 0.015 | 0.003 | 3.750E-08 | 30.323 |
| 246 | rs62011287 | G | A | 0.344 | -0.013 | 0.002 | 1.320E-09 | 36.831 |
| 247 | rs62061733 | G | A | 0.229 | -0.032 | 0.002 | 1.920E-42 | 186.638 |
| 248 | rs62086903 | C | T | 0.232 | 0.030 | 0.002 | 2.270E-37 | 163.388 |
| 249 | rs62105489 | T | C | 0.053 | -0.030 | 0.004 | 1.130E-11 | 46.152 |
| 250 | rs62183994 | T | C | 0.051 | 0.027 | 0.005 | 2.650E-09 | 35.476 |
| 251 | rs62308111 | T | G | 0.221 | 0.015 | 0.002 | 1.500E-10 | 41.099 |
| 252 | rs62385501 | A | T | 0.308 | -0.014 | 0.002 | 1.680E-11 | 45.364 |
| 253 | rs62408224 | G | A | 0.350 | -0.042 | 0.002 | 5.690E-92 | 413.992 |
| 254 | rs62420764 | C | T | 0.140 | 0.019 | 0.003 | 4.210E-11 | 43.581 |
| 255 | rs62473720 | G | A | 0.317 | 0.014 | 0.002 | 2.640E-11 | 44.488 |
| 256 | rs62539154 | A | G | 0.097 | 0.027 | 0.003 | 7.199E-16 | 65.171 |
| 257 | rs634534 | G | A | 0.542 | 0.033 | 0.002 | 5.830E-61 | 271.424 |
| 258 | rs637064 | T | C | 0.556 | 0.024 | 0.002 | 5.430E-34 | 147.913 |
| 259 | rs6479336 | A | T | 0.185 | -0.029 | 0.003 | 3.440E-30 | 130.465 |
| 260 | rs6496717 | T | C | 0.760 | 0.019 | 0.002 | 4.420E-16 | 66.134 |
| 261 | rs6540985 | G | A | 0.319 | -0.020 | 0.002 | 6.720E-20 | 83.490 |
| 262 | rs6556313 | G | A | 0.332 | 0.019 | 0.002 | 2.160E-20 | 85.769 |
| 263 | rs6573020 | T | C | 0.433 | 0.021 | 0.002 | 2.310E-24 | 103.840 |
| 264 | rs6684992 | T | A | 0.119 | 0.038 | 0.003 | 6.060E-35 | 152.253 |
| 265 | rs6691839 | T | G | 0.765 | -0.013 | 0.002 | 1.450E-08 | 32.168 |
| 266 | rs6731125 | C | T | 0.565 | 0.017 | 0.002 | 1.040E-17 | 73.520 |
| 267 | rs6750754 | G | T | 0.264 | -0.064 | 0.002 | 8.356E-179 | 813.556 |
| 268 | rs67856193 | G | C | 0.308 | 0.024 | 0.002 | 1.630E-27 | 118.284 |
| 269 | rs6787336 | A | G | 0.287 | 0.036 | 0.002 | 3.290E-59 | 263.590 |
| 270 | rs6904506 | C | T | 0.088 | -0.052 | 0.004 | 2.250E-49 | 218.445 |
| 271 | rs6924350 | C | A | 0.184 | 0.050 | 0.003 | 1.760E-86 | 388.774 |
| 272 | rs6924387 | G | A | 0.410 | 0.017 | 0.002 | 2.190E-16 | 67.544 |
| 273 | rs6930635 | C | T | 0.078 | -0.038 | 0.004 | 6.830E-24 | 101.729 |
| 274 | rs6956283 | T | C | 0.733 | -0.016 | 0.002 | 2.480E-12 | 49.131 |
| 275 | rs6971710 | A | G | 0.201 | 0.028 | 0.002 | 2.750E-30 | 130.975 |
| 276 | rs6979947 | G | A | 0.266 | 0.013 | 0.002 | 3.750E-09 | 34.790 |
| 277 | rs6986109 | T | G | 0.704 | -0.016 | 0.002 | 4.240E-14 | 57.137 |
| 278 | rs6989099 | C | T | 0.317 | -0.018 | 0.002 | 8.580E-17 | 69.350 |
| 279 | rs699664 | T | C | 0.330 | -0.016 | 0.002 | 1.000E-13 | 55.468 |
| 280 | rs6999452 | A | G | 0.518 | 0.012 | 0.002 | 5.330E-09 | 34.132 |
| 281 | rs7026022 | C | A | 0.378 | 0.012 | 0.002 | 3.380E-09 | 35.003 |
| 282 | rs708776 | T | G | 0.941 | 0.027 | 0.004 | 2.910E-10 | 39.796 |
| 283 | rs7123726 | C | T | 0.210 | 0.017 | 0.002 | 3.950E-12 | 48.210 |
| 284 | rs7141943 | G | A | 0.395 | 0.016 | 0.002 | 7.000E-15 | 60.707 |
| 285 | rs71429414 | A | G | 0.196 | -0.019 | 0.002 | 1.030E-13 | 55.382 |
| 286 | rs7158239 | A | G | 0.395 | 0.016 | 0.002 | 2.280E-14 | 58.349 |
| 287 | rs71628184 | T | C | 0.094 | 0.021 | 0.003 | 8.320E-10 | 37.736 |
| 288 | rs7173571 | C | T | 0.527 | 0.014 | 0.002 | 2.400E-12 | 49.197 |
| 289 | rs7215391 | T | C | 0.257 | -0.013 | 0.002 | 2.490E-08 | 31.108 |
| 290 | rs725613 | G | T | 0.356 | -0.047 | 0.002 | 4.775E-116 | 524.852 |
| 291 | rs7257 | A | G | 0.567 | 0.034 | 0.002 | 3.600E-63 | 281.725 |
| 292 | rs72766638 | A | C | 0.164 | -0.024 | 0.003 | 9.940E-20 | 82.719 |
| 293 | rs72844043 | A | G | 0.432 | -0.011 | 0.002 | 1.540E-08 | 32.051 |
| 294 | rs72987040 | A | C | 0.126 | -0.022 | 0.003 | 4.070E-13 | 52.694 |
| 295 | rs72998585 | T | A | 0.134 | -0.091 | 0.003 | 1.000E-200 | 965.776 |
| 296 | rs73049239 | A | G | 0.059 | -0.029 | 0.004 | 1.160E-11 | 46.125 |
| 297 | rs73118830 | C | T | 0.086 | -0.029 | 0.004 | 1.080E-16 | 68.902 |
| 298 | rs73176183 | C | T | 0.126 | -0.020 | 0.003 | 6.109E-11 | 42.848 |
| 299 | rs73176685 | G | C | 0.243 | 0.024 | 0.002 | 1.070E-24 | 105.387 |
| 300 | rs73187852 | T | C | 0.275 | 0.018 | 0.002 | 1.570E-16 | 68.166 |
| 301 | rs73203442 | T | C | 0.122 | -0.096 | 0.003 | 1.000E-200 | 995.303 |
| 302 | rs73232881 | C | T | 0.213 | 0.067 | 0.002 | 8.222E-171 | 776.695 |
| 303 | rs73238201 | T | C | 0.179 | -0.016 | 0.003 | 1.520E-10 | 41.053 |
| 304 | rs73272842 | A | G | 0.123 | -0.025 | 0.003 | 4.839E-17 | 70.529 |
| 305 | rs7327960 | C | T | 0.827 | 0.018 | 0.003 | 1.910E-11 | 45.118 |
| 306 | rs73322872 | T | C | 0.242 | 0.015 | 0.002 | 3.910E-10 | 39.198 |
| 307 | rs73428834 | T | C | 0.075 | 0.030 | 0.004 | 2.720E-15 | 62.545 |
| 308 | rs7354779 | C | T | 0.271 | 0.018 | 0.002 | 2.710E-15 | 62.562 |
| 309 | rs7382061 | C | T | 0.591 | -0.055 | 0.002 | 1.130E-161 | 734.396 |
| 310 | rs73963711 | T | C | 0.211 | 0.022 | 0.002 | 3.280E-19 | 80.358 |
| 311 | rs7423615 | T | C | 0.187 | 0.023 | 0.003 | 5.100E-20 | 84.031 |
| 312 | rs74299961 | T | C | 0.203 | 0.013 | 0.002 | 5.000E-08 | 29.763 |
| 313 | rs743002 | C | T | 0.066 | -0.059 | 0.004 | 2.900E-48 | 213.297 |
| 314 | rs74331768 | A | G | 0.098 | 0.019 | 0.003 | 7.490E-09 | 33.463 |
| 315 | rs7441808 | G | A | 0.301 | 0.014 | 0.002 | 1.220E-10 | 41.474 |
| 316 | rs746550 | T | C | 0.236 | 0.023 | 0.002 | 9.030E-23 | 96.579 |
| 317 | rs7569084 | T | C | 0.584 | 0.019 | 0.002 | 1.190E-20 | 86.965 |
| 318 | rs7636495 | A | G | 0.181 | -0.024 | 0.003 | 1.840E-20 | 86.093 |
| 319 | rs7646283 | T | C | 0.368 | 0.026 | 0.002 | 2.330E-36 | 158.786 |
| 320 | rs7646695 | T | C | 0.180 | 0.018 | 0.003 | 1.820E-11 | 45.213 |
| 321 | rs76474320 | C | T | 0.077 | 0.028 | 0.004 | 5.760E-14 | 56.544 |
| 322 | rs7649812 | G | C | 0.243 | 0.016 | 0.002 | 3.740E-12 | 48.328 |
| 323 | rs76793172 | T | C | 0.092 | -0.042 | 0.003 | 8.070E-34 | 147.117 |
| 324 | rs76830965 | A | C | 0.118 | -0.023 | 0.003 | 3.270E-14 | 57.642 |
| 325 | rs7687708 | G | T | 0.220 | -0.015 | 0.002 | 1.560E-09 | 36.514 |
| 326 | rs76981581 | G | C | 0.048 | -0.026 | 0.005 | 2.220E-08 | 31.344 |
| 327 | rs7700687 | T | C | 0.617 | 0.040 | 0.002 | 4.819E-86 | 386.802 |
| 328 | rs778756 | G | A | 0.580 | -0.016 | 0.002 | 1.570E-15 | 63.609 |
| 329 | rs7797428 | T | C | 0.469 | 0.012 | 0.002 | 5.850E-10 | 38.429 |
| 330 | rs783646 | C | G | 0.817 | -0.019 | 0.003 | 2.410E-13 | 53.701 |
| 331 | rs7839946 | C | T | 0.057 | 0.024 | 0.004 | 2.000E-08 | 31.541 |
| 332 | rs7840212 | T | C | 0.337 | -0.040 | 0.002 | 7.110E-81 | 363.054 |
| 333 | rs7846314 | T | A | 0.187 | -0.033 | 0.003 | 3.920E-38 | 166.879 |
| 334 | rs7868130 | T | C | 0.259 | 0.032 | 0.002 | 3.350E-46 | 203.775 |
| 335 | rs7897422 | C | T | 0.192 | 0.021 | 0.003 | 1.850E-16 | 67.864 |
| 336 | rs79716587 | A | G | 0.127 | -0.024 | 0.003 | 3.990E-16 | 66.317 |
| 337 | rs7975 | A | G | 0.324 | -0.012 | 0.002 | 1.170E-08 | 32.577 |
| 338 | rs7986796 | T | G | 0.628 | 0.024 | 0.002 | 4.910E-32 | 138.994 |
| 339 | rs79881201 | T | C | 0.360 | 0.040 | 0.002 | 4.720E-85 | 382.075 |
| 340 | rs8 | T | C | 0.204 | 0.022 | 0.003 | 7.630E-18 | 74.176 |
| 341 | rs80066203 | T | C | 0.068 | -0.024 | 0.004 | 8.530E-10 | 37.689 |
| 342 | rs8012643 | T | C | 0.286 | 0.015 | 0.002 | 1.910E-12 | 49.659 |
| 343 | rs8020739 | T | G | 0.644 | 0.025 | 0.002 | 2.570E-32 | 140.184 |
| 344 | rs8026803 | C | T | 0.261 | -0.031 | 0.002 | 1.670E-43 | 191.440 |
| 345 | rs8044920 | T | C | 0.376 | 0.012 | 0.002 | 3.590E-09 | 34.884 |
| 346 | rs8061729 | C | T | 0.274 | -0.013 | 0.002 | 9.320E-09 | 33.012 |
| 347 | rs8076052 | C | A | 0.296 | -0.018 | 0.002 | 4.330E-17 | 70.737 |
| 348 | rs8108623 | A | C | 0.625 | 0.018 | 0.002 | 1.910E-16 | 67.794 |
| 349 | rs8142080 | T | G | 0.102 | -0.024 | 0.003 | 5.720E-13 | 52.010 |
| 350 | rs884634 | C | T | 0.415 | 0.012 | 0.002 | 6.830E-10 | 38.118 |
| 351 | rs911603 | A | C | 0.404 | -0.025 | 0.002 | 1.420E-33 | 145.944 |
| 352 | rs925966 | G | C | 0.759 | -0.018 | 0.002 | 1.320E-14 | 59.438 |
| 353 | rs9349322 | C | A | 0.147 | 0.022 | 0.003 | 4.040E-15 | 61.765 |
| 354 | rs9389268 | G | A | 0.256 | -0.045 | 0.002 | 3.690E-87 | 391.786 |
| 355 | rs9392525 | C | T | 0.576 | 0.013 | 0.002 | 6.950E-11 | 42.597 |
| 356 | rs941616 | T | C | 0.610 | -0.012 | 0.002 | 6.690E-09 | 33.662 |
| 357 | rs954954 | C | A | 0.105 | -0.035 | 0.003 | 3.570E-27 | 116.719 |
| 358 | rs962993 | T | C | 0.422 | -0.040 | 0.002 | 4.920E-90 | 405.072 |
| 359 | rs964184 | C | G | 0.867 | 0.029 | 0.003 | 4.390E-23 | 98.032 |
| 360 | rs9666598 | G | C | 0.843 | -0.020 | 0.003 | 1.360E-12 | 50.319 |
| 361 | rs9675999 | A | G | 0.627 | 0.016 | 0.002 | 1.590E-15 | 63.588 |
| 362 | rs9815874 | T | C | 0.300 | 0.028 | 0.002 | 1.930E-39 | 172.854 |
| 363 | rs9818987 | T | C | 0.306 | 0.014 | 0.002 | 2.600E-10 | 40.023 |
| 364 | rs9835307 | C | T | 0.658 | 0.026 | 0.002 | 2.690E-33 | 144.681 |
| 365 | rs9837045 | A | G | 0.455 | 0.011 | 0.002 | 1.410E-08 | 32.233 |
| 366 | rs9840310 | G | A | 0.735 | -0.013 | 0.002 | 1.300E-08 | 32.387 |
| 367 | rs9880192 | C | G | 0.411 | 0.042 | 0.002 | 9.080E-96 | 431.596 |
| 368 | rs9889262 | A | T | 0.364 | 0.028 | 0.002 | 4.180E-43 | 189.564 |
| 369 | rs9894839 | C | T | 0.635 | 0.012 | 0.002 | 1.330E-08 | 32.356 |
| 370 | rs9939774 | T | C | 0.405 | -0.029 | 0.002 | 1.980E-47 | 209.475 |
| 371 | rs9979383 | T | C | 0.630 | 0.028 | 0.002 | 8.000E-43 | 188.394 |

SNPs: single-nucleotide polymorphisms; eaf: effect allele frequency; se: standard error

Supplementary Table 4: Characteristics of SNPs used as instrumental variables for IgG levels (IgG-Asthma).

|  | SNPs | effect_allele | other_allele | eaf | beta | se | p | F-statistic |
| --- | --- | --- | --- | --- | --- | --- | --- | --- |
| 1 | rs115032978 | T | G | 0.050 | -0.036 | 0.008 | 9.526E-06 | 19.770 |
| 2 | rs11642879 | T | C | 0.771 | 0.019 | 0.004 | 9.962E-06 | 19.683 |
| 3 | rs12463441 | A | G | 0.577 | 0.016 | 0.004 | 9.877E-06 | 19.700 |
| 4 | rs1983814 | C | T | 0.831 | 0.021 | 0.005 | 5.574E-06 | 20.815 |
| 5 | rs2257609 | G | A | 0.542 | -0.016 | 0.004 | 8.432E-06 | 20.008 |
| 6 | rs2780076 | A | C | 0.638 | 0.016 | 0.004 | 6.477E-06 | 20.522 |
| 7 | rs34740816 | A | G | 0.154 | 0.021 | 0.005 | 7.397E-06 | 20.263 |
| 8 | rs35755728 | G | C | 0.118 | -0.024 | 0.005 | 7.944E-06 | 20.124 |
| 9 | rs4655368 | A | G | 0.272 | 0.018 | 0.004 | 4.135E-06 | 21.399 |
| 10 | rs58371504 | C | T | 0.050 | -0.037 | 0.008 | 2.796E-06 | 22.164 |
| 11 | rs7256714 | C | G | 0.242 | -0.018 | 0.004 | 7.885E-06 | 20.139 |
| 12 | rs75832668 | A | G | 0.090 | 0.028 | 0.006 | 5.145E-06 | 20.971 |
| 13 | rs9520848 | C | T | 0.222 | 0.018 | 0.004 | 8.777E-06 | 19.930 |

SNPs: single-nucleotide polymorphisms; eaf: effect allele frequency; se: standard error

## Supplementary Table 5: Characteristics of SNPs used as instrumental variables for eosinophil cell count (EOS-Asthma).

|  | SNPs | effect_allele | other_allele | eaf | beta | se | p | F-Statistic |
| --- | --- | --- | --- | --- | --- | --- | --- | --- |
| 1 | rs1004870 | T | C | 0.589 | 0.026 | 0.002 | 1.270E-38 | 169.025 |
| 2 | rs10059018 | T | G | 0.201 | -0.023 | 0.002 | 3.380E-21 | 89.428 |
| 3 | rs10062687 | G | T | 0.233 | 0.024 | 0.002 | 9.471E-25 | 105.654 |
| 4 | rs10100356 | A | G | 0.227 | -0.013 | 0.002 | 1.910E-08 | 31.618 |
| 5 | rs10165678 | A | G | 0.758 | -0.015 | 0.002 | 1.900E-10 | 40.639 |
| 6 | rs10174238 | A | G | 0.766 | 0.013 | 0.002 | 3.410E-08 | 30.498 |
| 7 | rs10195713 | T | C | 0.864 | 0.025 | 0.003 | 2.740E-17 | 71.619 |
| 8 | rs1036332 | C | A | 0.737 | -0.033 | 0.002 | 1.610E-47 | 209.857 |
| 9 | rs1037674 | T | G | 0.292 | 0.013 | 0.002 | 1.630E-09 | 36.418 |
| 10 | rs1039341 | T | C | 0.311 | -0.024 | 0.002 | 8.790E-29 | 124.044 |
| 11 | rs10472984 | G | C | 0.340 | -0.035 | 0.002 | 7.621E-63 | 280.201 |
| 12 | rs1047891 | A | C | 0.315 | 0.017 | 0.002 | 1.550E-15 | 63.632 |
| 13 | rs1057258 | T | C | 0.178 | -0.032 | 0.003 | 2.520E-35 | 153.974 |
| 14 | rs1059091 | G | A | 0.321 | 0.034 | 0.002 | 3.080E-56 | 249.834 |
| 15 | rs10745763 | T | G | 0.423 | 0.013 | 0.002 | 2.410E-11 | 44.687 |
| 16 | rs10777378 | A | G | 0.536 | -0.022 | 0.002 | 1.280E-29 | 127.829 |
| 17 | rs10782957 | T | C | 0.622 | 0.023 | 0.002 | 2.810E-28 | 121.820 |
| 18 | rs10876550 | A | G | 0.559 | 0.014 | 0.002 | 4.920E-12 | 47.781 |
| 19 | rs10900595 | A | C | 0.713 | 0.015 | 0.002 | 1.590E-12 | 50.022 |
| 20 | rs10930337 | T | C | 0.285 | 0.015 | 0.002 | 2.030E-11 | 45.034 |
| 21 | rs1099448 | T | C | 0.466 | 0.018 | 0.002 | 8.320E-19 | 78.497 |
| 22 | rs10995240 | C | G | 0.368 | -0.044 | 0.002 | 1.549E-101 | 457.737 |
| 23 | rs11065822 | T | G | 0.354 | 0.065 | 0.002 | 1.000E-200 | 925.739 |
| 24 | rs11071528 | C | G | 0.807 | 0.014 | 0.003 | 1.340E-08 | 32.304 |
| 25 | rs11079340 | C | T | 0.322 | 0.012 | 0.002 | 7.490E-09 | 33.472 |
| 26 | rs11088236 | T | C | 0.453 | 0.018 | 0.002 | 5.170E-20 | 84.037 |
| 27 | rs111759324 | T | C | 0.111 | -0.043 | 0.003 | 3.560E-42 | 185.373 |
| 28 | rs112036266 | T | C | 0.176 | 0.017 | 0.003 | 1.480E-10 | 41.134 |
| 29 | rs11204682 | T | G | 0.223 | -0.041 | 0.002 | 4.890E-67 | 299.558 |
| 30 | rs11228990 | T | A | 0.045 | 0.029 | 0.005 | 3.560E-09 | 34.899 |
| 31 | rs11236813 | C | G | 0.102 | -0.030 | 0.003 | 4.780E-20 | 84.205 |
| 32 | rs11255507 | G | T | 0.178 | 0.020 | 0.003 | 1.050E-14 | 59.875 |
| 33 | rs113473633 | G | A | 0.026 | -0.063 | 0.007 | 3.470E-22 | 93.952 |
| 34 | rs113496608 | A | G | 0.030 | -0.045 | 0.006 | 1.130E-14 | 59.746 |
| 35 | rs113542380 | A | G | 0.075 | -0.027 | 0.004 | 1.550E-12 | 50.049 |
| 36 | rs113859409 | A | G | 0.040 | -0.029 | 0.005 | 4.750E-08 | 29.862 |
| 37 | rs114152720 | A | G | 0.030 | -0.036 | 0.006 | 8.080E-10 | 37.792 |
| 38 | rs114741563 | C | T | 0.008 | 0.120 | 0.012 | 2.940E-25 | 107.960 |
| 39 | rs11555542 | C | T | 0.063 | 0.064 | 0.004 | 2.120E-54 | 241.503 |
| 40 | rs115647629 | A | G | 0.026 | -0.067 | 0.007 | 6.690E-25 | 106.329 |
| 41 | rs11571404 | T | C | 0.203 | 0.015 | 0.002 | 5.570E-10 | 38.525 |
| 42 | rs11578794 | T | A | 0.728 | -0.014 | 0.002 | 1.530E-09 | 36.549 |
| 43 | rs11628569 | G | A | 0.290 | -0.017 | 0.002 | 4.700E-14 | 56.955 |
| 44 | rs11647946 | A | G | 0.708 | -0.013 | 0.002 | 1.200E-09 | 37.034 |
| 45 | rs11684770 | G | A | 0.270 | -0.015 | 0.002 | 3.100E-11 | 44.173 |
| 46 | rs116888884 | A | G | 0.010 | -0.058 | 0.010 | 1.540E-08 | 32.052 |
| 47 | rs11695281 | T | C | 0.514 | -0.023 | 0.002 | 4.090E-31 | 134.665 |
| 48 | rs11701475 | C | T | 0.223 | 0.015 | 0.002 | 1.060E-09 | 37.264 |
| 49 | rs11702918 | T | C | 0.103 | -0.022 | 0.003 | 1.060E-11 | 46.290 |
| 50 | rs1170439 | C | T | 0.779 | 0.023 | 0.002 | 7.860E-21 | 87.757 |
| 51 | rs117068593 | T | C | 0.190 | -0.026 | 0.003 | 1.170E-24 | 105.266 |
| 52 | rs117481629 | C | T | 0.028 | 0.036 | 0.006 | 8.250E-09 | 33.263 |
| 53 | rs1178016 | T | C | 0.495 | 0.011 | 0.002 | 1.170E-08 | 32.582 |
| 54 | rs11786536 | A | G | 0.165 | -0.027 | 0.003 | 5.110E-23 | 97.765 |
| 55 | rs117955557 | T | G | 0.256 | -0.030 | 0.002 | 1.440E-40 | 178.070 |
| 56 | rs117961539 | A | G | 0.040 | -0.031 | 0.005 | 2.630E-09 | 35.494 |
| 57 | rs118013485 | A | G | 0.067 | -0.034 | 0.004 | 1.540E-16 | 68.229 |
| 58 | rs11869228 | T | C | 0.841 | 0.021 | 0.003 | 3.450E-14 | 57.557 |
| 59 | rs11886928 | G | T | 0.308 | -0.014 | 0.002 | 1.860E-11 | 45.197 |
| 60 | rs11931711 | T | C | 0.282 | 0.019 | 0.002 | 3.480E-18 | 75.689 |
| 61 | rs12100034 | A | G | 0.359 | -0.017 | 0.002 | 2.600E-16 | 67.218 |
| 62 | rs12154498 | C | A | 0.854 | -0.033 | 0.003 | 7.761E-31 | 133.448 |
| 63 | rs121564 | T | G | 0.270 | -0.017 | 0.002 | 1.260E-13 | 55.024 |
| 64 | rs12208103 | T | C | 0.378 | -0.031 | 0.002 | 6.830E-50 | 220.685 |
| 65 | rs12361586 | G | A | 0.146 | -0.015 | 0.003 | 2.480E-08 | 31.137 |
| 66 | rs12408934 | A | G | 0.103 | -0.041 | 0.003 | 2.360E-36 | 158.690 |
| 67 | rs12470046 | C | T | 0.319 | -0.012 | 0.002 | 6.930E-09 | 33.613 |
| 68 | rs12487980 | A | C | 0.634 | 0.013 | 0.002 | 1.500E-09 | 36.587 |
| 69 | rs12530946 | G | A | 0.613 | 0.043 | 0.002 | 3.990E-97 | 437.532 |
| 70 | rs12540285 | G | A | 0.221 | 0.015 | 0.002 | 1.010E-09 | 37.365 |
| 71 | rs12545733 | C | T | 0.703 | -0.031 | 0.002 | 8.900E-45 | 197.294 |
| 72 | rs1257192 | G | A | 0.812 | 0.015 | 0.003 | 8.810E-09 | 33.147 |
| 73 | rs12581511 | G | C | 0.173 | -0.018 | 0.003 | 2.070E-11 | 44.968 |
| 74 | rs12705849 | A | G | 0.407 | -0.021 | 0.002 | 2.550E-24 | 103.664 |
| 75 | rs12820863 | T | C | 0.352 | 0.022 | 0.002 | 1.070E-25 | 110.005 |
| 76 | rs12861824 | C | T | 0.275 | -0.021 | 0.002 | 2.590E-18 | 76.267 |
| 77 | rs12878610 | C | T | 0.476 | -0.013 | 0.002 | 3.950E-11 | 43.710 |
| 78 | rs12882281 | C | T | 0.239 | -0.014 | 0.002 | 7.370E-09 | 33.492 |
| 79 | rs12928503 | T | C | 0.020 | -0.049 | 0.007 | 1.920E-11 | 45.120 |
| 80 | rs12941068 | A | G | 0.286 | -0.014 | 0.002 | 9.590E-10 | 37.456 |
| 81 | rs12978850 | T | C | 0.194 | -0.014 | 0.003 | 3.990E-08 | 30.206 |
| 82 | rs13073683 | C | T | 0.399 | 0.016 | 0.002 | 1.320E-14 | 59.422 |
| 83 | rs13105682 | G | T | 0.060 | -0.027 | 0.004 | 8.220E-10 | 37.770 |
| 84 | rs13138355 | T | C | 0.186 | -0.055 | 0.003 | 2.350E-102 | 461.852 |
| 85 | rs13139941 | G | A | 0.803 | -0.014 | 0.002 | 2.840E-08 | 30.869 |
| 86 | rs13207791 | G | A | 0.089 | 0.026 | 0.004 | 4.300E-13 | 52.567 |
| 87 | rs13226583 | T | A | 0.116 | -0.062 | 0.003 | 1.590E-88 | 398.258 |
| 88 | rs1323650 | T | G | 0.681 | -0.014 | 0.002 | 2.530E-10 | 40.058 |
| 89 | rs13251643 | T | A | 0.044 | 0.030 | 0.005 | 3.540E-10 | 39.417 |
| 90 | rs13313564 | A | G | 0.035 | -0.069 | 0.005 | 4.210E-37 | 162.156 |
| 91 | rs13511 | C | T | 0.577 | -0.016 | 0.002 | 4.130E-16 | 66.273 |
| 92 | rs1353286 | G | T | 0.453 | 0.020 | 0.002 | 3.090E-24 | 103.303 |
| 93 | rs1365623 | T | C | 0.372 | 0.012 | 0.002 | 1.710E-08 | 31.857 |
| 94 | rs137906075 | G | A | 0.046 | 0.043 | 0.005 | 1.390E-18 | 77.526 |
| 95 | rs1395269 | G | T | 0.301 | -0.024 | 0.002 | 1.060E-27 | 119.155 |
| 96 | rs139640694 | G | A | 0.093 | 0.025 | 0.003 | 3.540E-13 | 52.967 |
| 97 | rs1406449 | C | T | 0.462 | -0.012 | 0.002 | 8.690E-10 | 37.662 |
| 98 | rs1414517 | G | C | 0.187 | -0.025 | 0.003 | 1.360E-22 | 95.789 |
| 99 | rs1427499 | G | A | 0.710 | 0.019 | 0.002 | 1.300E-17 | 73.113 |
| 100 | rs143491704 | G | C | 0.036 | -0.031 | 0.006 | 1.470E-08 | 32.146 |
| 101 | rs144569746 | T | C | 0.102 | 0.059 | 0.003 | 4.230E-72 | 322.760 |
| 102 | rs1448187 | C | T | 0.700 | 0.016 | 0.002 | 7.890E-12 | 46.877 |
| 103 | rs145947882 | C | A | 0.026 | -0.060 | 0.006 | 2.980E-21 | 89.673 |
| 104 | rs146078144 | A | G | 0.010 | 0.086 | 0.010 | 2.000E-16 | 67.705 |
| 105 | rs146730870 | A | G | 0.010 | -0.087 | 0.010 | 4.950E-17 | 70.456 |
| 106 | rs14713 | A | G | 0.205 | -0.014 | 0.002 | 3.530E-08 | 30.438 |
| 107 | rs1471816 | A | G | 0.503 | 0.011 | 0.002 | 3.940E-08 | 30.214 |
| 108 | rs1479918 | T | A | 0.260 | -0.019 | 0.002 | 1.050E-16 | 68.961 |
| 109 | rs148219449 | A | G | 0.014 | 0.064 | 0.010 | 6.020E-11 | 42.881 |
| 110 | rs149110519 | T | C | 0.036 | 0.038 | 0.005 | 1.390E-12 | 50.279 |
| 111 | rs150640087 | T | G | 0.016 | 0.100 | 0.008 | 1.800E-35 | 154.670 |
| 112 | rs1516527 | C | T | 0.951 | -0.032 | 0.005 | 2.250E-12 | 49.317 |
| 113 | rs1519602 | G | T | 0.648 | 0.013 | 0.002 | 7.430E-10 | 37.951 |
| 114 | rs1529745 | G | C | 0.175 | 0.016 | 0.003 | 2.900E-09 | 35.302 |
| 115 | rs1539174 | G | C | 0.237 | 0.038 | 0.002 | 1.120E-59 | 265.764 |
| 116 | rs1547258 | C | T | 0.708 | -0.019 | 0.002 | 1.490E-18 | 77.408 |
| 117 | rs159963 | A | C | 0.582 | 0.019 | 0.002 | 9.881E-22 | 91.844 |
| 118 | rs1672753 | T | C | 0.811 | -0.017 | 0.003 | 6.741E-12 | 47.174 |
| 119 | rs1684578 | T | G | 0.416 | 0.016 | 0.002 | 5.150E-16 | 65.805 |
| 120 | rs1689510 | C | G | 0.338 | 0.027 | 0.002 | 1.060E-36 | 160.287 |
| 121 | rs16903574 | G | C | 0.078 | 0.029 | 0.004 | 1.170E-13 | 55.149 |
| 122 | rs16956811 | G | T | 0.081 | -0.029 | 0.004 | 1.750E-15 | 63.428 |
| 123 | rs17061503 | A | G | 0.307 | 0.036 | 0.002 | 8.041E-61 | 270.791 |
| 124 | rs17175830 | A | G | 0.238 | 0.032 | 0.002 | 4.130E-42 | 185.132 |
| 125 | rs174548 | G | C | 0.314 | -0.023 | 0.002 | 4.080E-27 | 116.421 |
| 126 | rs17482472 | A | G | 0.099 | -0.031 | 0.003 | 5.339E-21 | 88.520 |
| 127 | rs17516457 | C | T | 0.417 | -0.064 | 0.002 | 1.000E-200 | 1012.110 |
| 128 | rs175705 | G | C | 0.719 | 0.038 | 0.002 | 8.800E-67 | 298.275 |
| 129 | rs17653687 | G | A | 0.179 | -0.018 | 0.003 | 4.060E-12 | 48.165 |
| 130 | rs17668272 | T | G | 0.118 | -0.042 | 0.003 | 7.480E-42 | 183.870 |
| 131 | rs17682575 | T | C | 0.201 | -0.018 | 0.002 | 1.330E-12 | 50.349 |
| 132 | rs17689159 | C | T | 0.315 | -0.014 | 0.002 | 1.400E-11 | 45.740 |
| 133 | rs17758695 | T | C | 0.030 | -0.122 | 0.006 | 1.320E-93 | 421.552 |
| 134 | rs17849501 | T | C | 0.052 | -0.027 | 0.004 | 2.100E-09 | 35.933 |
| 135 | rs1800692 | G | A | 0.588 | -0.018 | 0.002 | 8.921E-19 | 78.373 |
| 136 | rs180506 | A | G | 0.776 | -0.023 | 0.002 | 5.820E-22 | 92.918 |
| 137 | rs1828803 | A | C | 0.392 | 0.013 | 0.002 | 1.280E-10 | 41.388 |
| 138 | rs1861489 | A | T | 0.782 | 0.013 | 0.002 | 4.140E-08 | 30.136 |
| 139 | rs201798 | A | G | 0.615 | 0.020 | 0.002 | 5.710E-23 | 97.510 |
| 140 | rs2025489 | G | A | 0.526 | 0.016 | 0.002 | 2.420E-15 | 62.791 |
| 141 | rs2089979 | G | A | 0.416 | -0.015 | 0.002 | 1.250E-13 | 55.016 |
| 142 | rs2182885 | A | G | 0.605 | 0.026 | 0.002 | 2.050E-38 | 168.181 |
| 143 | rs2223043 | G | A | 0.307 | 0.019 | 0.002 | 3.230E-17 | 71.323 |
| 144 | rs2228467 | C | T | 0.062 | 0.062 | 0.004 | 8.831E-52 | 229.450 |
| 145 | rs2239633 | A | G | 0.484 | 0.035 | 0.002 | 1.500E-68 | 306.531 |
| 146 | rs2253427 | C | T | 0.778 | -0.014 | 0.002 | 8.680E-09 | 33.169 |
| 147 | rs2399441 | C | T | 0.351 | -0.019 | 0.002 | 8.080E-20 | 83.163 |
| 148 | rs2410732 | A | C | 0.760 | 0.014 | 0.002 | 5.540E-09 | 34.035 |
| 149 | rs2419313 | A | G | 0.143 | -0.021 | 0.003 | 5.210E-13 | 52.193 |
| 150 | rs2431097 | T | C | 0.486 | 0.015 | 0.002 | 5.950E-14 | 56.479 |
| 151 | rs2497318 | T | C | 0.443 | -0.030 | 0.002 | 1.960E-49 | 218.570 |
| 152 | rs2502995 | C | T | 0.570 | 0.022 | 0.002 | 1.060E-27 | 119.149 |
| 153 | rs2505521 | T | C | 0.829 | -0.015 | 0.003 | 1.450E-08 | 32.165 |
| 154 | rs2566133 | C | T | 0.584 | -0.014 | 0.002 | 1.060E-12 | 50.823 |
| 155 | rs2579505 | C | T | 0.638 | 0.042 | 0.002 | 5.790E-94 | 423.333 |
| 156 | rs2646438 | A | G | 0.566 | -0.021 | 0.002 | 1.200E-24 | 105.121 |
| 157 | rs2713548 | T | C | 0.633 | 0.013 | 0.002 | 1.020E-09 | 37.355 |
| 158 | rs2788211 | C | T | 0.817 | 0.016 | 0.003 | 4.790E-10 | 38.809 |
| 159 | rs2807740 | T | C | 0.770 | 0.035 | 0.002 | 1.050E-50 | 224.427 |
| 160 | rs2817377 | A | G | 0.537 | -0.012 | 0.002 | 2.920E-08 | 30.817 |
| 161 | rs28362902 | A | G | 0.133 | 0.017 | 0.003 | 1.660E-08 | 31.908 |
| 162 | rs2838317 | C | A | 0.550 | 0.013 | 0.002 | 1.200E-10 | 41.549 |
| 163 | rs28421324 | T | A | 0.102 | 0.036 | 0.003 | 1.570E-27 | 118.334 |
| 164 | rs2847266 | T | C | 0.717 | -0.014 | 0.002 | 1.220E-10 | 41.494 |
| 165 | rs2850542 | T | G | 0.556 | -0.013 | 0.002 | 6.500E-11 | 42.740 |
| 166 | rs28532037 | A | G | 0.907 | -0.030 | 0.003 | 4.190E-18 | 75.353 |
| 167 | rs2864936 | C | A | 0.805 | -0.014 | 0.003 | 1.710E-08 | 31.861 |
| 168 | rs2887502 | T | C | 0.590 | -0.014 | 0.002 | 1.740E-11 | 45.289 |
| 169 | rs2894401 | G | A | 0.705 | -0.021 | 0.002 | 6.160E-21 | 88.215 |
| 170 | rs290430 | T | C | 0.345 | -0.012 | 0.002 | 8.310E-09 | 33.253 |
| 171 | rs2920505 | A | G | 0.602 | -0.030 | 0.002 | 9.469E-49 | 215.575 |
| 172 | rs295 | C | A | 0.235 | 0.016 | 0.002 | 1.160E-11 | 46.108 |
| 173 | rs295273 | A | G | 0.256 | 0.021 | 0.002 | 1.500E-19 | 81.897 |
| 174 | rs2979489 | A | G | 0.742 | 0.014 | 0.002 | 1.330E-09 | 36.831 |
| 175 | rs2992333 | A | G | 0.599 | -0.034 | 0.002 | 1.350E-63 | 283.609 |
| 176 | rs301162 | G | A | 0.853 | 0.026 | 0.003 | 1.160E-20 | 86.962 |
| 177 | rs3024971 | G | T | 0.107 | -0.040 | 0.003 | 5.040E-36 | 157.182 |
| 178 | rs3093023 | A | G | 0.434 | 0.013 | 0.002 | 2.270E-10 | 40.257 |
| 179 | rs3096309 | T | C | 0.824 | 0.015 | 0.003 | 1.360E-08 | 32.304 |
| 180 | rs3110791 | C | T | 0.623 | 0.015 | 0.002 | 7.490E-14 | 56.006 |
| 181 | rs3218148 | A | G | 0.540 | -0.018 | 0.002 | 7.079E-19 | 78.836 |
| 182 | rs33982662 | A | C | 0.285 | 0.020 | 0.002 | 1.190E-20 | 86.909 |
| 183 | rs34173062 | A | G | 0.073 | 0.055 | 0.004 | 1.550E-40 | 177.857 |
| 184 | rs34210653 | A | G | 0.021 | -0.176 | 0.007 | 8.110E-140 | 633.920 |
| 185 | rs34212866 | G | A | 0.221 | 0.020 | 0.002 | 5.740E-17 | 70.187 |
| 186 | rs34363176 | G | C | 0.244 | 0.013 | 0.002 | 8.430E-09 | 33.238 |
| 187 | rs34439695 | T | C | 0.035 | -0.038 | 0.005 | 3.670E-12 | 48.365 |
| 188 | rs34448954 | T | C | 0.106 | -0.025 | 0.003 | 3.870E-15 | 61.851 |
| 189 | rs34466956 | T | C | 0.568 | -0.014 | 0.002 | 2.600E-11 | 44.532 |
| 190 | rs34495 | T | G | 0.304 | -0.020 | 0.002 | 3.670E-20 | 84.674 |
| 191 | rs34505104 | G | A | 0.306 | -0.026 | 0.002 | 1.220E-33 | 146.309 |
| 192 | rs34631302 | G | A | 0.645 | -0.012 | 0.002 | 9.660E-09 | 32.963 |
| 193 | rs350836 | T | C | 0.710 | -0.017 | 0.002 | 6.411E-15 | 60.854 |
| 194 | rs35249183 | G | A | 0.100 | 0.040 | 0.003 | 1.460E-32 | 141.322 |
| 195 | rs35409523 | A | G | 0.076 | 0.050 | 0.004 | 1.430E-40 | 178.080 |
| 196 | rs36084354 | A | G | 0.092 | -0.045 | 0.003 | 1.170E-39 | 173.824 |
| 197 | rs3731211 | A | T | 0.720 | 0.018 | 0.002 | 2.340E-15 | 62.855 |
| 198 | rs3742704 | C | A | 0.092 | 0.019 | 0.003 | 1.540E-08 | 32.048 |
| 199 | rs3746420 | C | G | 0.061 | -0.032 | 0.004 | 2.260E-14 | 58.368 |
| 200 | rs3747869 | C | A | 0.901 | 0.020 | 0.003 | 2.310E-09 | 35.743 |
| 201 | rs3757114 | C | A | 0.474 | -0.025 | 0.002 | 6.150E-37 | 161.367 |
| 202 | rs3785356 | T | C | 0.298 | 0.030 | 0.002 | 1.700E-43 | 191.504 |
| 203 | rs3786586 | G | A | 0.155 | 0.032 | 0.003 | 4.420E-31 | 134.593 |
| 204 | rs3790163 | G | A | 0.792 | -0.015 | 0.003 | 1.350E-09 | 36.794 |
| 205 | rs3804590 | T | G | 0.317 | 0.018 | 0.002 | 3.760E-17 | 70.988 |
| 206 | rs3812206 | T | C | 0.300 | -0.012 | 0.002 | 2.070E-08 | 31.485 |
| 207 | rs3823536 | A | G | 0.467 | -0.018 | 0.002 | 3.160E-19 | 80.493 |
| 208 | rs3824867 | G | A | 0.712 | 0.017 | 0.002 | 1.770E-14 | 58.868 |
| 209 | rs3846855 | A | G | 0.183 | 0.028 | 0.003 | 5.010E-28 | 120.631 |
| 210 | rs3850107 | G | C | 0.294 | -0.014 | 0.002 | 3.940E-10 | 39.210 |
| 211 | rs3950296 | G | C | 0.244 | 0.024 | 0.002 | 8.300E-25 | 105.900 |
| 212 | rs397187 | C | T | 0.430 | -0.012 | 0.002 | 1.280E-09 | 36.890 |
| 213 | rs4074672 | T | C | 0.369 | 0.015 | 0.002 | 1.480E-12 | 50.140 |
| 214 | rs410867 | G | A | 0.217 | -0.059 | 0.002 | 4.121E-134 | 607.773 |
| 215 | rs412884 | C | T | 0.672 | 0.058 | 0.002 | 2.218E-164 | 746.797 |
| 216 | rs41313381 | A | C | 0.031 | 0.050 | 0.006 | 7.050E-19 | 78.865 |
| 217 | rs4142528 | A | T | 0.672 | -0.075 | 0.002 | 1.000E-200 | 1247.717 |
| 218 | rs4148757 | C | A | 0.210 | -0.017 | 0.002 | 2.240E-12 | 49.345 |
| 219 | rs4149909 | G | A | 0.033 | -0.033 | 0.006 | 2.070E-09 | 35.970 |
| 220 | rs4236746 | G | A | 0.975 | 0.057 | 0.006 | 6.780E-19 | 78.940 |
| 221 | rs4240624 | A | G | 0.909 | -0.020 | 0.003 | 3.620E-09 | 34.872 |
| 222 | rs4280242 | T | C | 0.754 | -0.033 | 0.002 | 1.030E-45 | 201.607 |
| 223 | rs4310436 | A | G | 0.120 | 0.019 | 0.003 | 9.150E-10 | 37.563 |
| 224 | rs4347868 | A | G | 0.238 | 0.013 | 0.002 | 1.690E-08 | 31.873 |
| 225 | rs4409785 | C | T | 0.172 | 0.016 | 0.003 | 4.250E-10 | 39.050 |
| 226 | rs45577137 | G | A | 0.045 | -0.060 | 0.005 | 5.100E-31 | 134.275 |
| 227 | rs460631 | G | A | 0.885 | 0.026 | 0.003 | 1.880E-16 | 67.847 |
| 228 | rs4618204 | C | T | 0.444 | 0.018 | 0.002 | 1.090E-19 | 82.572 |
| 229 | rs4652560 | T | A | 0.618 | -0.015 | 0.002 | 5.230E-14 | 56.743 |
| 230 | rs4675190 | T | C | 0.591 | -0.012 | 0.002 | 7.570E-09 | 33.453 |
| 231 | rs4680250 | G | C | 0.693 | -0.016 | 0.002 | 4.850E-13 | 52.331 |
| 232 | rs4703589 | C | T | 0.533 | 0.014 | 0.002 | 3.260E-13 | 53.107 |
| 233 | rs4703730 | T | C | 0.517 | -0.016 | 0.002 | 6.910E-16 | 65.248 |
| 234 | rs4721559 | T | C | 0.278 | -0.017 | 0.002 | 6.560E-15 | 60.831 |
| 235 | rs473739 | G | T | 0.319 | -0.014 | 0.002 | 7.070E-11 | 42.556 |
| 236 | rs4746153 | C | G | 0.185 | 0.016 | 0.003 | 8.580E-10 | 37.669 |
| 237 | rs4849903 | T | C | 0.633 | 0.021 | 0.002 | 9.949E-25 | 105.526 |
| 238 | rs4870977 | C | G | 0.871 | -0.019 | 0.003 | 2.400E-10 | 40.179 |
| 239 | rs4908835 | C | T | 0.161 | 0.019 | 0.003 | 1.800E-12 | 49.780 |
| 240 | rs4931002 | A | C | 0.779 | -0.022 | 0.002 | 1.730E-19 | 81.664 |
| 241 | rs495149 | T | C | 0.164 | 0.024 | 0.003 | 1.150E-19 | 82.413 |
| 242 | rs496475 | G | T | 0.387 | 0.035 | 0.002 | 1.830E-66 | 296.675 |
| 243 | rs547211157 | C | G | 0.014 | 0.050 | 0.009 | 1.780E-08 | 31.766 |
| 244 | rs556063 | G | A | 0.574 | 0.013 | 0.002 | 1.680E-10 | 40.850 |
| 245 | rs55868524 | A | G | 0.606 | 0.013 | 0.002 | 9.911E-11 | 41.908 |
| 246 | rs55879743 | T | C | 0.066 | 0.078 | 0.004 | 7.070E-82 | 367.528 |
| 247 | rs55977204 | C | T | 0.113 | 0.020 | 0.003 | 3.600E-10 | 39.387 |
| 248 | rs56117721 | A | T | 0.072 | -0.104 | 0.004 | 6.637E-160 | 726.323 |
| 249 | rs56179563 | A | G | 0.389 | 0.018 | 0.002 | 3.870E-18 | 75.516 |
| 250 | rs56268488 | A | G | 0.086 | -0.021 | 0.004 | 4.140E-09 | 34.602 |
| 251 | rs56330463 | C | T | 0.553 | 0.039 | 0.002 | 5.380E-86 | 386.646 |
| 252 | rs574183 | G | A | 0.389 | -0.014 | 0.002 | 1.200E-11 | 46.028 |
| 253 | rs5747308 | C | A | 0.505 | 0.016 | 0.002 | 4.819E-15 | 61.441 |
| 254 | rs57633475 | G | A | 0.122 | -0.025 | 0.003 | 6.361E-16 | 65.404 |
| 255 | rs57834782 | A | T | 0.245 | -0.058 | 0.002 | 7.780E-141 | 638.842 |
| 256 | rs58745116 | A | G | 0.391 | -0.015 | 0.002 | 8.209E-14 | 55.852 |
| 257 | rs58833930 | T | C | 0.113 | -0.023 | 0.003 | 1.030E-13 | 55.395 |
| 258 | rs594479 | C | T | 0.701 | 0.012 | 0.002 | 1.200E-08 | 32.525 |
| 259 | rs60175411 | A | G | 0.167 | -0.030 | 0.003 | 1.250E-28 | 123.391 |
| 260 | rs60600003 | G | T | 0.100 | 0.043 | 0.003 | 3.050E-38 | 167.318 |
| 261 | rs6080761 | A | G | 0.419 | 0.018 | 0.002 | 4.750E-20 | 84.194 |
| 262 | rs6103572 | C | T | 0.729 | -0.024 | 0.002 | 5.959E-27 | 115.689 |
| 263 | rs6139104 | T | A | 0.093 | -0.019 | 0.003 | 1.780E-08 | 31.760 |
| 264 | rs6141755 | T | G | 0.236 | -0.017 | 0.002 | 8.300E-13 | 51.298 |
| 265 | rs61426394 | C | G | 0.067 | -0.025 | 0.004 | 6.990E-10 | 38.090 |
| 266 | rs61798836 | C | T | 0.170 | 0.015 | 0.003 | 3.750E-08 | 30.323 |
| 267 | rs62006172 | A | G | 0.042 | -0.038 | 0.005 | 4.620E-14 | 56.971 |
| 268 | rs62011287 | G | A | 0.344 | -0.013 | 0.002 | 1.320E-09 | 36.831 |
| 269 | rs62061733 | G | A | 0.229 | -0.032 | 0.002 | 1.920E-42 | 186.638 |
| 270 | rs62086903 | C | T | 0.232 | 0.030 | 0.002 | 2.270E-37 | 163.388 |
| 271 | rs62105489 | T | C | 0.053 | -0.030 | 0.004 | 1.130E-11 | 46.152 |
| 272 | rs62117160 | A | G | 0.045 | -0.048 | 0.005 | 2.150E-23 | 99.468 |
| 273 | rs62183994 | T | C | 0.051 | 0.027 | 0.005 | 2.650E-09 | 35.476 |
| 274 | rs62308111 | T | G | 0.221 | 0.015 | 0.002 | 1.500E-10 | 41.099 |
| 275 | rs62385501 | A | T | 0.308 | -0.014 | 0.002 | 1.680E-11 | 45.364 |
| 276 | rs62395833 | C | G | 0.043 | 0.037 | 0.005 | 5.270E-14 | 56.721 |
| 277 | rs62408224 | G | A | 0.350 | -0.042 | 0.002 | 5.690E-92 | 413.992 |
| 278 | rs62420764 | C | T | 0.140 | 0.019 | 0.003 | 4.210E-11 | 43.581 |
| 279 | rs62473720 | G | A | 0.317 | 0.014 | 0.002 | 2.640E-11 | 44.488 |
| 280 | rs634534 | G | A | 0.542 | 0.033 | 0.002 | 5.830E-61 | 271.424 |
| 281 | rs637064 | T | C | 0.556 | 0.024 | 0.002 | 5.430E-34 | 147.913 |
| 282 | rs6479336 | A | T | 0.185 | -0.029 | 0.003 | 3.440E-30 | 130.465 |
| 283 | rs6490291 | A | T | 0.964 | -0.066 | 0.006 | 1.110E-29 | 128.177 |
| 284 | rs6496717 | T | C | 0.760 | 0.019 | 0.002 | 4.420E-16 | 66.134 |
| 285 | rs6540985 | G | A | 0.319 | -0.020 | 0.002 | 6.720E-20 | 83.490 |
| 286 | rs6556313 | G | A | 0.332 | 0.019 | 0.002 | 2.160E-20 | 85.769 |
| 287 | rs6573020 | T | C | 0.433 | 0.021 | 0.002 | 2.310E-24 | 103.840 |
| 288 | rs668248 | C | G | 0.616 | -0.017 | 0.002 | 5.490E-16 | 65.685 |
| 289 | rs6684992 | T | A | 0.119 | 0.038 | 0.003 | 6.060E-35 | 152.253 |
| 290 | rs6691839 | T | G | 0.765 | -0.013 | 0.002 | 1.450E-08 | 32.168 |
| 291 | rs6731125 | C | T | 0.565 | 0.017 | 0.002 | 1.040E-17 | 73.520 |
| 292 | rs6750754 | G | T | 0.264 | -0.064 | 0.002 | 8.356E-179 | 813.556 |
| 293 | rs67856193 | G | C | 0.308 | 0.024 | 0.002 | 1.630E-27 | 118.284 |
| 294 | rs6787336 | A | G | 0.287 | 0.036 | 0.002 | 3.290E-59 | 263.590 |
| 295 | rs6904506 | C | T | 0.088 | -0.052 | 0.004 | 2.250E-49 | 218.445 |
| 296 | rs6924350 | C | A | 0.184 | 0.050 | 0.003 | 1.760E-86 | 388.774 |
| 297 | rs6924387 | G | A | 0.410 | 0.017 | 0.002 | 2.190E-16 | 67.544 |
| 298 | rs6956283 | T | C | 0.733 | -0.016 | 0.002 | 2.480E-12 | 49.131 |
| 299 | rs6971710 | A | G | 0.201 | 0.028 | 0.002 | 2.750E-30 | 130.975 |
| 300 | rs6979947 | G | A | 0.266 | 0.013 | 0.002 | 3.750E-09 | 34.790 |
| 301 | rs6986109 | T | G | 0.704 | -0.016 | 0.002 | 4.240E-14 | 57.137 |
| 302 | rs6989099 | C | T | 0.317 | -0.018 | 0.002 | 8.580E-17 | 69.350 |
| 303 | rs699664 | T | C | 0.330 | -0.016 | 0.002 | 1.000E-13 | 55.468 |
| 304 | rs6999452 | A | G | 0.518 | 0.012 | 0.002 | 5.330E-09 | 34.132 |
| 305 | rs7026022 | C | A | 0.378 | 0.012 | 0.002 | 3.380E-09 | 35.003 |
| 306 | rs7080536 | A | G | 0.043 | -0.044 | 0.005 | 1.170E-18 | 77.854 |
| 307 | rs708776 | T | G | 0.941 | 0.027 | 0.004 | 2.910E-10 | 39.796 |
| 308 | rs7141943 | G | A | 0.395 | 0.016 | 0.002 | 7.000E-15 | 60.707 |
| 309 | rs71429414 | A | G | 0.196 | -0.019 | 0.002 | 1.030E-13 | 55.382 |
| 310 | rs71508968 | A | G | 0.026 | 0.036 | 0.006 | 1.280E-08 | 32.417 |
| 311 | rs7158239 | A | G | 0.395 | 0.016 | 0.002 | 2.280E-14 | 58.349 |
| 312 | rs71628184 | T | C | 0.094 | 0.021 | 0.003 | 8.320E-10 | 37.736 |
| 313 | rs7215391 | T | C | 0.257 | -0.013 | 0.002 | 2.490E-08 | 31.108 |
| 314 | rs7220649 | T | C | 0.043 | -0.028 | 0.005 | 1.230E-08 | 32.495 |
| 315 | rs725613 | G | T | 0.356 | -0.047 | 0.002 | 4.775E-116 | 524.852 |
| 316 | rs7257 | A | G | 0.567 | 0.034 | 0.002 | 3.600E-63 | 281.725 |
| 317 | rs72766638 | A | C | 0.164 | -0.024 | 0.003 | 9.940E-20 | 82.719 |
| 318 | rs72834751 | T | C | 0.013 | -0.072 | 0.009 | 1.500E-14 | 59.192 |
| 319 | rs72844043 | A | G | 0.432 | -0.011 | 0.002 | 1.540E-08 | 32.051 |
| 320 | rs72987040 | A | C | 0.126 | -0.022 | 0.003 | 4.070E-13 | 52.694 |
| 321 | rs72998585 | T | A | 0.134 | -0.091 | 0.003 | 1.000E-200 | 965.776 |
| 322 | rs73049239 | A | G | 0.059 | -0.029 | 0.004 | 1.160E-11 | 46.125 |
| 323 | rs73072498 | C | T | 0.009 | 0.060 | 0.010 | 9.630E-09 | 32.966 |
| 324 | rs73118830 | C | T | 0.086 | -0.029 | 0.004 | 1.080E-16 | 68.902 |
| 325 | rs73176183 | C | T | 0.126 | -0.020 | 0.003 | 6.109E-11 | 42.848 |
| 326 | rs73176685 | G | C | 0.243 | 0.024 | 0.002 | 1.070E-24 | 105.387 |
| 327 | rs73187852 | T | C | 0.275 | 0.018 | 0.002 | 1.570E-16 | 68.166 |
| 328 | rs73202462 | A | G | 0.020 | 0.057 | 0.008 | 2.140E-13 | 53.949 |
| 329 | rs73203442 | T | C | 0.122 | -0.096 | 0.003 | 1.000E-200 | 995.303 |
| 330 | rs73232881 | C | T | 0.213 | 0.067 | 0.002 | 8.222E-171 | 776.695 |
| 331 | rs73238201 | T | C | 0.179 | -0.016 | 0.003 | 1.520E-10 | 41.053 |
| 332 | rs73272842 | A | G | 0.123 | -0.025 | 0.003 | 4.839E-17 | 70.529 |
| 333 | rs7327960 | C | T | 0.827 | 0.018 | 0.003 | 1.910E-11 | 45.118 |
| 334 | rs73322872 | T | C | 0.242 | 0.015 | 0.002 | 3.910E-10 | 39.198 |
| 335 | rs73428834 | T | C | 0.075 | 0.030 | 0.004 | 2.720E-15 | 62.545 |
| 336 | rs7382061 | C | T | 0.591 | -0.055 | 0.002 | 1.130E-161 | 734.396 |
| 337 | rs73963711 | T | C | 0.211 | 0.022 | 0.002 | 3.280E-19 | 80.358 |
| 338 | rs7423615 | T | C | 0.187 | 0.023 | 0.003 | 5.100E-20 | 84.031 |
| 339 | rs74299961 | T | C | 0.203 | 0.013 | 0.002 | 5.000E-08 | 29.763 |
| 340 | rs743002 | C | T | 0.066 | -0.059 | 0.004 | 2.900E-48 | 213.297 |
| 341 | rs74331768 | A | G | 0.098 | 0.019 | 0.003 | 7.490E-09 | 33.463 |
| 342 | rs7441808 | G | A | 0.301 | 0.014 | 0.002 | 1.220E-10 | 41.474 |
| 343 | rs74480102 | A | G | 0.043 | -0.083 | 0.005 | 2.520E-63 | 282.416 |
| 344 | rs74612091 | A | T | 0.063 | 0.061 | 0.004 | 1.230E-49 | 219.612 |
| 345 | rs746550 | T | C | 0.236 | 0.023 | 0.002 | 9.030E-23 | 96.579 |
| 346 | rs7569084 | T | C | 0.584 | 0.019 | 0.002 | 1.190E-20 | 86.965 |
| 347 | rs7636495 | A | G | 0.181 | -0.024 | 0.003 | 1.840E-20 | 86.093 |
| 348 | rs7646283 | T | C | 0.368 | 0.026 | 0.002 | 2.330E-36 | 158.786 |
| 349 | rs7646695 | T | C | 0.180 | 0.018 | 0.003 | 1.820E-11 | 45.213 |
| 350 | rs76474320 | C | T | 0.077 | 0.028 | 0.004 | 5.760E-14 | 56.544 |
| 351 | rs7649812 | G | C | 0.243 | 0.016 | 0.002 | 3.740E-12 | 48.328 |
| 352 | rs76639817 | T | C | 0.014 | 0.050 | 0.009 | 5.280E-09 | 34.135 |
| 353 | rs76793172 | T | C | 0.092 | -0.042 | 0.003 | 8.070E-34 | 147.117 |
| 354 | rs76830965 | A | C | 0.118 | -0.023 | 0.003 | 3.270E-14 | 57.642 |
| 355 | rs7687708 | G | T | 0.220 | -0.015 | 0.002 | 1.560E-09 | 36.514 |
| 356 | rs76908370 | G | A | 0.036 | -0.032 | 0.006 | 6.570E-09 | 33.702 |
| 357 | rs76981581 | G | C | 0.048 | -0.026 | 0.005 | 2.220E-08 | 31.344 |
| 358 | rs7700687 | T | C | 0.617 | 0.040 | 0.002 | 4.819E-86 | 386.802 |
| 359 | rs77625297 | C | G | 0.035 | -0.046 | 0.006 | 2.790E-16 | 67.049 |
| 360 | rs778756 | G | A | 0.580 | -0.016 | 0.002 | 1.570E-15 | 63.609 |
| 361 | rs7797428 | T | C | 0.469 | 0.012 | 0.002 | 5.850E-10 | 38.429 |
| 362 | rs783646 | C | G | 0.817 | -0.019 | 0.003 | 2.410E-13 | 53.701 |
| 363 | rs7839946 | C | T | 0.057 | 0.024 | 0.004 | 2.000E-08 | 31.541 |
| 364 | rs7840212 | T | C | 0.337 | -0.040 | 0.002 | 7.110E-81 | 363.054 |
| 365 | rs7846314 | T | A | 0.187 | -0.033 | 0.003 | 3.920E-38 | 166.879 |
| 366 | rs7868130 | T | C | 0.259 | 0.032 | 0.002 | 3.350E-46 | 203.775 |
| 367 | rs78691875 | A | C | 0.021 | -0.051 | 0.007 | 1.690E-13 | 54.417 |
| 368 | rs7897422 | C | T | 0.192 | 0.021 | 0.003 | 1.850E-16 | 67.864 |
| 369 | rs79716587 | A | G | 0.127 | -0.024 | 0.003 | 3.990E-16 | 66.317 |
| 370 | rs7975 | A | G | 0.324 | -0.012 | 0.002 | 1.170E-08 | 32.577 |
| 371 | rs7986796 | T | G | 0.628 | 0.024 | 0.002 | 4.910E-32 | 138.994 |
| 372 | rs8 | T | C | 0.204 | 0.022 | 0.003 | 7.630E-18 | 74.176 |
| 373 | rs80054178 | C | T | 0.022 | -0.082 | 0.007 | 8.570E-34 | 146.993 |
| 374 | rs80066203 | T | C | 0.068 | -0.024 | 0.004 | 8.530E-10 | 37.689 |
| 375 | rs8012643 | T | C | 0.286 | 0.015 | 0.002 | 1.910E-12 | 49.659 |
| 376 | rs8020739 | T | G | 0.644 | 0.025 | 0.002 | 2.570E-32 | 140.184 |
| 377 | rs8026803 | C | T | 0.261 | -0.031 | 0.002 | 1.670E-43 | 191.440 |
| 378 | rs8044920 | T | C | 0.376 | 0.012 | 0.002 | 3.590E-09 | 34.884 |
| 379 | rs8050508 | T | C | 0.031 | 0.035 | 0.006 | 1.010E-09 | 37.367 |
| 380 | rs8061729 | C | T | 0.274 | -0.013 | 0.002 | 9.320E-09 | 33.012 |
| 381 | rs8076052 | C | A | 0.296 | -0.018 | 0.002 | 4.330E-17 | 70.737 |
| 382 | rs8108623 | A | C | 0.625 | 0.018 | 0.002 | 1.910E-16 | 67.794 |
| 383 | rs8142080 | T | G | 0.102 | -0.024 | 0.003 | 5.720E-13 | 52.010 |
| 384 | rs884634 | C | T | 0.415 | 0.012 | 0.002 | 6.830E-10 | 38.118 |
| 385 | rs911603 | A | C | 0.404 | -0.025 | 0.002 | 1.420E-33 | 145.944 |
| 386 | rs925966 | G | C | 0.759 | -0.018 | 0.002 | 1.320E-14 | 59.438 |
| 387 | rs9349322 | C | A | 0.147 | 0.022 | 0.003 | 4.040E-15 | 61.765 |
| 388 | rs9389268 | G | A | 0.256 | -0.045 | 0.002 | 3.690E-87 | 391.786 |
| 389 | rs9392525 | C | T | 0.576 | 0.013 | 0.002 | 6.950E-11 | 42.597 |
| 390 | rs941616 | T | C | 0.610 | -0.012 | 0.002 | 6.690E-09 | 33.662 |
| 391 | rs954954 | C | A | 0.105 | -0.035 | 0.003 | 3.570E-27 | 116.719 |
| 392 | rs962993 | T | C | 0.422 | -0.040 | 0.002 | 4.920E-90 | 405.072 |
| 393 | rs964184 | C | G | 0.867 | 0.029 | 0.003 | 4.390E-23 | 98.032 |
| 394 | rs9666598 | G | C | 0.843 | -0.020 | 0.003 | 1.360E-12 | 50.319 |
| 395 | rs9675999 | A | G | 0.627 | 0.016 | 0.002 | 1.590E-15 | 63.588 |
| 396 | rs9815874 | T | C | 0.300 | 0.028 | 0.002 | 1.930E-39 | 172.854 |
| 397 | rs9818987 | T | C | 0.306 | 0.014 | 0.002 | 2.600E-10 | 40.023 |
| 398 | rs9835307 | C | T | 0.658 | 0.026 | 0.002 | 2.690E-33 | 144.681 |
| 399 | rs9837045 | A | G | 0.455 | 0.011 | 0.002 | 1.410E-08 | 32.233 |
| 400 | rs9840310 | G | A | 0.735 | -0.013 | 0.002 | 1.300E-08 | 32.387 |
| 401 | rs9872485 | G | T | 0.164 | -0.018 | 0.003 | 9.260E-12 | 46.554 |
| 402 | rs9880192 | C | G | 0.411 | 0.042 | 0.002 | 9.080E-96 | 431.596 |
| 403 | rs9894839 | C | T | 0.635 | 0.012 | 0.002 | 1.330E-08 | 32.356 |
| 404 | rs9939774 | T | C | 0.405 | -0.029 | 0.002 | 1.980E-47 | 209.475 |
| 405 | rs9979383 | T | C | 0.630 | 0.028 | 0.002 | 8.000E-43 | 188.394 |

SNPs: single-nucleotide polymorphisms; eaf: effect allele frequency; se: standard error

Supplementary Table 6: MR-PRESSO analysis for estimate of EOS-IgG.

| MR. Analysis | Causal. Estimate | Sd | T.stat | p |
| --- | --- | --- | --- | --- |
| Raw | 0.0189556 | 0.0085864 | 2.2076232 | 0.0278836 |
| Outlier-corrected | NA | NA | NA | NA |

MR-PRESSO: MR Pleiotropy RESidual Sum and Outlier; EOS: eosinophil; Sd: standard deviation; NA: Not Applicable

Supplementary Table 7: MR-PRESSO analysis for estimate of IgG-Asthma.

| MR. Analysis | Causal. Estimate | Sd | T.stat | p |
| --- | --- | --- | --- | --- |
| Raw | 0.7162654 | 0.3024139 | 2.3684937 | 0.0354986 |
| Outlier-corrected | NA | NA | NA | NA |

MR-PRESSO: MR Pleiotropy RESidual Sum and Outlier; Sd: standard deviation; Not Applicable

Supplementary Table 8: MR-PRESSO analysis for estimate of EOS-Asthma.

| MR. Analysis | Causal. Estimate | Sd | T.stat | p |
| --- | --- | --- | --- | --- |
| Raw | 0.6131714 | 0.0667605 | 9.1846468 | 2.0209477E-18 |
| Outlier-corrected | 0.6107223 | 0.0616575 | 9.9050792 | 7.5211378E-21 |

MR-PRESSO: MR Pleiotropy RESidual Sum and Outlier; Sd: standard deviation; Not Applicable

Supplementary Table 9: Single SNP analysis for estimate of EOS-IgG.

|  | SNPs | beta | se | p |
| --- | --- | --- | --- | --- |
| 1 | rs1004870 | -0.00318891 | 0.0035587 | 0.37042305 |
| 2 | rs10059018 | -0.00409141 | 0.00465614 | 0.37977118 |
| 3 | rs10062687 | -0.00481072 | 0.00418703 | 0.25085225 |
| 4 | rs10100356 | 0.00590796 | 0.00443071 | 0.18270398 |
| 5 | rs10165678 | -0.0002111 | 0.0041482 | 0.95942493 |
| 6 | rs10174238 | 0.00255896 | 0.00428587 | 0.55059847 |
| 7 | rs10195713 | -0.00055258 | 0.00531287 | 0.91718406 |
| 8 | rs1036332 | -0.00395704 | 0.00383707 | 0.30266904 |
| 9 | rs1037674 | -8.0353E-05 | 0.00381412 | 0.98319599 |
| 10 | rs1039341 | 0.00456157 | 0.00383157 | 0.23412727 |
| 11 | rs10472984 | -0.00530985 | 0.00395644 | 0.17987881 |
| 12 | rs1047891 | -0.00706506 | 0.00385363 | 0.06705019 |
| 13 | rs1057258 | 0.00185459 | 0.00464073 | 0.68951386 |
| 14 | rs1059091 | 0.00459785 | 0.00369467 | 0.21362693 |
| 15 | rs10745763 | -0.00139783 | 0.00355948 | 0.69462275 |
| 16 | rs10777378 | -0.00484978 | 0.00345607 | 0.16085294 |
| 17 | rs10782957 | 0.00185862 | 0.00369943 | 0.61549142 |
| 18 | rs10876550 | -0.00625962 | 0.00353086 | 0.07656495 |
| 19 | rs10900595 | -0.00510494 | 0.00388586 | 0.18924482 |
| 20 | rs10930337 | -0.00105512 | 0.00377611 | 0.77998096 |
| 21 | rs10962640 | -0.00382065 | 0.00400096 | 0.33984431 |
| 22 | rs1099448 | 0.00084296 | 0.00351166 | 0.81034396 |
| 23 | rs10995240 | -0.00490007 | 0.00361213 | 0.17523102 |
| 24 | rs11065822 | 0.00639614 | 0.00362335 | 0.07782874 |
| 25 | rs11071528 | 0.00339317 | 0.0044508 | 0.4460176 |
| 26 | rs11071559 | -0.00520874 | 0.00513025 | 0.31021229 |
| 27 | rs11079340 | 0.00278623 | 0.00385239 | 0.46969939 |
| 28 | rs11088236 | 0.00272705 | 0.00370897 | 0.46235653 |
| 29 | rs111759324 | -0.0106206 | 0.00579479 | 0.06713516 |
| 30 | rs112036266 | -0.00140223 | 0.00441011 | 0.75058348 |
| 31 | rs11204682 | 0.00628174 | 0.00418431 | 0.13360786 |
| 32 | rs11236813 | -0.00072629 | 0.00568458 | 0.89836102 |
| 33 | rs11255507 | -0.00394081 | 0.0048165 | 0.4134462 |
| 34 | rs113105190 | -1.5257E-05 | 0.00692825 | 0.998243 |
| 35 | rs113542380 | -0.0145531 | 0.00661823 | 0.02811318 |
| 36 | rs11555542 | -0.0118649 | 0.0065332 | 0.06967228 |
| 37 | rs11571404 | -0.011821 | 0.00448734 | 0.00856288 |
| 38 | rs11578794 | 4.0537E-06 | 0.00369041 | 0.999124 |
| 39 | rs11628569 | -0.00531819 | 0.00389647 | 0.17260286 |
| 40 | rs11647946 | -0.00354151 | 0.003758 | 0.34622307 |
| 41 | rs11684770 | -0.00228443 | 0.00384321 | 0.55237625 |
| 42 | rs11695281 | 0.00461011 | 0.00357104 | 0.19701577 |
| 43 | rs11701475 | -0.00390343 | 0.00413937 | 0.3459107 |
| 44 | rs11702918 | 0.00096519 | 0.00545161 | 0.85950894 |
| 45 | rs1170439 | -0.00598206 | 0.00406695 | 0.14163807 |
| 46 | rs117068593 | 0.00033308 | 0.00458442 | 0.94209609 |
| 47 | rs1178016 | -0.00357412 | 0.00344778 | 0.30015598 |
| 48 | rs11786536 | 0.00089022 | 0.00498164 | 0.85821006 |
| 49 | rs117955557 | -0.00039148 | 0.00419755 | 0.92571393 |
| 50 | rs118013485 | -0.00841582 | 0.00706835 | 0.23408307 |
| 51 | rs11869228 | -0.00155455 | 0.00469171 | 0.74045897 |
| 52 | rs11886928 | 0.00099788 | 0.00376156 | 0.79084614 |
| 53 | rs11931711 | 0.0065423 | 0.0040305 | 0.10486607 |
| 54 | rs12100034 | 0.00250492 | 0.00368548 | 0.4968714 |
| 55 | rs12154498 | 0.0114854 | 0.00498561 | 0.02144618 |
| 56 | rs121564 | -0.00277436 | 0.00378685 | 0.46395942 |
| 57 | rs12208103 | -0.00742803 | 0.00364716 | 0.04195078 |
| 58 | rs12361586 | -0.00614513 | 0.00567916 | 0.27949433 |
| 59 | rs12408934 | -0.0101344 | 0.00595034 | 0.08885257 |
| 60 | rs12470046 | -0.00720752 | 0.00374141 | 0.05433879 |
| 61 | rs12487980 | 0.0032394 | 0.00381249 | 0.39570907 |
| 62 | rs12530946 | 0.00456607 | 0.00359295 | 0.20408402 |
| 63 | rs12540285 | 0.00069235 | 0.00426808 | 0.87116998 |
| 64 | rs12545733 | 0.00532243 | 0.00387294 | 0.1696731 |
| 65 | rs1257192 | -0.00085032 | 0.00455673 | 0.85200707 |
| 66 | rs12581511 | -0.00027235 | 0.00499901 | 0.95656393 |
| 67 | rs12681644 | -0.00225844 | 0.00437426 | 0.60575917 |
| 68 | rs12705849 | 0.00183087 | 0.00355601 | 0.60676148 |
| 69 | rs12820863 | 0.00300938 | 0.00364848 | 0.40966607 |
| 70 | rs12878610 | 0.00026096 | 0.00348367 | 0.94030295 |
| 71 | rs12882281 | -0.00031536 | 0.00418261 | 0.93991396 |
| 72 | rs12941068 | 0.00258458 | 0.0037818 | 0.49449852 |
| 73 | rs1296535 | 0.00450553 | 0.00424158 | 0.28839186 |
| 74 | rs12978850 | -0.0102241 | 0.00482084 | 0.03418613 |
| 75 | rs13073683 | -0.00715538 | 0.00363125 | 0.04906028 |
| 76 | rs13105682 | 0.00032737 | 0.00742274 | 0.96483089 |
| 77 | rs13120371 | -0.00157265 | 0.00366238 | 0.66771941 |
| 78 | rs13138355 | -0.00140808 | 0.00454818 | 0.75693746 |
| 79 | rs13139941 | 0.00689108 | 0.00437717 | 0.11573294 |
| 80 | rs13207791 | 0.00465372 | 0.0064353 | 0.46975564 |
| 81 | rs13226583 | -0.00236281 | 0.00557987 | 0.67205683 |
| 82 | rs1323650 | -0.00231267 | 0.00371518 | 0.53376243 |
| 83 | rs13511 | -0.00726616 | 0.00356579 | 0.04184177 |
| 84 | rs1353286 | 0.00400282 | 0.00359134 | 0.26530412 |
| 85 | rs1365623 | 0.00278667 | 0.0037415 | 0.45656856 |
| 86 | rs1395269 | 0.00053961 | 0.00380966 | 0.88739198 |
| 87 | rs139640694 | 0.00338715 | 0.00587517 | 0.5643948 |
| 88 | rs1406449 | -0.00055581 | 0.00356762 | 0.87622694 |
| 89 | rs1414517 | 0.00291199 | 0.00440464 | 0.50869094 |
| 90 | rs1427499 | 0.00181302 | 0.00387412 | 0.63990131 |
| 91 | rs144569746 | 0.0131664 | 0.00553502 | 0.01756143 |
| 92 | rs1448187 | -0.00656294 | 0.00392962 | 0.09521163 |
| 93 | rs14713 | -0.00193997 | 0.00452636 | 0.6683116 |
| 94 | rs1471816 | 0.00510576 | 0.00354273 | 0.14984595 |
| 95 | rs1479918 | 0.0018444 | 0.00401622 | 0.64616436 |
| 96 | rs1519602 | 0.00917551 | 0.00373876 | 0.01429322 |
| 97 | rs1529745 | 0.00243692 | 0.00452244 | 0.59011139 |
| 98 | rs1539174 | 0.00089489 | 0.00415472 | 0.82950709 |
| 99 | rs1547258 | 0.00064745 | 0.0037184 | 0.86180597 |
| 100 | rs159963 | 0.00215233 | 0.00360066 | 0.55013718 |
| 101 | rs1672753 | -0.00152278 | 0.0047098 | 0.74651923 |
| 102 | rs1684578 | 0.00445091 | 0.00369292 | 0.22839416 |
| 103 | rs1689510 | -0.00217103 | 0.0036875 | 0.5561616 |
| 104 | rs16956811 | -0.00363176 | 0.00686442 | 0.59687584 |
| 105 | rs17061503 | 0.00075478 | 0.00393715 | 0.84801103 |
| 106 | rs17175830 | -0.00186254 | 0.00413886 | 0.65279978 |
| 107 | rs17293632 | -0.00166476 | 0.00424846 | 0.69525641 |
| 108 | rs174548 | -0.00292506 | 0.00398239 | 0.46281881 |
| 109 | rs17482472 | -0.0162924 | 0.00570546 | 0.00438571 |
| 110 | rs17516457 | -0.00530179 | 0.0035842 | 0.13940296 |
| 111 | rs175705 | 0.00019624 | 0.00376046 | 0.95839204 |
| 112 | rs17653687 | 0.00286878 | 0.00495138 | 0.56245787 |
| 113 | rs17668272 | -0.00850731 | 0.00550169 | 0.12235001 |
| 114 | rs17682575 | 0.00214005 | 0.00445949 | 0.6314137 |
| 115 | rs17689159 | -0.00483023 | 0.00372651 | 0.19521716 |
| 116 | rs17849501 | 0.00676068 | 0.00819977 | 0.4098661 |
| 117 | rs1800692 | 0.00574742 | 0.00350679 | 0.10154394 |
| 118 | rs180506 | -0.00473785 | 0.00413708 | 0.25239689 |
| 119 | rs1828803 | 0.0006932 | 0.00358208 | 0.84659208 |
| 120 | rs1861489 | -0.00147114 | 0.00421144 | 0.72692399 |
| 121 | rs201798 | -0.0049096 | 0.00367894 | 0.18234296 |
| 122 | rs2025489 | -6.512E-05 | 0.00363347 | 0.985704 |
| 123 | rs2089979 | -0.0040012 | 0.00360911 | 0.26785577 |
| 124 | rs214080 | -0.00369984 | 0.00365509 | 0.31167 |
| 125 | rs2182885 | 0.0003101 | 0.0036216 | 0.93178111 |
| 126 | rs2223043 | -0.00589825 | 0.00388511 | 0.12930699 |
| 127 | rs2228467 | -0.00113206 | 0.00732139 | 0.87715108 |
| 128 | rs2239633 | -0.0003135 | 0.00370867 | 0.93265109 |
| 129 | rs2253427 | 0.00175275 | 0.00458002 | 0.70203376 |
| 130 | rs2399441 | -0.00298846 | 0.00357922 | 0.40394943 |
| 131 | rs2410732 | 0.00086819 | 0.00391898 | 0.82472204 |
| 132 | rs2419313 | -0.00038979 | 0.00470335 | 0.93396693 |
| 133 | rs2431097 | 0.00214019 | 0.00358272 | 0.55040073 |
| 134 | rs2497318 | -0.00503785 | 0.00352028 | 0.15272003 |
| 135 | rs2502995 | -0.0024994 | 0.00355832 | 0.48258853 |
| 136 | rs2505521 | -0.00771366 | 0.00453584 | 0.08933261 |
| 137 | rs2566133 | 0.00703839 | 0.0035686 | 0.04885286 |
| 138 | rs2579505 | 0.00685864 | 0.00363516 | 0.05948672 |
| 139 | rs2646438 | 0.00362748 | 0.0035428 | 0.30613219 |
| 140 | rs2713548 | 0.00576408 | 0.00364669 | 0.11428099 |
| 141 | rs2788211 | 0.00244178 | 0.0044059 | 0.57956213 |
| 142 | rs2807740 | -0.00359728 | 0.00443218 | 0.41719971 |
| 143 | rs2817377 | 0.00239836 | 0.00357097 | 0.50197952 |
| 144 | rs28362902 | -0.00311246 | 0.00526211 | 0.55432951 |
| 145 | rs2838317 | 0.00672308 | 0.00349428 | 0.05463865 |
| 146 | rs28383314 | -0.0129311 | 0.00365617 | 0.00042386 |
| 147 | rs28421324 | 0.00079677 | 0.00555348 | 0.8859461 |
| 148 | rs2847266 | 0.00268495 | 0.00381737 | 0.48200329 |
| 149 | rs2850542 | 0.0012643 | 0.00359498 | 0.72515359 |
| 150 | rs28532037 | 0.00409034 | 0.00561987 | 0.46688616 |
| 151 | rs2864936 | -0.00163395 | 0.00465265 | 0.72552269 |
| 152 | rs2887502 | -0.00495903 | 0.00357685 | 0.16593118 |
| 153 | rs2894401 | 0.00553814 | 0.00393429 | 0.15954603 |
| 154 | rs290430 | -0.0104675 | 0.0037094 | 0.00487001 |
| 155 | rs2920505 | -0.00045811 | 0.00352023 | 0.89648598 |
| 156 | rs295 | -0.00848401 | 0.00414047 | 0.04072115 |
| 157 | rs295273 | 0.0043353 | 0.00403658 | 0.28308314 |
| 158 | rs2979489 | 0.00284693 | 0.00401039 | 0.47793958 |
| 159 | rs2992333 | -0.00441067 | 0.00355122 | 0.21452504 |
| 160 | rs301162 | -0.00167871 | 0.00472059 | 0.72220418 |
| 161 | rs3024971 | -0.00111547 | 0.00561508 | 0.84257196 |
| 162 | rs3093023 | 0.00901158 | 0.00362022 | 0.01297329 |
| 163 | rs3096309 | 0.00555061 | 0.00477714 | 0.24555173 |
| 164 | rs3110791 | 0.00019306 | 0.00361437 | 0.95741406 |
| 165 | rs3218148 | -0.0048948 | 0.00357186 | 0.17087991 |
| 166 | rs33982662 | -0.00030812 | 0.00405439 | 0.93943795 |
| 167 | rs34212866 | -0.00531395 | 0.00427128 | 0.21375289 |
| 168 | rs34290285 | -0.00429706 | 0.00399227 | 0.28203629 |
| 169 | rs34363176 | 0.00381885 | 0.00410561 | 0.35251938 |
| 170 | rs34448954 | 0.00154254 | 0.00599838 | 0.7971121 |
| 171 | rs34466956 | 0.00391205 | 0.00354381 | 0.26990069 |
| 172 | rs34495 | 0.00101367 | 0.00379088 | 0.78921985 |
| 173 | rs34505104 | -0.00248905 | 0.00395428 | 0.52919533 |
| 174 | rs34631302 | -0.005018 | 0.00389592 | 0.19804186 |
| 175 | rs346835 | -0.0031804 | 0.00397283 | 0.42359224 |
| 176 | rs350836 | 0.00050032 | 0.00382011 | 0.89582504 |
| 177 | rs35249183 | 0.0073038 | 0.00610874 | 0.23212825 |
| 178 | rs35409523 | -0.00509499 | 0.00668387 | 0.44607409 |
| 179 | rs36084354 | -0.00295662 | 0.00575447 | 0.60751639 |
| 180 | rs3731211 | -0.00493037 | 0.00397724 | 0.21540015 |
| 181 | rs3742704 | 0.00328753 | 0.00603515 | 0.58606664 |
| 182 | rs3746420 | 0.00434812 | 0.00757568 | 0.56612717 |
| 183 | rs3747869 | -0.00216933 | 0.00651663 | 0.73929029 |
| 184 | rs3757114 | -0.00252087 | 0.00352854 | 0.47513607 |
| 185 | rs3785356 | -0.00783526 | 0.00378947 | 0.03893409 |
| 186 | rs3786586 | -0.00501435 | 0.00507367 | 0.3232429 |
| 187 | rs3790163 | 0.0108057 | 0.0042521 | 0.01119747 |
| 188 | rs3804590 | 0.00165822 | 0.00380568 | 0.66313355 |
| 189 | rs3812206 | -0.00115894 | 0.0038292 | 0.76221238 |
| 190 | rs3823536 | 0.0042508 | 0.0035732 | 0.23447687 |
| 191 | rs3824867 | -0.00350333 | 0.0039167 | 0.37129406 |
| 192 | rs3846855 | 0.0110787 | 0.00464519 | 0.01726792 |
| 193 | rs3850107 | 0.00123711 | 0.00375055 | 0.7415834 |
| 194 | rs3950296 | 0.00374528 | 0.00422281 | 0.37534013 |
| 195 | rs397187 | -0.00402317 | 0.00356414 | 0.25926029 |
| 196 | rs4074672 | 0.00182452 | 0.00359549 | 0.61195431 |
| 197 | rs410867 | 0.00718594 | 0.0042755 | 0.09313223 |
| 198 | rs412884 | 0.00485621 | 0.00367944 | 0.18720007 |
| 199 | rs4142528 | -0.00111235 | 0.00354966 | 0.75406538 |
| 200 | rs4148757 | 0.00021489 | 0.00417839 | 0.95899402 |
| 201 | rs4240624 | -0.00618239 | 0.00656549 | 0.34660195 |
| 202 | rs4280242 | -0.00483677 | 0.00396647 | 0.22297799 |
| 203 | rs4310436 | -0.00127629 | 0.00551275 | 0.81696208 |
| 204 | rs4347868 | 0.00071152 | 0.00405779 | 0.86084309 |
| 205 | rs4409785 | 0.00103064 | 0.00455155 | 0.82090708 |
| 206 | rs460631 | -0.0028285 | 0.00511413 | 0.58033665 |
| 207 | rs4618204 | 0.00017942 | 0.00348671 | 0.95897194 |
| 208 | rs4652560 | 0.00074558 | 0.00358967 | 0.83550509 |
| 209 | rs4675190 | 0.00240639 | 0.00351327 | 0.49353959 |
| 210 | rs4680250 | -0.0047667 | 0.00382212 | 0.21264296 |
| 211 | rs4703589 | 0.00185049 | 0.00362582 | 0.60990894 |
| 212 | rs4703730 | 0.00519123 | 0.00356524 | 0.14569109 |
| 213 | rs4721559 | 0.00034434 | 0.00400957 | 0.93158009 |
| 214 | rs4722171 | -0.00066094 | 0.00361714 | 0.85505096 |
| 215 | rs473739 | 0.00170998 | 0.00386953 | 0.65865046 |
| 216 | rs4746153 | 0.00555977 | 0.00465505 | 0.23262586 |
| 217 | rs4849903 | -0.00178376 | 0.00378376 | 0.63744248 |
| 218 | rs4870977 | -0.00036207 | 0.00529415 | 0.94548795 |
| 219 | rs4908835 | 0.00094428 | 0.00498479 | 0.8497931 |
| 220 | rs4931002 | -0.00251372 | 0.00421155 | 0.55073541 |
| 221 | rs495149 | 0.00393979 | 0.00478984 | 0.41097272 |
| 222 | rs496475 | -4.5234E-05 | 0.0035624 | 0.989872 |
| 223 | rs556063 | -0.00461321 | 0.00358489 | 0.19844904 |
| 224 | rs55868524 | 0.00883377 | 0.00364138 | 0.01544685 |
| 225 | rs55879743 | 0.00689727 | 0.00775173 | 0.37380491 |
| 226 | rs55977204 | -0.00113645 | 0.00588862 | 0.84700603 |
| 227 | rs56117721 | 0.00527495 | 0.00707305 | 0.45597707 |
| 228 | rs56179563 | -0.00671188 | 0.00361888 | 0.06393961 |
| 229 | rs56268488 | 0.00052361 | 0.00640758 | 0.93488889 |
| 230 | rs56330463 | -0.00225007 | 0.00360537 | 0.53271264 |
| 231 | rs574183 | -0.00151092 | 0.00371604 | 0.6843937 |
| 232 | rs5747308 | 0.00312853 | 0.00350361 | 0.37210542 |
| 233 | rs57633475 | 0.00097349 | 0.00553971 | 0.86054305 |
| 234 | rs57834782 | 0.0016998 | 0.00427533 | 0.6910222 |
| 235 | rs58745116 | 0.00359482 | 0.00356969 | 0.31416297 |
| 236 | rs58833930 | 0.00082127 | 0.00575281 | 0.8865089 |
| 237 | rs594479 | -0.00661521 | 0.00400278 | 0.09871883 |
| 238 | rs60175411 | -0.00600734 | 0.00477301 | 0.20847021 |
| 239 | rs60600003 | 0.00466322 | 0.00549083 | 0.39593418 |
| 240 | rs6080761 | 0.00407489 | 0.00349671 | 0.24415915 |
| 241 | rs6103572 | -0.00152899 | 0.00409679 | 0.70906655 |
| 242 | rs6139104 | 0.00478416 | 0.00637426 | 0.45310619 |
| 243 | rs6141755 | -0.00212669 | 0.00410743 | 0.60473764 |
| 244 | rs61426394 | 0.0124194 | 0.00640083 | 0.05262959 |
| 245 | rs61798836 | -0.00491717 | 0.00489105 | 0.31497929 |
| 246 | rs62011287 | 0.00070602 | 0.00368309 | 0.84802294 |
| 247 | rs62061733 | 0.00114259 | 0.00405586 | 0.77822289 |
| 248 | rs62086903 | -0.00428746 | 0.00413258 | 0.29976712 |
| 249 | rs62105489 | 0.0153337 | 0.00801132 | 0.05590878 |
| 250 | rs62183994 | -0.00371334 | 0.00800724 | 0.64293046 |
| 251 | rs62308111 | 0.0040383 | 0.00404326 | 0.31814932 |
| 252 | rs62385501 | -0.00016015 | 0.00382988 | 0.96665299 |
| 253 | rs62408224 | 0.00078005 | 0.0036469 | 0.83067301 |
| 254 | rs62420764 | 0.00466104 | 0.00503726 | 0.35502914 |
| 255 | rs62473720 | 0.00087603 | 0.00383519 | 0.81936706 |
| 256 | rs62539154 | 0.0090901 | 0.00604551 | 0.13300103 |
| 257 | rs634534 | 0.0018247 | 0.00353687 | 0.60603401 |
| 258 | rs637064 | 0.00144719 | 0.00355047 | 0.68365029 |
| 259 | rs6479336 | 0.00877804 | 0.0045838 | 0.05577891 |
| 260 | rs6496717 | -0.00612975 | 0.0041506 | 0.14003803 |
| 261 | rs6540985 | 0.00208834 | 0.00379233 | 0.5819826 |
| 262 | rs6556313 | 0.00265125 | 0.0037549 | 0.48030488 |
| 263 | rs6573020 | 0.00819082 | 0.00360799 | 0.023411 |
| 264 | rs6684992 | 0.00590874 | 0.00528727 | 0.2640347 |
| 265 | rs6691839 | 0.00028081 | 0.00410631 | 0.94549296 |
| 266 | rs6731125 | -0.00400103 | 0.00351361 | 0.25509386 |
| 267 | rs6750754 | -0.00315652 | 0.00398692 | 0.42871532 |
| 268 | rs67856193 | -0.00350701 | 0.00374099 | 0.34875384 |
| 269 | rs6787336 | -0.00214987 | 0.00395025 | 0.5864014 |
| 270 | rs6904506 | -0.00479138 | 0.00630433 | 0.44742681 |
| 271 | rs6924350 | 0.00293834 | 0.00441943 | 0.50629076 |
| 272 | rs6924387 | 0.00569078 | 0.00352319 | 0.10657909 |
| 273 | rs6930635 | -0.0123949 | 0.00599833 | 0.03905171 |
| 274 | rs6956283 | -0.00262186 | 0.00380841 | 0.49133647 |
| 275 | rs6971710 | 0.00220909 | 0.00440761 | 0.61634234 |
| 276 | rs6979947 | -0.00114417 | 0.00400349 | 0.77509512 |
| 277 | rs6986109 | 0.00144389 | 0.00377931 | 0.70250594 |
| 278 | rs6989099 | -0.00084338 | 0.00361214 | 0.81543303 |
| 279 | rs699664 | -0.00307404 | 0.00385827 | 0.4258023 |
| 280 | rs6999452 | 0.00218402 | 0.00355845 | 0.53951808 |
| 281 | rs7026022 | 0.00024073 | 0.0035864 | 0.946498 |
| 282 | rs708776 | 0.00951636 | 0.00707577 | 0.17897402 |
| 283 | rs7123726 | -0.00193965 | 0.00416839 | 0.64180338 |
| 284 | rs7141943 | -0.00566089 | 0.00370977 | 0.12734503 |
| 285 | rs71429414 | -0.0023066 | 0.00434441 | 0.59558397 |
| 286 | rs7158239 | 0.00819561 | 0.00358804 | 0.02257408 |
| 287 | rs71628184 | -0.00327843 | 0.00597499 | 0.58334167 |
| 288 | rs7173571 | 0.00350276 | 0.0034191 | 0.30586515 |
| 289 | rs7215391 | -0.0020749 | 0.00413452 | 0.61588553 |
| 290 | rs725613 | 0.006172 | 0.00370711 | 0.09624762 |
| 291 | rs7257 | 0.00182504 | 0.00362163 | 0.61442942 |
| 292 | rs72766638 | 7.5196E-05 | 0.00513758 | 0.988325 |
| 293 | rs72844043 | -0.00018114 | 0.00354671 | 0.95927803 |
| 294 | rs72987040 | -0.00076551 | 0.00551079 | 0.88954803 |
| 295 | rs72998585 | -0.00272229 | 0.00511109 | 0.59441259 |
| 296 | rs73049239 | -0.00254071 | 0.00693437 | 0.71414928 |
| 297 | rs73118830 | -0.00509869 | 0.00642437 | 0.42759144 |
| 298 | rs73176183 | -0.00019399 | 0.00527318 | 0.97066194 |
| 299 | rs73176685 | -0.00092888 | 0.0041901 | 0.82460507 |
| 300 | rs73187852 | -0.00078548 | 0.00386869 | 0.83914904 |
| 301 | rs73203442 | -0.00611612 | 0.00532788 | 0.25126905 |
| 302 | rs73232881 | 0.00421263 | 0.00408375 | 0.30253108 |
| 303 | rs73238201 | -0.00557563 | 0.00481279 | 0.24693918 |
| 304 | rs73272842 | 0.00674888 | 0.00529992 | 0.20317815 |
| 305 | rs7327960 | 0.00029695 | 0.00440451 | 0.946262 |
| 306 | rs73322872 | -0.00733978 | 0.00419553 | 0.08052672 |
| 307 | rs73428834 | -0.00515471 | 0.00701288 | 0.46249176 |
| 308 | rs7354779 | 0.00030516 | 0.00402916 | 0.9396439 |
| 309 | rs7382061 | -0.00296045 | 0.00353122 | 0.40202772 |
| 310 | rs73963711 | 0.00257633 | 0.00422106 | 0.54177006 |
| 311 | rs7423615 | -0.00699284 | 0.00471356 | 0.13824486 |
| 312 | rs74299961 | -0.00314018 | 0.00445549 | 0.48110847 |
| 313 | rs743002 | -0.0107099 | 0.00721459 | 0.13799997 |
| 314 | rs74331768 | 0.00333122 | 0.00541738 | 0.53875337 |
| 315 | rs7441808 | -0.00150534 | 0.00370374 | 0.68450874 |
| 316 | rs746550 | -0.00224146 | 0.00417806 | 0.59174555 |
| 317 | rs7569084 | 0.00464178 | 0.00348126 | 0.18271996 |
| 318 | rs7636495 | -0.0012879 | 0.00484307 | 0.79035281 |
| 319 | rs7646283 | 0.00516716 | 0.00366858 | 0.15930008 |
| 320 | rs7646695 | -0.00522088 | 0.00493001 | 0.28985914 |
| 321 | rs76474320 | -0.00343103 | 0.00669598 | 0.60848517 |
| 322 | rs7649812 | -0.00030587 | 0.00406122 | 0.93997997 |
| 323 | rs76793172 | -0.0059031 | 0.00642859 | 0.35870877 |
| 324 | rs76830965 | 0.00177585 | 0.00584478 | 0.76131783 |
| 325 | rs7687708 | 0.00462555 | 0.00420744 | 0.27187295 |
| 326 | rs76981581 | -0.00559898 | 0.00792501 | 0.48004727 |
| 327 | rs7700687 | -0.00343203 | 0.00368807 | 0.3523011 |
| 328 | rs778756 | -0.00177093 | 0.00353989 | 0.6169884 |
| 329 | rs7797428 | 0.00285336 | 0.00341746 | 0.40395501 |
| 330 | rs783646 | 0.00721829 | 0.00432683 | 0.09558065 |
| 331 | rs7839946 | -0.00481566 | 0.00792365 | 0.54348805 |
| 332 | rs7840212 | -0.00076887 | 0.00374739 | 0.83747702 |
| 333 | rs7846314 | -0.00210699 | 0.00460836 | 0.64761962 |
| 334 | rs7868130 | -0.00574736 | 0.00414372 | 0.16575285 |
| 335 | rs7897422 | -0.00476969 | 0.00459538 | 0.29955598 |
| 336 | rs79716587 | 0.00553282 | 0.00517215 | 0.28500075 |
| 337 | rs7975 | -0.00516329 | 0.00393943 | 0.19028914 |
| 338 | rs7986796 | -0.00683115 | 0.00367208 | 0.06314079 |
| 339 | rs79881201 | 0.00205833 | 0.00359647 | 0.56723497 |
| 340 | rs8 | 0.00293892 | 0.00447828 | 0.5118091 |
| 341 | rs80066203 | 0.0131701 | 0.00729142 | 0.07118525 |
| 342 | rs8012643 | 0.00543464 | 0.00385135 | 0.1585291 |
| 343 | rs8020739 | -0.0006708 | 0.00375366 | 0.85820591 |
| 344 | rs8026803 | -0.00280705 | 0.0039408 | 0.47644525 |
| 345 | rs8044920 | -0.00355671 | 0.00369388 | 0.33585127 |
| 346 | rs8061729 | 0.00509215 | 0.00388791 | 0.1905882 |
| 347 | rs8076052 | -0.00276729 | 0.0038056 | 0.46730024 |
| 348 | rs8108623 | -0.00200706 | 0.0036522 | 0.58275365 |
| 349 | rs8142080 | 0.00158132 | 0.0055173 | 0.77447071 |
| 350 | rs884634 | -0.00068475 | 0.00360584 | 0.84942492 |
| 351 | rs911603 | -0.00197182 | 0.0035781 | 0.58170259 |
| 352 | rs925966 | -0.00317169 | 0.00396478 | 0.42392302 |
| 353 | rs9349322 | 0.00158065 | 0.00474098 | 0.73890227 |
| 354 | rs9389268 | 0.00217947 | 0.00410383 | 0.59548112 |
| 355 | rs9392525 | 0.00734221 | 0.00358535 | 0.0408404 |
| 356 | rs941616 | -0.00486589 | 0.00363423 | 0.18090893 |
| 357 | rs954954 | -9.6114E-06 | 0.00584373 | 0.998688 |
| 358 | rs962993 | -0.00540337 | 0.00350964 | 0.12398296 |
| 359 | rs964184 | 0.00226797 | 0.00548769 | 0.67949146 |
| 360 | rs9666598 | 0.00056968 | 0.00463404 | 0.902185 |
| 361 | rs9675999 | 0.00284169 | 0.00358627 | 0.42832853 |
| 362 | rs9815874 | -0.00035184 | 0.00389594 | 0.9280601 |
| 363 | rs9818987 | -0.00454643 | 0.00381615 | 0.23379596 |
| 364 | rs9835307 | 0.00043515 | 0.00362675 | 0.90452005 |
| 365 | rs9837045 | -0.00077384 | 0.00360115 | 0.82990007 |
| 366 | rs9840310 | -0.00226108 | 0.00402515 | 0.57442191 |
| 367 | rs9880192 | -0.00374289 | 0.00369434 | 0.31124114 |
| 368 | rs9889262 | -0.00381132 | 0.00356599 | 0.28542302 |
| 369 | rs9894839 | -0.00105832 | 0.00366851 | 0.77303472 |
| 370 | rs9939774 | -0.00231945 | 0.00356819 | 0.51581979 |
| 371 | rs9979383 | 0.00314003 | 0.00370347 | 0.39672083 |

SNPs: single-nucleotide polymorphisms; se: standard error

Supplementary Table 10：Leave-one-out analysis for estimate of EOS-IgG.

|  | SNPs | beta | se | p |
| --- | --- | --- | --- | --- |
| 1 | rs1004870 | 0.0194651 | 0.0086023 | 0.0236491 |
| 2 | rs10059018 | 0.0186933 | 0.0085988 | 0.0297100 |
| 3 | rs10062687 | 0.0194401 | 0.0085909 | 0.0236437 |
| 4 | rs10100356 | 0.0192365 | 0.0085803 | 0.0249661 |
| 5 | rs10165678 | 0.0189594 | 0.0086016 | 0.0275129 |
| 6 | rs10174238 | 0.0188445 | 0.0085978 | 0.0283944 |
| 7 | rs10195713 | 0.0190161 | 0.0086040 | 0.0270951 |
| 8 | rs1036332 | 0.0184594 | 0.0086109 | 0.0320551 |
| 9 | rs1037674 | 0.0189756 | 0.0086014 | 0.0273767 |
| 10 | rs1039341 | 0.0195007 | 0.0085911 | 0.0232163 |
| 11 | rs10472984 | 0.0182601 | 0.0086059 | 0.0338545 |
| 12 | rs1047891 | 0.0195186 | 0.0085649 | 0.0226722 |
| 13 | rs1057258 | 0.0192012 | 0.0086088 | 0.0257204 |
| 14 | rs1059091 | 0.0182985 | 0.0086100 | 0.0335660 |
| 15 | rs10745763 | 0.0190730 | 0.0085999 | 0.0265673 |
| 16 | rs10777378 | 0.0183992 | 0.0085929 | 0.0322578 |
| 17 | rs10782957 | 0.0187982 | 0.0086071 | 0.0289606 |
| 18 | rs10876550 | 0.0194374 | 0.0085665 | 0.0232680 |
| 19 | rs10900595 | 0.0193248 | 0.0085823 | 0.0243410 |
| 20 | rs10930337 | 0.0190474 | 0.0086011 | 0.0267923 |
| 21 | rs10962640 | 0.0191781 | 0.0085903 | 0.0255796 |
| 22 | rs1099448 | 0.0189070 | 0.0086051 | 0.0280077 |
| 23 | rs10995240 | 0.0180357 | 0.0086270 | 0.0365634 |
| 24 | rs11065822 | 0.0172151 | 0.0086697 | 0.0470696 |
| 25 | rs11071528 | 0.0188050 | 0.0085959 | 0.0286932 |
| 26 | rs11071559 | 0.0186862 | 0.0085943 | 0.0296852 |
| 27 | rs11079340 | 0.0188148 | 0.0085963 | 0.0286185 |
| 28 | rs11088236 | 0.0187442 | 0.0086007 | 0.0293041 |
| 29 | rs111759324 | 0.0181155 | 0.0085836 | 0.0348171 |
| 30 | rs112036266 | 0.0190550 | 0.0086006 | 0.0267233 |
| 31 | rs11204682 | 0.0200741 | 0.0085959 | 0.0195268 |
| 32 | rs11236813 | 0.0189461 | 0.0086062 | 0.0277045 |
| 33 | rs11255507 | 0.0192067 | 0.0085946 | 0.0254349 |
| 34 | rs113105190 | 0.0189837 | 0.0086045 | 0.0273663 |
| 35 | rs113542380 | 0.0183812 | 0.0085553 | 0.0316731 |
| 36 | rs11555542 | 0.0202669 | 0.0085830 | 0.0182116 |
| 37 | rs11571404 | 0.0195743 | 0.0085245 | 0.0216623 |
| 38 | rs11578794 | 0.0189729 | 0.0086019 | 0.0274071 |
| 39 | rs11628569 | 0.0185909 | 0.0085859 | 0.0303656 |
| 40 | rs11647946 | 0.0187490 | 0.0085936 | 0.0291286 |
| 41 | rs11684770 | 0.0188219 | 0.0085994 | 0.0286155 |
| 42 | rs11695281 | 0.0195630 | 0.0085889 | 0.0227436 |
| 43 | rs11701475 | 0.0191985 | 0.0085910 | 0.0254362 |
| 44 | rs11702918 | 0.0190251 | 0.0086022 | 0.0269899 |
| 45 | rs1170439 | 0.0195379 | 0.0085807 | 0.0227879 |
| 46 | rs117068593 | 0.0190242 | 0.0086070 | 0.0270832 |
| 47 | rs1178016 | 0.0191967 | 0.0085885 | 0.0254062 |
| 48 | rs11786536 | 0.0190561 | 0.0086055 | 0.0268004 |
| 49 | rs117955557 | 0.0189769 | 0.0086131 | 0.0275764 |
| 50 | rs118013485 | 0.0186065 | 0.0085918 | 0.0303404 |
| 51 | rs11869228 | 0.0190769 | 0.0086018 | 0.0265695 |
| 52 | rs11886928 | 0.0190418 | 0.0086011 | 0.0268365 |
| 53 | rs11931711 | 0.0184671 | 0.0085798 | 0.0313674 |
| 54 | rs12100034 | 0.0191924 | 0.0085980 | 0.0256022 |
| 55 | rs12154498 | 0.0200142 | 0.0085479 | 0.0192109 |
| 56 | rs121564 | 0.0187660 | 0.0085991 | 0.0290857 |
| 57 | rs12208103 | 0.0178968 | 0.0085811 | 0.0370155 |
| 58 | rs12361586 | 0.0187696 | 0.0085890 | 0.0288659 |
| 59 | rs12408934 | 0.0182262 | 0.0085858 | 0.0337681 |
| 60 | rs12470046 | 0.0185473 | 0.0085645 | 0.0303425 |
| 61 | rs12487980 | 0.0187830 | 0.0085946 | 0.0288576 |
| 62 | rs12530946 | 0.0181190 | 0.0086273 | 0.0357109 |
| 63 | rs12540285 | 0.0189334 | 0.0086013 | 0.0277209 |
| 64 | rs12545733 | 0.0197651 | 0.0085916 | 0.0214185 |
| 65 | rs1257192 | 0.0190084 | 0.0086004 | 0.0270920 |
| 66 | rs12581511 | 0.0189585 | 0.0086016 | 0.0275199 |
| 67 | rs12681644 | 0.0188275 | 0.0086018 | 0.0286113 |
| 68 | rs12705849 | 0.0191969 | 0.0086036 | 0.0256627 |
| 69 | rs12820863 | 0.0186644 | 0.0086037 | 0.0300558 |
| 70 | rs12878610 | 0.0189926 | 0.0086019 | 0.0272480 |
| 71 | rs12882281 | 0.0189526 | 0.0086011 | 0.0275585 |
| 72 | rs12941068 | 0.0191403 | 0.0085960 | 0.0259703 |
| 73 | rs1296535 | 0.0192994 | 0.0085900 | 0.0246568 |
| 74 | rs12978850 | 0.0185611 | 0.0085554 | 0.0300437 |
| 75 | rs13073683 | 0.0195428 | 0.0085589 | 0.0224114 |
| 76 | rs13105682 | 0.0189824 | 0.0086016 | 0.0273251 |
| 77 | rs13120371 | 0.0190990 | 0.0086004 | 0.0263709 |
| 78 | rs13138355 | 0.0188898 | 0.0086399 | 0.0287902 |
| 79 | rs13139941 | 0.0193001 | 0.0085728 | 0.0243655 |
| 80 | rs13207791 | 0.0187834 | 0.0085982 | 0.0289202 |
| 81 | rs13226583 | 0.0187968 | 0.0086330 | 0.0294572 |
| 82 | rs1323650 | 0.0188205 | 0.0085987 | 0.0286143 |
| 83 | rs13511 | 0.0183586 | 0.0085640 | 0.0320561 |
| 84 | rs1353286 | 0.0185767 | 0.0085965 | 0.0306979 |
| 85 | rs1365623 | 0.0188135 | 0.0085959 | 0.0286212 |
| 86 | rs1395269 | 0.0190628 | 0.0086084 | 0.0267977 |
| 87 | rs139640694 | 0.0188156 | 0.0086006 | 0.0286913 |
| 88 | rs1406449 | 0.0189349 | 0.0086013 | 0.0277083 |
| 89 | rs1414517 | 0.0192466 | 0.0086011 | 0.0252404 |
| 90 | rs1427499 | 0.0188345 | 0.0086032 | 0.0285795 |
| 91 | rs144569746 | 0.0173989 | 0.0085803 | 0.0425832 |
| 92 | rs1448187 | 0.0194436 | 0.0085708 | 0.0232942 |
| 93 | rs14713 | 0.0188812 | 0.0085992 | 0.0281138 |
| 94 | rs1471816 | 0.0186717 | 0.0085807 | 0.0295537 |
| 95 | rs1479918 | 0.0191265 | 0.0086012 | 0.0261680 |
| 96 | rs1519602 | 0.0184124 | 0.0085412 | 0.0311046 |
| 97 | rs1529745 | 0.0188469 | 0.0085991 | 0.0283986 |
| 98 | rs1539174 | 0.0189299 | 0.0086221 | 0.0281275 |
| 99 | rs1547258 | 0.0190513 | 0.0086052 | 0.0268330 |
| 100 | rs159963 | 0.0187779 | 0.0086037 | 0.0290709 |
| 101 | rs1672753 | 0.0188930 | 0.0086013 | 0.0280548 |
| 102 | rs1684578 | 0.0186242 | 0.0085905 | 0.0301579 |
| 103 | rs1689510 | 0.0193041 | 0.0086074 | 0.0249133 |
| 104 | rs16956811 | 0.0188278 | 0.0086012 | 0.0285983 |
| 105 | rs17061503 | 0.0189431 | 0.0086215 | 0.0280060 |
| 106 | rs17175830 | 0.0192663 | 0.0086117 | 0.0252709 |
| 107 | rs17293632 | 0.0191998 | 0.0086091 | 0.0257361 |
| 108 | rs174548 | 0.0187141 | 0.0086036 | 0.0296187 |
| 109 | rs17482472 | 0.0179422 | 0.0085267 | 0.0353576 |
| 110 | rs17516457 | 0.0175710 | 0.0086764 | 0.0428520 |
| 111 | rs175705 | 0.0190514 | 0.0086276 | 0.0272308 |
| 112 | rs17653687 | 0.0191123 | 0.0085974 | 0.0262136 |
| 113 | rs17668272 | 0.0182453 | 0.0085937 | 0.0337461 |
| 114 | rs17682575 | 0.0191021 | 0.0085993 | 0.0263274 |
| 115 | rs17689159 | 0.0186401 | 0.0085866 | 0.0299442 |
| 116 | rs17849501 | 0.0191487 | 0.0085929 | 0.0258510 |
| 117 | rs1800692 | 0.0195522 | 0.0085739 | 0.0225814 |
| 118 | rs180506 | 0.0185698 | 0.0085955 | 0.0307413 |
| 119 | rs1828803 | 0.0189253 | 0.0086017 | 0.0277940 |
| 120 | rs1861489 | 0.0190416 | 0.0085992 | 0.0268038 |
| 121 | rs201798 | 0.0194825 | 0.0085849 | 0.0232442 |
| 122 | rs2025489 | 0.0189846 | 0.0086034 | 0.0273383 |
| 123 | rs2089979 | 0.0186713 | 0.0085918 | 0.0297685 |
| 124 | rs214080 | 0.0192097 | 0.0085894 | 0.0253222 |
| 125 | rs2182885 | 0.0189810 | 0.0086132 | 0.0275451 |
| 126 | rs2223043 | 0.0194925 | 0.0085778 | 0.0230596 |
| 127 | rs2228467 | 0.0191355 | 0.0086178 | 0.0263877 |
| 128 | rs2239633 | 0.0191204 | 0.0086226 | 0.0265911 |
| 129 | rs2253427 | 0.0190440 | 0.0085986 | 0.0267758 |
| 130 | rs2399441 | 0.0186970 | 0.0086003 | 0.0297065 |
| 131 | rs2410732 | 0.0189197 | 0.0086012 | 0.0278317 |
| 132 | rs2419313 | 0.0189558 | 0.0086036 | 0.0275783 |
| 133 | rs2431097 | 0.0188108 | 0.0086003 | 0.0287260 |
| 134 | rs2497318 | 0.0182342 | 0.0086017 | 0.0340193 |
| 135 | rs2502995 | 0.0192915 | 0.0086018 | 0.0249147 |
| 136 | rs2505521 | 0.0185905 | 0.0085730 | 0.0301219 |
| 137 | rs2566133 | 0.0195056 | 0.0085585 | 0.0226608 |
| 138 | rs2579505 | 0.0176515 | 0.0086076 | 0.0402965 |
| 139 | rs2646438 | 0.0193940 | 0.0085941 | 0.0240297 |
| 140 | rs2713548 | 0.0186091 | 0.0085772 | 0.0300383 |
| 141 | rs2788211 | 0.0188379 | 0.0085994 | 0.0284800 |
| 142 | rs2807740 | 0.0194690 | 0.0086065 | 0.0236899 |
| 143 | rs2817377 | 0.0191132 | 0.0085954 | 0.0261716 |
| 144 | rs28362902 | 0.0190924 | 0.0085964 | 0.0263534 |
| 145 | rs2838317 | 0.0185008 | 0.0085657 | 0.0307832 |
| 146 | rs28383314 | 0.0233704 | 0.0085184 | 0.0060785 |
| 147 | rs28421324 | 0.0189464 | 0.0086100 | 0.0277700 |
| 148 | rs2847266 | 0.0191512 | 0.0085958 | 0.0258835 |
| 149 | rs2850542 | 0.0190588 | 0.0086001 | 0.0266829 |
| 150 | rs28532037 | 0.0192472 | 0.0085988 | 0.0251983 |
| 151 | rs2864936 | 0.0188957 | 0.0085999 | 0.0280056 |
| 152 | rs2887502 | 0.0186218 | 0.0085840 | 0.0300555 |
| 153 | rs2894401 | 0.0194806 | 0.0085819 | 0.0232102 |
| 154 | rs290430 | 0.0183563 | 0.0085206 | 0.0312145 |
| 155 | rs2920505 | 0.0189722 | 0.0086184 | 0.0277109 |
| 156 | rs295 | 0.0194979 | 0.0085548 | 0.0226565 |
| 157 | rs295273 | 0.0186231 | 0.0085955 | 0.0302640 |
| 158 | rs2979489 | 0.0188082 | 0.0085971 | 0.0286882 |
| 159 | rs2992333 | 0.0182724 | 0.0086128 | 0.0338758 |
| 160 | rs301162 | 0.0191259 | 0.0086046 | 0.0262331 |
| 161 | rs3024971 | 0.0189255 | 0.0086128 | 0.0279933 |
| 162 | rs3093023 | 0.0183899 | 0.0085396 | 0.0312815 |
| 163 | rs3096309 | 0.0187274 | 0.0085880 | 0.0292097 |
| 164 | rs3110791 | 0.0189634 | 0.0086033 | 0.0275096 |
| 165 | rs3218148 | 0.0185340 | 0.0085879 | 0.0309164 |
| 166 | rs33982662 | 0.0190133 | 0.0086050 | 0.0271360 |
| 167 | rs34212866 | 0.0193746 | 0.0085857 | 0.0240322 |
| 168 | rs34290285 | 0.0182924 | 0.0086283 | 0.0340024 |
| 169 | rs34363176 | 0.0187665 | 0.0085932 | 0.0289707 |
| 170 | rs34448954 | 0.0190502 | 0.0086019 | 0.0267846 |
| 171 | rs34466956 | 0.0192553 | 0.0085877 | 0.0249481 |
| 172 | rs34495 | 0.0190836 | 0.0086045 | 0.0265641 |
| 173 | rs34505104 | 0.0187330 | 0.0086079 | 0.0295362 |
| 174 | rs34631302 | 0.0187052 | 0.0085848 | 0.0293413 |
| 175 | rs346835 | 0.0186534 | 0.0086062 | 0.0302023 |
| 176 | rs350836 | 0.0190206 | 0.0086034 | 0.0270478 |
| 177 | rs35249183 | 0.0184869 | 0.0085982 | 0.0315490 |
| 178 | rs35409523 | 0.0194089 | 0.0086056 | 0.0241087 |
| 179 | rs36084354 | 0.0187634 | 0.0086145 | 0.0293972 |
| 180 | rs3731211 | 0.0193450 | 0.0085852 | 0.0242402 |
| 181 | rs3742704 | 0.0188516 | 0.0085986 | 0.0283506 |
| 182 | rs3746420 | 0.0191386 | 0.0085986 | 0.0260290 |
| 183 | rs3747869 | 0.0190348 | 0.0085991 | 0.0268577 |
| 184 | rs3757114 | 0.0186788 | 0.0086092 | 0.0300350 |
| 185 | rs3785356 | 0.0201373 | 0.0085641 | 0.0187048 |
| 186 | rs3786586 | 0.0194208 | 0.0085965 | 0.0238733 |
| 187 | rs3790163 | 0.0195814 | 0.0085299 | 0.0216975 |
| 188 | rs3804590 | 0.0188463 | 0.0086032 | 0.0284803 |
| 189 | rs3812206 | 0.0189040 | 0.0086004 | 0.0279457 |
| 190 | rs3823536 | 0.0193830 | 0.0085878 | 0.0240062 |
| 191 | rs3824867 | 0.0192353 | 0.0085933 | 0.0251952 |
| 192 | rs3846855 | 0.0180354 | 0.0085542 | 0.0349979 |
| 193 | rs3850107 | 0.0190532 | 0.0086002 | 0.0267304 |
| 194 | rs3950296 | 0.0186638 | 0.0086006 | 0.0300029 |
| 195 | rs397187 | 0.0187113 | 0.0085896 | 0.0293783 |
| 196 | rs4074672 | 0.0188396 | 0.0086008 | 0.0284916 |
| 197 | rs410867 | 0.0207790 | 0.0086134 | 0.0158469 |
| 198 | rs412884 | 0.0178704 | 0.0086579 | 0.0390135 |
| 199 | rs4142528 | 0.0190809 | 0.0087284 | 0.0288093 |
| 200 | rs4148757 | 0.0189910 | 0.0086027 | 0.0272752 |
| 201 | rs4240624 | 0.0187728 | 0.0085926 | 0.0289065 |
| 202 | rs4280242 | 0.0183657 | 0.0086059 | 0.0328361 |
| 203 | rs4310436 | 0.0190229 | 0.0086005 | 0.0269775 |
| 204 | rs4347868 | 0.0189311 | 0.0086009 | 0.0277321 |
| 205 | rs4409785 | 0.0189176 | 0.0086015 | 0.0278548 |
| 206 | rs460631 | 0.0191726 | 0.0086009 | 0.0258036 |
| 207 | rs4618204 | 0.0189721 | 0.0086058 | 0.0274848 |
| 208 | rs4652560 | 0.0190383 | 0.0086025 | 0.0268891 |
| 209 | rs4675190 | 0.0191212 | 0.0085953 | 0.0261081 |
| 210 | rs4680250 | 0.0186346 | 0.0085885 | 0.0300290 |
| 211 | rs4703589 | 0.0188400 | 0.0086006 | 0.0284850 |
| 212 | rs4703730 | 0.0194178 | 0.0085788 | 0.0236074 |
| 213 | rs4721559 | 0.0190044 | 0.0086032 | 0.0271749 |
| 214 | rs4722171 | 0.0189355 | 0.0086143 | 0.0279395 |
| 215 | rs473739 | 0.0190776 | 0.0085990 | 0.0265154 |
| 216 | rs4746153 | 0.0187020 | 0.0085879 | 0.0294267 |
| 217 | rs4849903 | 0.0191710 | 0.0086035 | 0.0258622 |
| 218 | rs4870977 | 0.0189553 | 0.0086017 | 0.0275467 |
| 219 | rs4908835 | 0.0189257 | 0.0086022 | 0.0277990 |
| 220 | rs4931002 | 0.0187847 | 0.0086030 | 0.0289978 |
| 221 | rs495149 | 0.0187099 | 0.0085999 | 0.0295850 |
| 222 | rs496475 | 0.0190868 | 0.0086254 | 0.0269082 |
| 223 | rs556063 | 0.0192782 | 0.0085824 | 0.0246885 |
| 224 | rs55868524 | 0.0183880 | 0.0085432 | 0.0313688 |
| 225 | rs55879743 | 0.0184853 | 0.0086218 | 0.0320304 |
| 226 | rs55977204 | 0.0190129 | 0.0086006 | 0.0270601 |
| 227 | rs56117721 | 0.0199791 | 0.0086499 | 0.0209015 |
| 228 | rs56179563 | 0.0195972 | 0.0085650 | 0.0221345 |
| 229 | rs56268488 | 0.0189868 | 0.0086009 | 0.0272762 |
| 230 | rs56330463 | 0.0195673 | 0.0086253 | 0.0232924 |
| 231 | rs574183 | 0.0188723 | 0.0086008 | 0.0282182 |
| 232 | rs5747308 | 0.0187158 | 0.0085968 | 0.0294766 |
| 233 | rs57633475 | 0.0190328 | 0.0086030 | 0.0269432 |
| 234 | rs57834782 | 0.0195589 | 0.0086472 | 0.0237047 |
| 235 | rs58745116 | 0.0192646 | 0.0085908 | 0.0249312 |
| 236 | rs58833930 | 0.0190151 | 0.0086023 | 0.0270722 |
| 237 | rs594479 | 0.0193082 | 0.0085700 | 0.0242589 |
| 238 | rs60175411 | 0.0184803 | 0.0085955 | 0.0315554 |
| 239 | rs60600003 | 0.0185891 | 0.0086104 | 0.0308572 |
| 240 | rs6080761 | 0.0185799 | 0.0085941 | 0.0306238 |
| 241 | rs6103572 | 0.0188529 | 0.0086072 | 0.0284985 |
| 242 | rs6139104 | 0.0191195 | 0.0085938 | 0.0260951 |
| 243 | rs6141755 | 0.0188354 | 0.0086008 | 0.0285276 |
| 244 | rs61426394 | 0.0194712 | 0.0085596 | 0.0229187 |
| 245 | rs61798836 | 0.0191659 | 0.0085887 | 0.0256471 |
| 246 | rs62011287 | 0.0190147 | 0.0086008 | 0.0270491 |
| 247 | rs62061733 | 0.0191861 | 0.0086143 | 0.0259312 |
| 248 | rs62086903 | 0.0195350 | 0.0085990 | 0.0231000 |
| 249 | rs62105489 | 0.0194555 | 0.0085606 | 0.0230453 |
| 250 | rs62183994 | 0.0190738 | 0.0085984 | 0.0265344 |
| 251 | rs62308111 | 0.0187205 | 0.0085932 | 0.0293670 |
| 252 | rs62385501 | 0.0189630 | 0.0086021 | 0.0274919 |
| 253 | rs62408224 | 0.0192924 | 0.0086346 | 0.0254633 |
| 254 | rs62420764 | 0.0187411 | 0.0085945 | 0.0292126 |
| 255 | rs62473720 | 0.0189165 | 0.0086018 | 0.0278679 |
| 256 | rs62539154 | 0.0185260 | 0.0085828 | 0.0308891 |
| 257 | rs634534 | 0.0187439 | 0.0086218 | 0.0297035 |
| 258 | rs637064 | 0.0188286 | 0.0086106 | 0.0287664 |
| 259 | rs6479336 | 0.0198218 | 0.0085659 | 0.0206657 |
| 260 | rs6496717 | 0.0194296 | 0.0085782 | 0.0235134 |
| 261 | rs6540985 | 0.0191789 | 0.0086013 | 0.0257625 |
| 262 | rs6556313 | 0.0187453 | 0.0086019 | 0.0293162 |
| 263 | rs6573020 | 0.0181324 | 0.0085580 | 0.0341103 |
| 264 | rs6684992 | 0.0184878 | 0.0086024 | 0.0316238 |
| 265 | rs6691839 | 0.0189835 | 0.0086009 | 0.0273030 |
| 266 | rs6731125 | 0.0193570 | 0.0085889 | 0.0242138 |
| 267 | rs6750754 | 0.0184253 | 0.0086702 | 0.0335763 |
| 268 | rs67856193 | 0.0194022 | 0.0085977 | 0.0240290 |
| 269 | rs6787336 | 0.0193985 | 0.0086169 | 0.0243725 |
| 270 | rs6904506 | 0.0186205 | 0.0086141 | 0.0306456 |
| 271 | rs6924350 | 0.0186131 | 0.0086328 | 0.0310773 |
| 272 | rs6924387 | 0.0184743 | 0.0085799 | 0.0313034 |
| 273 | rs6930635 | 0.0181378 | 0.0085693 | 0.0342943 |
| 274 | rs6956283 | 0.0187880 | 0.0085990 | 0.0288959 |
| 275 | rs6971710 | 0.0187927 | 0.0086084 | 0.0290307 |
| 276 | rs6979947 | 0.0190323 | 0.0085999 | 0.0268917 |
| 277 | rs6986109 | 0.0190908 | 0.0086012 | 0.0264497 |
| 278 | rs6989099 | 0.0189096 | 0.0086048 | 0.0279801 |
| 279 | rs699664 | 0.0187608 | 0.0085973 | 0.0290972 |
| 280 | rs6999452 | 0.0188354 | 0.0085979 | 0.0284745 |
| 281 | rs7026022 | 0.0189549 | 0.0086013 | 0.0275441 |
| 282 | rs708776 | 0.0186343 | 0.0085851 | 0.0299657 |
| 283 | rs7123726 | 0.0191024 | 0.0085997 | 0.0263316 |
| 284 | rs7141943 | 0.0194145 | 0.0085761 | 0.0235872 |
| 285 | rs71429414 | 0.0188273 | 0.0086012 | 0.0286025 |
| 286 | rs7158239 | 0.0183186 | 0.0085523 | 0.0321973 |
| 287 | rs71628184 | 0.0191012 | 0.0085977 | 0.0263053 |
| 288 | rs7173571 | 0.0186974 | 0.0085934 | 0.0295718 |
| 289 | rs7215391 | 0.0188649 | 0.0085987 | 0.0282402 |
| 290 | rs725613 | 0.0206001 | 0.0086064 | 0.0166853 |
| 291 | rs7257 | 0.0187516 | 0.0086217 | 0.0296346 |
| 292 | rs72766638 | 0.0189886 | 0.0086044 | 0.0273245 |
| 293 | rs72844043 | 0.0189577 | 0.0086010 | 0.0275156 |
| 294 | rs72987040 | 0.0189387 | 0.0086024 | 0.0276968 |
| 295 | rs72998585 | 0.0187188 | 0.0086895 | 0.0312256 |
| 296 | rs73049239 | 0.0188757 | 0.0086022 | 0.0282140 |
| 297 | rs73118830 | 0.0187400 | 0.0085988 | 0.0293039 |
| 298 | rs73176183 | 0.0189639 | 0.0086020 | 0.0274826 |
| 299 | rs73176685 | 0.0190807 | 0.0086062 | 0.0266169 |
| 300 | rs73187852 | 0.0190486 | 0.0086036 | 0.0268275 |
| 301 | rs73203442 | 0.0179684 | 0.0086848 | 0.0385518 |
| 302 | rs73232881 | 0.0181508 | 0.0086716 | 0.0363364 |
| 303 | rs73238201 | 0.0187058 | 0.0085888 | 0.0294118 |
| 304 | rs73272842 | 0.0193910 | 0.0085850 | 0.0239017 |
| 305 | rs7327960 | 0.0189579 | 0.0086027 | 0.0275438 |
| 306 | rs73322872 | 0.0193759 | 0.0085668 | 0.0237133 |
| 307 | rs73428834 | 0.0191872 | 0.0085963 | 0.0256122 |
| 308 | rs7354779 | 0.0189578 | 0.0086036 | 0.0275612 |
| 309 | rs7382061 | 0.0183849 | 0.0086641 | 0.0338410 |
| 310 | rs73963711 | 0.0187770 | 0.0086032 | 0.0290675 |
| 311 | rs7423615 | 0.0194731 | 0.0085788 | 0.0232127 |
| 312 | rs74299961 | 0.0191089 | 0.0085946 | 0.0261906 |
| 313 | rs743002 | 0.0182296 | 0.0085985 | 0.0339980 |
| 314 | rs74331768 | 0.0188245 | 0.0085986 | 0.0285791 |
| 315 | rs7441808 | 0.0190753 | 0.0085997 | 0.0265457 |
| 316 | rs746550 | 0.0191913 | 0.0086024 | 0.0256867 |
| 317 | rs7569084 | 0.0185139 | 0.0085904 | 0.0311469 |
| 318 | rs7636495 | 0.0188987 | 0.0086048 | 0.0280711 |
| 319 | rs7646283 | 0.0183520 | 0.0085953 | 0.0327514 |
| 320 | rs7646695 | 0.0192238 | 0.0085884 | 0.0251981 |
| 321 | rs76474320 | 0.0191209 | 0.0085994 | 0.0261814 |
| 322 | rs7649812 | 0.0189951 | 0.0086023 | 0.0272342 |
| 323 | rs76793172 | 0.0186111 | 0.0086035 | 0.0305247 |
| 324 | rs76830965 | 0.0190570 | 0.0086011 | 0.0267161 |
| 325 | rs7687708 | 0.0192273 | 0.0085873 | 0.0251528 |
| 326 | rs76981581 | 0.0188143 | 0.0085968 | 0.0286316 |
| 327 | rs7700687 | 0.0197837 | 0.0086183 | 0.0217016 |
| 328 | rs778756 | 0.0188308 | 0.0086021 | 0.0285893 |
| 329 | rs7797428 | 0.0187703 | 0.0085957 | 0.0289842 |
| 330 | rs783646 | 0.0194631 | 0.0085712 | 0.0231611 |
| 331 | rs7839946 | 0.0190918 | 0.0085961 | 0.0263523 |
| 332 | rs7840212 | 0.0189543 | 0.0086312 | 0.0280907 |
| 333 | rs7846314 | 0.0188021 | 0.0086116 | 0.0290094 |
| 334 | rs7868130 | 0.0197584 | 0.0085907 | 0.0214495 |
| 335 | rs7897422 | 0.0192944 | 0.0085906 | 0.0247052 |
| 336 | rs79716587 | 0.0193214 | 0.0085903 | 0.0244995 |
| 337 | rs7975 | 0.0186987 | 0.0085843 | 0.0293875 |
| 338 | rs7986796 | 0.0198322 | 0.0085689 | 0.0206437 |
| 339 | rs79881201 | 0.0186846 | 0.0086329 | 0.0304383 |
| 340 | rs8 | 0.0187716 | 0.0086016 | 0.0290846 |
| 341 | rs80066203 | 0.0193680 | 0.0085644 | 0.0237309 |
| 342 | rs8012643 | 0.0185982 | 0.0085839 | 0.0302631 |
| 343 | rs8020739 | 0.0190891 | 0.0086095 | 0.0266090 |
| 344 | rs8026803 | 0.0186574 | 0.0086128 | 0.0302927 |
| 345 | rs8044920 | 0.0191794 | 0.0085901 | 0.0255664 |
| 346 | rs8061729 | 0.0192653 | 0.0085815 | 0.0247687 |
| 347 | rs8076052 | 0.0187512 | 0.0086005 | 0.0292386 |
| 348 | rs8108623 | 0.0191647 | 0.0086006 | 0.0258595 |
| 349 | rs8142080 | 0.0190614 | 0.0086020 | 0.0266956 |
| 350 | rs884634 | 0.0190142 | 0.0086008 | 0.0270526 |
| 351 | rs911603 | 0.0187618 | 0.0086099 | 0.0293235 |
| 352 | rs925966 | 0.0187363 | 0.0085988 | 0.0293367 |
| 353 | rs9349322 | 0.0188796 | 0.0086036 | 0.0282085 |
| 354 | rs9389268 | 0.0195056 | 0.0086274 | 0.0237670 |
| 355 | rs9392525 | 0.0184696 | 0.0085607 | 0.0309679 |
| 356 | rs941616 | 0.0186791 | 0.0085839 | 0.0295512 |
| 357 | rs954954 | 0.0190009 | 0.0086083 | 0.0272954 |
| 358 | rs962993 | 0.0179324 | 0.0086177 | 0.0374452 |
| 359 | rs964184 | 0.0188456 | 0.0086049 | 0.0285180 |
| 360 | rs9666598 | 0.0190133 | 0.0086028 | 0.0270960 |
| 361 | rs9675999 | 0.0187403 | 0.0085988 | 0.0293019 |
| 362 | rs9815874 | 0.0190667 | 0.0086127 | 0.0268437 |
| 363 | rs9818987 | 0.0192550 | 0.0085850 | 0.0249062 |
| 364 | rs9835307 | 0.0189641 | 0.0086132 | 0.0276825 |
| 365 | rs9837045 | 0.0190128 | 0.0086001 | 0.0270518 |
| 366 | rs9840310 | 0.0188497 | 0.0085983 | 0.0283604 |
| 367 | rs9880192 | 0.0198967 | 0.0086194 | 0.0209788 |
| 368 | rs9889262 | 0.0196071 | 0.0086006 | 0.0226236 |
| 369 | rs9894839 | 0.0190329 | 0.0085999 | 0.0268867 |
| 370 | rs9939774 | 0.0186843 | 0.0086148 | 0.0300923 |
| 371 | rs9979383 | 0.0185956 | 0.0086097 | 0.0307851 |
| 372 | All | 0.0189556 | 0.0085864 | 0.0272706 |

SNPs: single-nucleotide polymorphisms; se: standard error

Supplementary Table 11: Single SNP analysis for estimate of IgG-Asthma.

|  | SNP | beta | se | p |
| --- | --- | --- | --- | --- |
| 1 | rs115032978 | -0.0685355 | 27403320 | 0.2482610 |
| 2 | rs11642879 | -0.00523074 | 6734877 | 0.8302160 |
| 3 | rs12463441 | 0.0302612 | 227707454 | 0.1351880 |
| 4 | rs1983814 | -0.018969 | 116382815 | 0.4444440 |
| 5 | rs2257609 | 0.00732037 | 18961868 | 0.7180490 |
| 6 | rs2780076 | 0.00444375 | 9355243 | 0.8375260 |
| 7 | rs34740816 | 0.00896722 | 160247432 | 0.7906040 |
| 8 | rs35755728 | -0.0191093 | 61777902 | 0.4957470 |
| 9 | rs4655368 | 0.0257103 | 214755775 | 0.2388970 |
| 10 | rs58371504 | -0.0221189 | 142513030 | 0.7287240 |
| 11 | rs7256714 | -0.0368502 | 48825618 | 0.0917107 |
| 12 | rs75832668 | 0.0597627 | 93618739 | 0.0902070 |
| 13 | rs9520848 | -0.00692605 | 108343101 | 0.7809330 |

SNPs: single-nucleotide polymorphisms; se: standard error

Supplementary Table 12：Leave-one-out analysis for estimate of IgG-Asthma.

|  | SNP | beta | se | p |
| --- | --- | --- | --- | --- |
| 1 | rs115032978 | 0.6555766 | 0.3725123 | 0.0784283 |
| 2 | rs11642879 | 0.7997099 | 0.3782010 | 0.0344717 |
| 3 | rs12463441 | 0.6109462 | 0.3791733 | 0.1071234 |
| 4 | rs1983814 | 0.8891832 | 0.3823335 | 0.0200359 |
| 5 | rs2257609 | 0.8242663 | 0.3797355 | 0.0299590 |
| 6 | rs2780076 | 0.7514255 | 0.3774736 | 0.0465171 |
| 7 | rs34740816 | 0.7330019 | 0.3733597 | 0.0496161 |
| 8 | rs35755728 | 0.7076571 | 0.3821917 | 0.0640866 |
| 9 | rs4655368 | 0.6459795 | 0.3810397 | 0.0900171 |
| 10 | rs58371504 | 0.7222064 | 0.3718348 | 0.0521035 |
| 11 | rs7256714 | 0.5831989 | 0.3817000 | 0.1265377 |
| 12 | rs75832668 | 0.5871383 | 0.3796094 | 0.1219379 |
| 13 | rs9520848 | 0.8012227 | 0.3771589 | 0.0336398 |
| 14 | All | 0.7162654 | 0.3632961 | 0.0486582 |

SNPs: single-nucleotide polymorphisms; se: standard error

Supplementary Table 13: Single SNP analysis for estimate of EOS-Asthma.

|  | SNP | beta | se | p |
| --- | --- | --- | --- | --- |
| 1 | rs1004870 | 0.0436179 | 0.0207078 | 0.0351739 |
| 2 | rs10059018 | -0.0493892 | 0.02758 | 0.0733314 |
| 3 | rs10062687 | 0.039574 | 0.0248009 | 0.1105620 |
| 4 | rs10100356 | 0.00513414 | 0.0235511 | 0.8274290 |
| 5 | rs10165678 | -0.0449974 | 0.0217934 | 0.0389493 |
| 6 | rs10174238 | 0.00791669 | 0.0238046 | 0.7394590 |
| 7 | rs10195713 | 0.0566165 | 0.032598 | 0.0824214 |
| 8 | rs1036332 | 0.0213272 | 0.0225927 | 0.3451760 |
| 9 | rs1037674 | -0.0306495 | 0.0226752 | 0.1764790 |
| 10 | rs1039341 | -0.00021499 | 0.020683 | 0.9917060 |
| 11 | rs10472984 | -0.0626047 | 0.0206765 | 0.0024632 |
| 12 | rs1047891 | -0.00549387 | 0.0215771 | 0.7990200 |
| 13 | rs1057258 | -0.0929363 | 0.0296918 | 0.0017478 |
| 14 | rs1059091 | 0.0268214 | 0.0216444 | 0.2152760 |
| 15 | rs10745763 | 0.00535244 | 0.020439 | 0.7934200 |
| 16 | rs10777378 | -0.0174725 | 0.0202696 | 0.3886840 |
| 17 | rs10782957 | 0.00641129 | 0.0209979 | 0.7601140 |
| 18 | rs10876550 | -0.0246737 | 0.0216943 | 0.2553970 |
| 19 | rs10900595 | -0.0113389 | 0.0254299 | 0.6556770 |
| 20 | rs10930337 | -0.0504015 | 0.0216804 | 0.0200854 |
| 21 | rs1099448 | 0.0373189 | 0.0203821 | 0.0671058 |
| 22 | rs10995240 | -0.059382 | 0.0216695 | 0.0061374 |
| 23 | rs11065822 | -0.00230947 | 0.0224845 | 0.9181900 |
| 24 | rs11071528 | 0.0026302 | 0.0279486 | 0.9250230 |
| 25 | rs11079340 | -0.00526491 | 0.0205206 | 0.7975130 |
| 26 | rs11088236 | 0.00679642 | 0.0202 | 0.7365280 |
| 27 | rs111759324 | -0.00871358 | 0.0394026 | 0.8249820 |
| 28 | rs112036266 | 0.0603787 | 0.0397838 | 0.1290980 |
| 29 | rs11204682 | 0.00819053 | 0.0252515 | 0.7456670 |
| 30 | rs11228990 | 0.0429709 | 0.0485669 | 0.3762770 |
| 31 | rs11236813 | -0.147806 | 0.0320191 | 3.9086E-06 |
| 32 | rs11255507 | 0.0458902 | 0.0242792 | 0.0587449 |
| 33 | rs113473633 | 0.169209 | 0.087542 | 0.0532500 |
| 34 | rs113496608 | -0.122421 | 0.0786306 | 0.1194920 |
| 35 | rs113542380 | 0.0248511 | 0.0560313 | 0.6573880 |
| 36 | rs113859409 | 0.0266341 | 0.0455714 | 0.5589190 |
| 37 | rs114152720 | -0.0409025 | 0.0521916 | 0.4332170 |
| 38 | rs114741563 | 0.0763945 | 0.169042 | 0.6513220 |
| 39 | rs11555542 | -0.0336889 | 0.0786313 | 0.6683300 |
| 40 | rs115647629 | 0.0286689 | 0.072282 | 0.6916430 |
| 41 | rs11571404 | 0.0397236 | 0.0254166 | 0.1180770 |
| 42 | rs11578794 | -0.0292518 | 0.0223479 | 0.1905570 |
| 43 | rs11628569 | 0.0205193 | 0.0210514 | 0.3296980 |
| 44 | rs11647946 | 0.00913043 | 0.0218208 | 0.6756350 |
| 45 | rs11684770 | -0.0198457 | 0.022618 | 0.3802540 |
| 46 | rs116888884 | -0.139935 | 0.163568 | 0.3922660 |
| 47 | rs11695281 | -0.0496684 | 0.0201942 | 0.013911 |
| 48 | rs11701475 | -0.0206418 | 0.0232825 | 0.375305 |
| 49 | rs11702918 | 0.0121113 | 0.0346732 | 0.726865 |
| 50 | rs1170439 | 0.0250221 | 0.0265472 | 0.345911 |
| 51 | rs117068593 | -0.0582259 | 0.0289403 | 0.044227 |
| 52 | rs117481629 | 0.0362552 | 0.069769 | 0.603311 |
| 53 | rs1178016 | -0.0136127 | 0.0202351 | 0.501121 |
| 54 | rs11786536 | 0.00269584 | 0.0262747 | 0.918279 |
| 55 | rs117955557 | 0.00194051 | 0.0221228 | 0.930103 |
| 56 | rs117961539 | -0.0118156 | 0.0593271 | 0.842138 |
| 57 | rs118013485 | -0.13793 | 0.0348599 | 7.5994E-05 |
| 58 | rs11869228 | 0.0414437 | 0.0277469 | 0.135271 |
| 59 | rs11886928 | 0.0256621 | 0.0242201 | 0.289356 |
| 60 | rs11931711 | 0.00500146 | 0.0213226 | 0.814549 |
| 61 | rs12100034 | 0.0137627 | 0.0214379 | 0.520885 |
| 62 | rs12154498 | -0.0039836 | 0.0260613 | 0.878512 |
| 63 | rs121564 | -0.0673054 | 0.0238231 | 0.004724 |
| 64 | rs12208103 | -0.0307855 | 0.0207075 | 0.137099 |
| 65 | rs12361586 | -0.0148134 | 0.0296968 | 0.617905 |
| 66 | rs12408934 | 0.00605829 | 0.031637 | 0.848139 |
| 67 | rs12470046 | 0.0069836 | 0.0216542 | 0.747069 |
| 68 | rs12487980 | -0.00348019 | 0.0226641 | 0.877960 |
| 69 | rs12530946 | -0.0192718 | 0.0215734 | 0.371689 |
| 70 | rs12540285 | 0.0100318 | 0.0217459 | 0.644568 |
| 71 | rs12545733 | -0.00834855 | 0.0219007 | 0.703055 |
| 72 | rs1257192 | 0.0107629 | 0.0275843 | 0.696400 |
| 73 | rs12581511 | -0.0204902 | 0.0253282 | 0.418524 |
| 74 | rs12705849 | 0.0391038 | 0.0210575 | 0.063311 |
| 75 | rs12820863 | 0.00521075 | 0.0211645 | 0.805526 |
| 76 | rs12861824 | -0.0260562 | 0.0228178 | 0.253485 |
| 77 | rs12878610 | 0.0137732 | 0.0202356 | 0.496099 |
| 78 | rs12882281 | -0.00103455 | 0.0250695 | 0.967083 |
| 79 | rs12928503 | 0.352319 | 0.147917 | 0.017225 |
| 80 | rs12941068 | -0.0609731 | 0.024706 | 0.013589 |
| 81 | rs12978850 | -0.0479156 | 0.0270711 | 0.076729 |
| 82 | rs13073683 | -0.0107669 | 0.0202062 | 0.594136 |
| 83 | rs13105682 | 0.234535 | 0.106132 | 0.027116 |
| 84 | rs13138355 | -0.023551 | 0.0237342 | 0.321061 |
| 85 | rs13139941 | -0.0128284 | 0.0248321 | 0.605431 |
| 86 | rs13207791 | -0.00244086 | 0.0657523 | 0.970388 |
| 87 | rs13226583 | -0.00256207 | 0.0299856 | 0.931909 |
| 88 | rs1323650 | 0.00981856 | 0.0221274 | 0.657239 |
| 89 | rs13251643 | 0.0351786 | 0.0459845 | 0.444266 |
| 90 | rs13313564 | -0.0528547 | 0.0504283 | 0.294585 |
| 91 | rs13511 | -0.0443177 | 0.0203023 | 0.029043 |
| 92 | rs1353286 | 0.00901651 | 0.0202932 | 0.656816 |
| 93 | rs1365623 | 0.00392259 | 0.0209933 | 0.851778 |
| 94 | rs137906075 | 0.0222904 | 0.0532674 | 0.675610 |
| 95 | rs1395269 | 0.0208282 | 0.0213557 | 0.329412 |
| 96 | rs139640694 | 0.0676961 | 0.0346058 | 0.050440 |
| 97 | rs1406449 | 0.0165439 | 0.0201791 | 0.412300 |
| 98 | rs1414517 | -0.0284949 | 0.0272631 | 0.295940 |
| 99 | rs1427499 | 0.00812615 | 0.0219222 | 0.710875 |
| 100 | rs143491704 | 0.0573329 | 0.0812833 | 0.480595 |
| 101 | rs144569746 | 0.0262268 | 0.033069 | 0.427723 |
| 102 | rs1448187 | 0.0178194 | 0.0236313 | 0.450814 |
| 103 | rs145947882 | 0.107776 | 0.0602985 | 0.073877 |
| 104 | rs146078144 | -0.117638 | 0.199239 | 0.554898 |
| 105 | rs146730870 | 0.122487 | 0.0555246 | 0.027384 |
| 106 | rs14713 | -0.0368753 | 0.024074 | 0.125585 |
| 107 | rs1471816 | -0.00829495 | 0.0201739 | 0.680945 |
| 108 | rs1479918 | -0.0573008 | 0.0291978 | 0.049703 |
| 109 | rs148219449 | 0.160311 | 0.146882 | 0.275083 |
| 110 | rs149110519 | -0.0344579 | 0.0642076 | 0.591499 |
| 111 | rs150640087 | -0.118093 | 0.08013 | 0.140545 |
| 112 | rs1516527 | -0.0390167 | 0.0612006 | 0.523785 |
| 113 | rs1519602 | 0.0383536 | 0.0214495 | 0.073761 |
| 114 | rs1529745 | -0.00611112 | 0.0310854 | 0.844147 |
| 115 | rs1539174 | 0.0229869 | 0.0250356 | 0.358530 |
| 116 | rs1547258 | -0.00875477 | 0.0256015 | 0.732379 |
| 117 | rs159963 | 0.0099421 | 0.021408 | 0.642355 |
| 118 | rs1672753 | -0.0351522 | 0.0245665 | 0.152460 |
| 119 | rs1684578 | 0.0281348 | 0.0210614 | 0.181598 |
| 120 | rs1689510 | 0.0378078 | 0.0216804 | 0.081182 |
| 121 | rs16903574 | 0.0710791 | 0.0302563 | 0.018811 |
| 122 | rs16956811 | -0.0280228 | 0.0466175 | 0.547758 |
| 123 | rs17061503 | 0.0361883 | 0.0210229 | 0.085182 |
| 124 | rs17175830 | 0.0237118 | 0.0239588 | 0.322324 |
| 125 | rs174548 | -0.00352236 | 0.0206696 | 0.864686 |
| 126 | rs17482472 | -0.00186624 | 0.0387568 | 0.961595 |
| 127 | rs17516457 | -0.0832546 | 0.0226564 | 0.000238 |
| 128 | rs175705 | 0.0435789 | 0.0206741 | 0.035039 |
| 129 | rs17653687 | -0.0278557 | 0.02912 | 0.338778 |
| 130 | rs17668272 | -0.0962382 | 0.0393628 | 0.014489 |
| 131 | rs17682575 | 0.0652278 | 0.0273644 | 0.017140 |
| 132 | rs17689159 | -0.0227811 | 0.0218997 | 0.298226 |
| 133 | rs17758695 | 0.0219442 | 0.0495757 | 0.658026 |
| 134 | rs17849501 | 0.00990933 | 0.0521916 | 0.849415 |
| 135 | rs1800692 | -0.0025143 | 0.0208972 | 0.904232 |
| 136 | rs180506 | 0.0148133 | 0.0263171 | 0.573518 |
| 137 | rs1828803 | 0.0637662 | 0.0211531 | 0.002573 |
| 138 | rs1861489 | 0.00029202 | 0.0236638 | 0.990154 |
| 139 | rs201798 | 0.0239458 | 0.0217289 | 0.270451 |
| 140 | rs2025489 | 0.0321184 | 0.0202067 | 0.111950 |
| 141 | rs2089979 | -0.0284663 | 0.0203572 | 0.162012 |
| 142 | rs2182885 | 0.0587219 | 0.0202359 | 0.0037093 |
| 143 | rs2223043 | 0.0187296 | 0.0210462 | 0.373505 |
| 144 | rs2228467 | -0.0553929 | 0.0378668 | 0.143512 |
| 145 | rs2239633 | 0.0158113 | 0.0202479 | 0.434871 |
| 146 | rs2253427 | 0.018112 | 0.0256157 | 0.479525 |
| 147 | rs2399441 | -0.036541 | 0.0218473 | 0.094414 |
| 148 | rs2410732 | 0.0113251 | 0.0218786 | 0.604713 |
| 149 | rs2419313 | 0.0287735 | 0.029305 | 0.326167 |
| 150 | rs2431097 | 0.0148165 | 0.0202378 | 0.464095 |
| 151 | rs2497318 | -0.0549622 | 0.0202786 | 0.006721 |
| 152 | rs2502995 | 0.0720029 | 0.0204302 | 0.000424 |
| 153 | rs2505521 | 0.0127698 | 0.024178 | 0.597391 |
| 154 | rs2566133 | -0.0437555 | 0.0202857 | 0.031009 |
| 155 | rs2579505 | -0.00952288 | 0.0212525 | 0.654094 |
| 156 | rs2646438 | -0.0203831 | 0.0204311 | 0.318448 |
| 157 | rs2713548 | 0.0204726 | 0.0207616 | 0.324093 |
| 158 | rs2788211 | -0.010263 | 0.0267049 | 0.700747 |
| 159 | rs2807740 | 0.0046555 | 0.0254659 | 0.854944 |
| 160 | rs2817377 | -0.00561082 | 0.0205359 | 0.784684 |
| 161 | rs28362902 | 0.0120917 | 0.0299842 | 0.686751 |
| 162 | rs2838317 | -0.0495318 | 0.0214405 | 0.020877 |
| 163 | rs28421324 | -0.0550273 | 0.0306407 | 0.072512 |
| 164 | rs2847266 | 0.0100173 | 0.0220714 | 0.649931 |
| 165 | rs2850542 | -0.0751686 | 0.0200955 | 0.000183 |
| 166 | rs28532037 | 0.00074616 | 0.0317922 | 0.981275 |
| 167 | rs2864936 | 0.0110243 | 0.0294055 | 0.707729 |
| 168 | rs2887502 | -0.0117495 | 0.0205565 | 0.567611 |
| 169 | rs2894401 | 0.00018102 | 0.0244041 | 0.994082 |
| 170 | rs290430 | 0.0319928 | 0.0208416 | 0.124773 |
| 171 | rs2920505 | -0.0478322 | 0.0202961 | 0.018437 |
| 172 | rs295 | -0.0400728 | 0.0240255 | 0.095330 |
| 173 | rs295273 | -0.0244998 | 0.0231588 | 0.290099 |
| 174 | rs2979489 | 0.0120949 | 0.0222321 | 0.586422 |
| 175 | rs2992333 | 0.00529992 | 0.0217292 | 0.807303 |
| 176 | rs301162 | 0.0306415 | 0.0312012 | 0.326070 |
| 177 | rs3024971 | -0.132674 | 0.0510178 | 0.009307 |
| 178 | rs3093023 | 0.00913231 | 0.0203826 | 0.654122 |
| 179 | rs3096309 | 0.0435762 | 0.0304909 | 0.152960 |
| 180 | rs3110791 | -0.00923349 | 0.0218504 | 0.672603 |
| 181 | rs3218148 | 0.019277 | 0.0212769 | 0.364932 |
| 182 | rs33982662 | 0.00056058 | 0.0232085 | 0.980730 |
| 183 | rs34173062 | 0.0779478 | 0.0449793 | 0.083101 |
| 184 | rs34210653 | -0.0682742 | 0.121435 | 0.573959 |
| 185 | rs34212866 | -0.00607441 | 0.0287417 | 0.832618 |
| 186 | rs34363176 | 0.0425728 | 0.0262146 | 0.104373 |
| 187 | rs34439695 | -0.0242574 | 0.0630045 | 0.700229 |
| 188 | rs34448954 | -0.05085 | 0.032636 | 0.119211 |
| 189 | rs34466956 | -0.021356 | 0.0203751 | 0.294574 |
| 190 | rs34495 | 0.0242627 | 0.0271045 | 0.370705 |
| 191 | rs34505104 | 0.00586982 | 0.0225716 | 0.794823 |
| 192 | rs34631302 | 0.0194286 | 0.0205772 | 0.345077 |
| 193 | rs350836 | -0.00083551 | 0.0230114 | 0.971036 |
| 194 | rs35249183 | 0.0416901 | 0.0470448 | 0.375522 |
| 195 | rs35409523 | -0.033851 | 0.0341868 | 0.322088 |
| 196 | rs36084354 | -0.0195791 | 0.0362297 | 0.588910 |
| 197 | rs3731211 | 0.0361872 | 0.023964 | 0.131028 |
| 198 | rs3742704 | 0.0209742 | 0.0369164 | 0.569931 |
| 199 | rs3746420 | -0.0452017 | 0.045908 | 0.324813 |
| 200 | rs3747869 | -0.00129514 | 0.0258397 | 0.960025 |
| 201 | rs3757114 | 0.00338094 | 0.0205139 | 0.869092 |
| 202 | rs3785356 | 0.0683328 | 0.0226164 | 0.002516 |
| 203 | rs3786586 | 0.00381666 | 0.0253598 | 0.880370 |
| 204 | rs3790163 | 0.00197439 | 0.0268502 | 0.941382 |
| 205 | rs3804590 | 0.0182787 | 0.0219639 | 0.405286 |
| 206 | rs3812206 | -0.036202 | 0.0213781 | 0.090377 |
| 207 | rs3823536 | -0.0109416 | 0.0203525 | 0.590851 |
| 208 | rs3824867 | -0.0128755 | 0.0206442 | 0.532833 |
| 209 | rs3846855 | -0.028448 | 0.0217064 | 0.189998 |
| 210 | rs3850107 | -0.0143571 | 0.0226249 | 0.525707 |
| 211 | rs3950296 | 0.0173724 | 0.0227185 | 0.444460 |
| 212 | rs397187 | -0.0240535 | 0.0210631 | 0.253464 |
| 213 | rs4074672 | 0.0105492 | 0.0221843 | 0.634414 |
| 214 | rs410867 | -0.0530459 | 0.0241679 | 0.028170 |
| 215 | rs412884 | 0.0271157 | 0.0217847 | 0.213237 |
| 216 | rs41313381 | 0.0767538 | 0.0791083 | 0.331929 |
| 217 | rs4142528 | -0.0914047 | 0.0221701 | 3.7415E-05 |
| 218 | rs4148757 | -0.0458749 | 0.0298137 | 0.123873 |
| 219 | rs4149909 | -0.0848581 | 0.0568448 | 0.135489 |
| 220 | rs4236746 | 0.0356426 | 0.0677896 | 0.599039 |
| 221 | rs4240624 | -0.0334638 | 0.0287078 | 0.243748 |
| 222 | rs4280242 | 0.0068911 | 0.0232816 | 0.767238 |
| 223 | rs4310436 | 0.020105 | 0.0291447 | 0.490299 |
| 224 | rs4347868 | -0.0151319 | 0.026462 | 0.567434 |
| 225 | rs4409785 | -0.0140763 | 0.0270333 | 0.602572 |
| 226 | rs45577137 | -0.00056168 | 0.0390974 | 0.988538 |
| 227 | rs460631 | 0.0320158 | 0.0416787 | 0.442393 |
| 228 | rs4618204 | 0.0355482 | 0.0201439 | 0.077612 |
| 229 | rs4652560 | -0.0140187 | 0.0221459 | 0.526725 |
| 230 | rs4675190 | 0.00891103 | 0.0204485 | 0.662998 |
| 231 | rs4680250 | -0.0135488 | 0.0222881 | 0.543258 |
| 232 | rs4703589 | -0.0189078 | 0.0205985 | 0.358660 |
| 233 | rs4703730 | -0.01228 | 0.0205918 | 0.550940 |
| 234 | rs4721559 | 0.00495598 | 0.0224175 | 0.825033 |
| 235 | rs473739 | -0.00830089 | 0.0231125 | 0.719482 |
| 236 | rs4746153 | 0.0043067 | 0.0263823 | 0.870328 |
| 237 | rs4849903 | 0.0207181 | 0.0207675 | 0.318463 |
| 238 | rs4870977 | 0.0142147 | 0.0307637 | 0.644037 |
| 239 | rs4908835 | 0.0496909 | 0.0270004 | 0.065714 |
| 240 | rs4931002 | -0.0814949 | 0.0252376 | 0.001241 |
| 241 | rs495149 | -0.0179123 | 0.0278938 | 0.520769 |
| 242 | rs496475 | 0.0261156 | 0.0212575 | 0.219246 |
| 243 | rs547211157 | 0.0215767 | 0.0839561 | 0.797179 |
| 244 | rs556063 | 0.0189487 | 0.0210697 | 0.368475 |
| 245 | rs55868524 | 0.0478031 | 0.0208732 | 0.022012 |
| 246 | rs55879743 | 0.0323044 | 0.0547733 | 0.555335 |
| 247 | rs55977204 | -0.0449892 | 0.0440132 | 0.306697 |
| 248 | rs56117721 | -0.0478352 | 0.0324933 | 0.140978 |
| 249 | rs56179563 | 0.00312044 | 0.0211428 | 0.882667 |
| 250 | rs56268488 | -0.00260057 | 0.045134 | 0.954052 |
| 251 | rs56330463 | 0.02161 | 0.0208717 | 0.300496 |
| 252 | rs574183 | -0.0306807 | 0.0206985 | 0.138270 |
| 253 | rs5747308 | 0.0309428 | 0.0201789 | 0.125171 |
| 254 | rs57633475 | 0.0327094 | 0.0271664 | 0.228575 |
| 255 | rs57834782 | -0.0317062 | 0.0213279 | 0.137118 |
| 256 | rs58745116 | 0.0116044 | 0.021925 | 0.596612 |
| 257 | rs58833930 | 0.0331924 | 0.0279327 | 0.234716 |
| 258 | rs594479 | -0.0199854 | 0.021342 | 0.349049 |
| 259 | rs60175411 | -0.0173178 | 0.0279125 | 0.534974 |
| 260 | rs60600003 | 0.0024766 | 0.0334854 | 0.941042 |
| 261 | rs6080761 | 0.00270169 | 0.0203895 | 0.894585 |
| 262 | rs6103572 | -0.0306866 | 0.0224386 | 0.171444 |
| 263 | rs6139104 | -0.0551192 | 0.0359881 | 0.125623 |
| 264 | rs6141755 | -0.0380042 | 0.0216409 | 0.079066 |
| 265 | rs61426394 | 0.0936158 | 0.0475221 | 0.048845 |
| 266 | rs61798836 | 0.0305126 | 0.0250009 | 0.222292 |
| 267 | rs62006172 | -0.0627723 | 0.0546118 | 0.250380 |
| 268 | rs62011287 | -0.0332911 | 0.0206663 | 0.107204 |
| 269 | rs62061733 | -0.0762739 | 0.0367521 | 0.037952 |
| 270 | rs62086903 | 0.0375915 | 0.0225533 | 0.095556 |
| 271 | rs62105489 | 0.00878183 | 0.0829935 | 0.915730 |
| 272 | rs62117160 | -0.158212 | 0.0624719 | 0.011324 |
| 273 | rs62183994 | 0.0899213 | 0.0514828 | 0.080703 |
| 274 | rs62308111 | -0.0119291 | 0.0252826 | 0.637049 |
| 275 | rs62385501 | 0.0302563 | 0.0242512 | 0.212169 |
| 276 | rs62395833 | 0.140063 | 0.0476073 | 0.003260 |
| 277 | rs62408224 | -0.087054 | 0.0235166 | 0.000214 |
| 278 | rs62420764 | 0.0955423 | 0.0247246 | 0.000111 |
| 279 | rs62473720 | 0.0365767 | 0.02146 | 0.088303 |
| 280 | rs634534 | 0.0553076 | 0.0206331 | 0.007350 |
| 281 | rs637064 | -0.0136853 | 0.0208738 | 0.512071 |
| 282 | rs6479336 | 0.0133588 | 0.0235193 | 0.570041 |
| 283 | rs6490291 | 0.0275781 | 0.0497556 | 0.579394 |
| 284 | rs6496717 | 0.0430928 | 0.022764 | 0.058355 |
| 285 | rs6540985 | -0.0540942 | 0.0204489 | 0.008160 |
| 286 | rs6556313 | 0.0314714 | 0.0209822 | 0.133637 |
| 287 | rs6573020 | 0.0356606 | 0.0202994 | 0.078964 |
| 288 | rs668248 | 0.00481537 | 0.0216044 | 0.823622 |
| 289 | rs6684992 | -0.0165665 | 0.0296814 | 0.576747 |
| 290 | rs6691839 | -0.0342144 | 0.0225555 | 0.129293 |
| 291 | rs6731125 | 0.0168571 | 0.0206394 | 0.414074 |
| 292 | rs6750754 | -0.0171563 | 0.0227347 | 0.450471 |
| 293 | rs67856193 | 0.0421376 | 0.0213664 | 0.048592 |
| 294 | rs6787336 | 0.00396122 | 0.0230942 | 0.863812 |
| 295 | rs6904506 | -0.0435781 | 0.0397307 | 0.272712 |
| 296 | rs6924350 | 0.0568993 | 0.0245267 | 0.020347 |
| 297 | rs6924387 | 0.0500467 | 0.0204062 | 0.014186 |
| 298 | rs6956283 | -0.043241 | 0.0240412 | 0.072079 |
| 299 | rs6971710 | -0.0029497 | 0.024555 | 0.904383 |
| 300 | rs6979947 | -0.0105182 | 0.0229172 | 0.646257 |
| 301 | rs6986109 | -0.0594144 | 0.0231424 | 0.010248 |
| 302 | rs6989099 | -0.0269662 | 0.0225088 | 0.230905 |
| 303 | rs699664 | -0.00046694 | 0.0216074 | 0.982759 |
| 304 | rs6999452 | 0.0054616 | 0.0209573 | 0.794396 |
| 305 | rs7026022 | 0.0259745 | 0.0214074 | 0.224999 |
| 306 | rs7080536 | -0.0919938 | 0.0621242 | 0.138658 |
| 307 | rs708776 | 0.159523 | 0.0517905 | 0.002068 |
| 308 | rs7141943 | -0.00862044 | 0.0207744 | 0.678175 |
| 309 | rs71429414 | 0.0195652 | 0.0276999 | 0.479984 |
| 310 | rs71508968 | -0.160461 | 0.074347 | 0.030906 |
| 311 | rs7158239 | 0.0572794 | 0.0204408 | 0.005075 |
| 312 | rs71628184 | 0.0184098 | 0.0388988 | 0.636017 |
| 313 | rs7215391 | -0.0274431 | 0.0250821 | 0.273897 |
| 314 | rs7220649 | -0.0405294 | 0.0709916 | 0.568064 |
| 315 | rs725613 | -0.0465601 | 0.0213999 | 0.029576 |
| 316 | rs7257 | -0.027623 | 0.0202928 | 0.173445 |
| 317 | rs72766638 | -0.0607814 | 0.0249952 | 0.015027 |
| 318 | rs72834751 | -0.104056 | 0.0750155 | 0.165402 |
| 319 | rs72844043 | 0.0165057 | 0.0202703 | 0.415487 |
| 320 | rs72987040 | 0.0711782 | 0.0281314 | 0.011399 |
| 321 | rs72998585 | -0.135241 | 0.0272109 | 6.6921E-07 |
| 322 | rs73049239 | 0.00920185 | 0.0619874 | 0.881990 |
| 323 | rs73072498 | 0.133371 | 0.118883 | 0.261921 |
| 324 | rs73118830 | -0.00310353 | 0.0245705 | 0.899486 |
| 325 | rs73176183 | 0.02599 | 0.033292 | 0.434997 |
| 326 | rs73176685 | 0.0184125 | 0.0233164 | 0.429714 |
| 327 | rs73187852 | 0.00737992 | 0.0230699 | 0.749049 |
| 328 | rs73202462 | 0.0828983 | 0.242519 | 0.732485 |
| 329 | rs73203442 | -0.0675581 | 0.0345619 | 0.050618 |
| 330 | rs73232881 | 0.00477138 | 0.0266867 | 0.858101 |
| 331 | rs73238201 | -0.02253 | 0.0264516 | 0.394356 |
| 332 | rs73272842 | -0.0145504 | 0.0302096 | 0.630054 |
| 333 | rs7327960 | -0.0272173 | 0.0290048 | 0.348052 |
| 334 | rs73322872 | 0.0524637 | 0.0254169 | 0.039005 |
| 335 | rs73428834 | 0.0304092 | 0.0323892 | 0.347799 |
| 336 | rs7382061 | 0.00730326 | 0.0201875 | 0.717523 |
| 337 | rs73963711 | 0.0076659 | 0.021376 | 0.719878 |
| 338 | rs7423615 | 0.00111105 | 0.0300517 | 0.970508 |
| 339 | rs74299961 | 0.00470303 | 0.0243466 | 0.846826 |
| 340 | rs743002 | -0.0424432 | 0.0522748 | 0.416835 |
| 341 | rs74331768 | -0.0153216 | 0.0403406 | 0.704090 |
| 342 | rs7441808 | -0.0169994 | 0.0223929 | 0.447768 |
| 343 | rs74480102 | -0.0299777 | 0.0709647 | 0.672710 |
| 344 | rs74612091 | -0.0400602 | 0.0394124 | 0.309422 |
| 345 | rs746550 | 0.0158776 | 0.0294022 | 0.589187 |
| 346 | rs7569084 | -0.00233453 | 0.0210637 | 0.911750 |
| 347 | rs7636495 | -0.030668 | 0.0257969 | 0.234509 |
| 348 | rs7646283 | 0.0456745 | 0.0215933 | 0.034412 |
| 349 | rs7646695 | -0.012248 | 0.0264079 | 0.642790 |
| 350 | rs76474320 | -0.0515629 | 0.0426635 | 0.226818 |
| 351 | rs7649812 | 0.0215549 | 0.0225195 | 0.338484 |
| 352 | rs76639817 | 0.116115 | 0.037354 | 0.001880 |
| 353 | rs76793172 | 0.00268325 | 0.0283056 | 0.924477 |
| 354 | rs76830965 | 0.0362636 | 0.0368433 | 0.324985 |
| 355 | rs7687708 | 0.0321103 | 0.0251003 | 0.200799 |
| 356 | rs76908370 | -0.0561796 | 0.0462299 | 0.224281 |
| 357 | rs76981581 | -0.0600047 | 0.0331718 | 0.070466 |
| 358 | rs7700687 | 0.0442667 | 0.0212189 | 0.036960 |
| 359 | rs77625297 | -0.160165 | 0.0750435 | 0.032818 |
| 360 | rs778756 | 0.00822171 | 0.0202476 | 0.684699 |
| 361 | rs7797428 | 0.0500535 | 0.0203737 | 0.014019 |
| 362 | rs783646 | -0.0720062 | 0.0221678 | 0.001161 |
| 363 | rs7839946 | 0.0323632 | 0.042782 | 0.449368 |
| 364 | rs7840212 | -0.0123427 | 0.022257 | 0.579201 |
| 365 | rs7846314 | -0.00043312 | 0.0255693 | 0.986485 |
| 366 | rs7868130 | 0.0797672 | 0.0213159 | 0.000182 |
| 367 | rs78691875 | 0.374795 | 0.145593 | 0.010045 |
| 368 | rs7897422 | 0.0354233 | 0.0218937 | 0.105670 |
| 369 | rs79716587 | -0.00710816 | 0.0366585 | 0.846252 |
| 370 | rs7975 | -0.014282 | 0.0206112 | 0.488359 |
| 371 | rs7986796 | 0.00151494 | 0.0208822 | 0.942167 |
| 372 | rs8 | 0.0247044 | 0.0240576 | 0.304474 |
| 373 | rs80054178 | 0.208888 | 0.110511 | 0.058731 |
| 374 | rs80066203 | -0.0361345 | 0.0515432 | 0.483270 |
| 375 | rs8012643 | 0.0234097 | 0.0233945 | 0.316996 |
| 376 | rs8020739 | 0.0496853 | 0.0216289 | 0.021608 |
| 377 | rs8026803 | -0.0457918 | 0.0240949 | 0.057370 |
| 378 | rs8044920 | -0.0534837 | 0.0209374 | 0.010635 |
| 379 | rs8050508 | -0.0386903 | 0.034905 | 0.267670 |
| 380 | rs8061729 | -0.0393551 | 0.0233002 | 0.091209 |
| 381 | rs8076052 | 0.0216193 | 0.0242278 | 0.372216 |
| 382 | rs8108623 | 0.00758522 | 0.0213743 | 0.722683 |
| 383 | rs8142080 | 0.0132326 | 0.0341321 | 0.698247 |
| 384 | rs884634 | 0.0473821 | 0.0204813 | 0.020699 |
| 385 | rs911603 | 0.0213416 | 0.0202665 | 0.292320 |
| 386 | rs925966 | 0.0133214 | 0.0215311 | 0.536113 |
| 387 | rs9349322 | -0.0456147 | 0.0249641 | 0.067667 |
| 388 | rs9389268 | 0.0357936 | 0.0212682 | 0.092382 |
| 389 | rs9392525 | 0.0384248 | 0.020312 | 0.058527 |
| 390 | rs941616 | -0.0533261 | 0.0205241 | 0.009370 |
| 391 | rs954954 | -0.01681 | 0.0309965 | 0.587598 |
| 392 | rs962993 | -0.0925875 | 0.022119 | 2.8405E-05 |
| 393 | rs964184 | -0.0131224 | 0.0288354 | 0.649051 |
| 394 | rs9666598 | -0.0260085 | 0.026574 | 0.327720 |
| 395 | rs9675999 | 0.022302 | 0.0211373 | 0.291380 |
| 396 | rs9815874 | 0.0719707 | 0.0243542 | 0.003124 |
| 397 | rs9818987 | -0.0178498 | 0.0204513 | 0.382775 |
| 398 | rs9835307 | 0.0424549 | 0.0215395 | 0.048720 |
| 399 | rs9837045 | -0.020677 | 0.0201411 | 0.304606 |
| 400 | rs9840310 | -0.0302776 | 0.0229277 | 0.186645 |
| 401 | rs9872485 | -0.00838358 | 0.0241039 | 0.727983 |
| 402 | rs9880192 | -0.0373533 | 0.0213976 | 0.080866 |
| 403 | rs9894839 | -0.0298681 | 0.0205469 | 0.146041 |
| 404 | rs9939774 | 0.0370965 | 0.0208555 | 0.075281 |
| 405 | rs9979383 | 0.0470473 | 0.0212123 | 0.026559 |

SNPs: single-nucleotide polymorphisms; se: standard error; EOS: eosinophil

Supplementary Table 14：Leave-one-out analysis for estimate of EOS-Asthma.

|  | SNPs | beta | se | p |
| --- | --- | --- | --- | --- |
| 1 | rs1004870 | 0.6070339 | 0.0617633 | <0.001 |
| 2 | rs10059018 | 0.6083686 | 0.0617110 | <0.001 |
| 3 | rs10062687 | 0.6085943 | 0.0617537 | <0.001 |
| 4 | rs10100356 | 0.6114256 | 0.0617417 | <0.001 |
| 5 | rs10165678 | 0.6082940 | 0.0616444 | <0.001 |
| 6 | rs10174238 | 0.6107317 | 0.0617548 | <0.001 |
| 7 | rs10195713 | 0.6086033 | 0.0617026 | <0.001 |
| 8 | rs1036332 | 0.6165121 | 0.0617278 | <0.001 |
| 9 | rs1037674 | 0.6128900 | 0.0616288 | <0.001 |
| 10 | rs1039341 | 0.6124765 | 0.0618026 | <0.001 |
| 11 | rs10472984 | 0.6033234 | 0.0617513 | <0.001 |
| 12 | rs1047891 | 0.6119830 | 0.0617519 | <0.001 |
| 13 | rs1057258 | 0.6048863 | 0.0615442 | <0.001 |
| 14 | rs1059091 | 0.6097485 | 0.0618954 | <0.001 |
| 15 | rs10745763 | 0.6109255 | 0.0617624 | <0.001 |
| 16 | rs10777378 | 0.6102752 | 0.0618154 | <0.001 |
| 17 | rs10782957 | 0.6115433 | 0.0618062 | <0.001 |
| 18 | rs10876550 | 0.6128410 | 0.0616590 | <0.001 |
| 19 | rs10900595 | 0.6118078 | 0.0617296 | <0.001 |
| 20 | rs10930337 | 0.6147756 | 0.0614350 | <0.001 |
| 21 | rs1099448 | 0.6082572 | 0.0617104 | <0.001 |
| 22 | rs10995240 | 0.6040296 | 0.0619104 | <0.001 |
| 23 | rs11065822 | 0.6227042 | 0.0621463 | <0.001 |
| 24 | rs11071528 | 0.6109675 | 0.0617495 | <0.001 |
| 25 | rs11079340 | 0.6115330 | 0.0617410 | <0.001 |
| 26 | rs11088236 | 0.6111523 | 0.0617872 | <0.001 |
| 27 | rs111759324 | 0.6117854 | 0.0618055 | <0.001 |
| 28 | rs112036266 | 0.6095604 | 0.0616765 | <0.001 |
| 29 | rs11204682 | 0.6154715 | 0.0618374 | <0.001 |
| 30 | rs11228990 | 0.6100433 | 0.0617466 | <0.001 |
| 31 | rs11236813 | 0.6023711 | 0.0610743 | <0.001 |
| 32 | rs11255507 | 0.6082136 | 0.0616966 | <0.001 |
| 33 | rs113473633 | 0.6144616 | 0.0615213 | <0.001 |
| 34 | rs113496608 | 0.6092193 | 0.0616918 | <0.001 |
| 35 | rs113542380 | 0.6114917 | 0.0617256 | <0.001 |
| 36 | rs113859409 | 0.6120952 | 0.0617199 | <0.001 |
| 37 | rs114152720 | 0.6101776 | 0.0617607 | <0.001 |
| 38 | rs114741563 | 0.6106915 | 0.0617676 | <0.001 |
| 39 | rs11555542 | 0.6123596 | 0.0617408 | <0.001 |
| 40 | rs115647629 | 0.6126828 | 0.0617514 | <0.001 |
| 41 | rs11571404 | 0.6091445 | 0.0616964 | <0.001 |
| 42 | rs11578794 | 0.6094824 | 0.0617199 | <0.001 |
| 43 | rs11628569 | 0.6132253 | 0.0616827 | <0.001 |
| 44 | rs11647946 | 0.6117735 | 0.0617315 | <0.001 |
| 45 | rs11684770 | 0.6100407 | 0.0617526 | <0.001 |
| 46 | rs116888884 | 0.6102299 | 0.0617244 | <0.001 |
| 47 | rs11695281 | 0.6063352 | 0.0616841 | <0.001 |
| 48 | rs11701475 | 0.6125061 | 0.0616899 | <0.001 |
| 49 | rs11702918 | 0.6117642 | 0.0617376 | <0.001 |
| 50 | rs1170439 | 0.6099364 | 0.0617744 | <0.001 |
| 51 | rs117068593 | 0.6078467 | 0.0616948 | <0.001 |
| 52 | rs117481629 | 0.6104908 | 0.0617495 | <0.001 |
| 53 | rs1178016 | 0.6119627 | 0.0617099 | <0.001 |
| 54 | rs11786536 | 0.6123323 | 0.0617807 | <0.001 |
| 55 | rs117955557 | 0.6135063 | 0.0618234 | <0.001 |
| 56 | rs117961539 | 0.6108586 | 0.0617517 | <0.001 |
| 57 | rs118013485 | 0.6036345 | 0.0612963 | <0.001 |
| 58 | rs11869228 | 0.6090387 | 0.0617234 | <0.001 |
| 59 | rs11886928 | 0.6125645 | 0.0616689 | <0.001 |
| 60 | rs11931711 | 0.6113468 | 0.0617844 | <0.001 |
| 61 | rs12100034 | 0.6126783 | 0.0617207 | <0.001 |
| 62 | rs12154498 | 0.6123868 | 0.0618227 | <0.001 |
| 63 | rs121564 | 0.6070818 | 0.0615132 | <0.001 |
| 64 | rs12208103 | 0.6088369 | 0.0618678 | <0.001 |
| 65 | rs12361586 | 0.6105161 | 0.0617505 | <0.001 |
| 66 | rs12408934 | 0.6135474 | 0.0618059 | <0.001 |
| 67 | rs12470046 | 0.6115504 | 0.0617360 | <0.001 |
| 68 | rs12487980 | 0.6113150 | 0.0617439 | <0.001 |
| 69 | rs12530946 | 0.6198844 | 0.0618032 | <0.001 |
| 70 | rs12540285 | 0.6106488 | 0.0617644 | <0.001 |
| 71 | rs12545733 | 0.6121833 | 0.0618568 | <0.001 |
| 72 | rs1257192 | 0.6106449 | 0.0617526 | <0.001 |
| 73 | rs12581511 | 0.6101395 | 0.0617601 | <0.001 |
| 74 | rs12705849 | 0.6159575 | 0.0615332 | <0.001 |
| 75 | rs12820863 | 0.6116545 | 0.0618027 | <0.001 |
| 76 | rs12861824 | 0.6095574 | 0.0617761 | <0.001 |
| 77 | rs12878610 | 0.6122545 | 0.0617115 | <0.001 |
| 78 | rs12882281 | 0.6110653 | 0.0617500 | <0.001 |
| 79 | rs12928503 | 0.6125877 | 0.0614481 | <0.001 |
| 80 | rs12941068 | 0.6081156 | 0.0615571 | <0.001 |
| 81 | rs12978850 | 0.6091007 | 0.0616581 | <0.001 |
| 82 | rs13073683 | 0.6124105 | 0.0617297 | <0.001 |
| 83 | rs13105682 | 0.6120133 | 0.0614931 | <0.001 |
| 84 | rs13138355 | 0.6128528 | 0.0620881 | <0.001 |
| 85 | rs13139941 | 0.6105081 | 0.0617535 | <0.001 |
| 86 | rs13207791 | 0.6109565 | 0.0617409 | <0.001 |
| 87 | rs13226583 | 0.6160516 | 0.0619609 | <0.001 |
| 88 | rs1323650 | 0.6118217 | 0.0617300 | <0.001 |
| 89 | rs13251643 | 0.6102014 | 0.0617577 | <0.001 |
| 90 | rs13313564 | 0.6100940 | 0.0618594 | <0.001 |
| 91 | rs13511 | 0.6077502 | 0.0616520 | <0.001 |
| 92 | rs1353286 | 0.6110800 | 0.0617997 | <0.001 |
| 93 | rs1365623 | 0.6109051 | 0.0617536 | <0.001 |
| 94 | rs137906075 | 0.6108475 | 0.0617770 | <0.001 |
| 95 | rs1395269 | 0.6147062 | 0.0616964 | <0.001 |
| 96 | rs139640694 | 0.6083448 | 0.0616676 | <0.001 |
| 97 | rs1406449 | 0.6122878 | 0.0616965 | <0.001 |
| 98 | rs1414517 | 0.6097510 | 0.0617801 | <0.001 |
| 99 | rs1427499 | 0.6109997 | 0.0617818 | <0.001 |
| 100 | rs143491704 | 0.6115110 | 0.0617051 | <0.001 |
| 101 | rs144569746 | 0.6118796 | 0.0619450 | <0.001 |
| 102 | rs1448187 | 0.6102227 | 0.0617617 | <0.001 |
| 103 | rs145947882 | 0.6159187 | 0.0615481 | <0.001 |
| 104 | rs146078144 | 0.6115244 | 0.0617145 | <0.001 |
| 105 | rs146730870 | 0.6215360 | 0.0614564 | <0.001 |
| 106 | rs14713 | 0.6092610 | 0.0616936 | <0.001 |
| 107 | rs1471816 | 0.6115966 | 0.0617295 | <0.001 |
| 108 | rs1479918 | 0.6085209 | 0.0616536 | <0.001 |
| 109 | rs148219449 | 0.6099347 | 0.0617172 | <0.001 |
| 110 | rs149110519 | 0.6118900 | 0.0617222 | <0.001 |
| 111 | rs150640087 | 0.6167851 | 0.0616183 | <0.001 |
| 112 | rs1516527 | 0.6103596 | 0.0617483 | <0.001 |
| 113 | rs1519602 | 0.6088756 | 0.0616686 | <0.001 |
| 114 | rs1529745 | 0.6112704 | 0.0617397 | <0.001 |
| 115 | rs1539174 | 0.6107529 | 0.0618896 | <0.001 |
| 116 | rs1547258 | 0.6109264 | 0.0617723 | <0.001 |
| 117 | rs159963 | 0.6108938 | 0.0617885 | <0.001 |
| 118 | rs1672753 | 0.6091759 | 0.0617245 | <0.001 |
| 119 | rs1684578 | 0.6092623 | 0.0617420 | <0.001 |
| 120 | rs1689510 | 0.6080571 | 0.0617910 | <0.001 |
| 121 | rs16903574 | 0.6070743 | 0.0616554 | <0.001 |
| 122 | rs16956811 | 0.6104248 | 0.0617585 | <0.001 |
| 123 | rs17061503 | 0.6081684 | 0.0619059 | <0.001 |
| 124 | rs17175830 | 0.6102245 | 0.0618543 | <0.001 |
| 125 | rs174548 | 0.6119674 | 0.0618063 | <0.001 |
| 126 | rs17482472 | 0.6115161 | 0.0617695 | <0.001 |
| 127 | rs17516457 | 0.5985149 | 0.0621040 | <0.001 |
| 128 | rs175705 | 0.6067499 | 0.0619226 | <0.001 |
| 129 | rs17653687 | 0.6099424 | 0.0617448 | <0.001 |
| 130 | rs17668272 | 0.6065650 | 0.0616671 | <0.001 |
| 131 | rs17682575 | 0.6146284 | 0.0614227 | <0.001 |
| 132 | rs17689159 | 0.6098059 | 0.0617452 | <0.001 |
| 133 | rs17758695 | 0.6213126 | 0.0619765 | <0.001 |
| 134 | rs17849501 | 0.6112855 | 0.0617406 | <0.001 |
| 135 | rs1800692 | 0.6114899 | 0.0617769 | <0.001 |
| 136 | rs180506 | 0.6128125 | 0.0617325 | <0.001 |
| 137 | rs1828803 | 0.6071670 | 0.0614541 | <0.001 |
| 138 | rs1861489 | 0.6111268 | 0.0617504 | <0.001 |
| 139 | rs201798 | 0.6096380 | 0.0617795 | <0.001 |
| 140 | rs2025489 | 0.6088275 | 0.0617206 | <0.001 |
| 141 | rs2089979 | 0.6092017 | 0.0617305 | <0.001 |
| 142 | rs2182885 | 0.6047268 | 0.0616522 | <0.001 |
| 143 | rs2223043 | 0.6100639 | 0.0617863 | <0.001 |
| 144 | rs2228467 | 0.6196146 | 0.0616471 | <0.001 |
| 145 | rs2239633 | 0.6117310 | 0.0619301 | <0.001 |
| 146 | rs2253427 | 0.6119397 | 0.0617064 | <0.001 |
| 147 | rs2399441 | 0.6085640 | 0.0617266 | <0.001 |
| 148 | rs2410732 | 0.6105335 | 0.0617588 | <0.001 |
| 149 | rs2419313 | 0.6129034 | 0.0616799 | <0.001 |
| 150 | rs2431097 | 0.6102708 | 0.0617676 | <0.001 |
| 151 | rs2497318 | 0.6048839 | 0.0617355 | <0.001 |
| 152 | rs2502995 | 0.6040144 | 0.0614476 | <0.001 |
| 153 | rs2505521 | 0.6119693 | 0.0617240 | <0.001 |
| 154 | rs2566133 | 0.6080587 | 0.0616371 | <0.001 |
| 155 | rs2579505 | 0.6180065 | 0.0618794 | <0.001 |
| 156 | rs2646438 | 0.6098794 | 0.0617954 | <0.001 |
| 157 | rs2713548 | 0.6099103 | 0.0617416 | <0.001 |
| 158 | rs2788211 | 0.6117050 | 0.0617335 | <0.001 |
| 159 | rs2807740 | 0.6127386 | 0.0618443 | <0.001 |
| 160 | rs2817377 | 0.6108071 | 0.0617549 | <0.001 |
| 161 | rs28362902 | 0.6106432 | 0.0617543 | <0.001 |
| 162 | rs2838317 | 0.6142206 | 0.0614433 | <0.001 |
| 163 | rs28421324 | 0.6171295 | 0.0615483 | <0.001 |
| 164 | rs2847266 | 0.6119401 | 0.0617308 | <0.001 |
| 165 | rs2850542 | 0.6059366 | 0.0612721 | <0.001 |
| 166 | rs28532037 | 0.6119298 | 0.0617771 | <0.001 |
| 167 | rs2864936 | 0.6114327 | 0.0617300 | <0.001 |
| 168 | rs2887502 | 0.6104811 | 0.0617623 | <0.001 |
| 169 | rs2894401 | 0.6116800 | 0.0617697 | <0.001 |
| 170 | rs290430 | 0.6131098 | 0.0615999 | <0.001 |
| 171 | rs2920505 | 0.6060393 | 0.0617832 | <0.001 |
| 172 | rs295 | 0.6137059 | 0.0615749 | <0.001 |
| 173 | rs295273 | 0.6138265 | 0.0616745 | <0.001 |
| 174 | rs2979489 | 0.6104980 | 0.0617585 | <0.001 |
| 175 | rs2992333 | 0.6148671 | 0.0618367 | <0.001 |
| 176 | rs301162 | 0.6098623 | 0.0617715 | <0.001 |
| 177 | rs3024971 | 0.6070766 | 0.0615785 | <0.001 |
| 178 | rs3093023 | 0.6106297 | 0.0617598 | <0.001 |
| 179 | rs3096309 | 0.6095223 | 0.0616936 | <0.001 |
| 180 | rs3110791 | 0.6120347 | 0.0617355 | <0.001 |
| 181 | rs3218148 | 0.6132848 | 0.0616927 | <0.001 |
| 182 | rs33982662 | 0.6117092 | 0.0617746 | <0.001 |
| 183 | rs34173062 | 0.6080917 | 0.0617898 | <0.001 |
| 184 | rs34210653 | 0.6117372 | 0.0618705 | <0.001 |
| 185 | rs34212866 | 0.6116973 | 0.0617490 | <0.001 |
| 186 | rs34363176 | 0.6092596 | 0.0616761 | <0.001 |
| 187 | rs34439695 | 0.6106997 | 0.0617583 | <0.001 |
| 188 | rs34448954 | 0.6088898 | 0.0617225 | <0.001 |
| 189 | rs34466956 | 0.6097914 | 0.0617456 | <0.001 |
| 190 | rs34495 | 0.6128639 | 0.0616909 | <0.001 |
| 191 | rs34505104 | 0.6131681 | 0.0617831 | <0.001 |
| 192 | rs34631302 | 0.6123575 | 0.0616826 | <0.001 |
| 193 | rs350836 | 0.6114075 | 0.0617638 | <0.001 |
| 194 | rs35249183 | 0.6100414 | 0.0617767 | <0.001 |
| 195 | rs35409523 | 0.6167692 | 0.0617219 | <0.001 |
| 196 | rs36084354 | 0.6113364 | 0.0618374 | <0.001 |
| 197 | rs3731211 | 0.6090247 | 0.0617204 | <0.001 |
| 198 | rs3742704 | 0.6104394 | 0.0617500 | <0.001 |
| 199 | rs3746420 | 0.6098738 | 0.0617524 | <0.001 |
| 200 | rs3747869 | 0.6115904 | 0.0617618 | <0.001 |
| 201 | rs3757114 | 0.6131868 | 0.0617990 | <0.001 |
| 202 | rs3785356 | 0.6042838 | 0.0616391 | <0.001 |
| 203 | rs3786586 | 0.6124216 | 0.0618238 | <0.001 |
| 204 | rs3790163 | 0.6112455 | 0.0617480 | <0.001 |
| 205 | rs3804590 | 0.6101294 | 0.0617745 | <0.001 |
| 206 | rs3812206 | 0.6090450 | 0.0616768 | <0.001 |
| 207 | rs3823536 | 0.6107166 | 0.0617855 | <0.001 |
| 208 | rs3824867 | 0.6127166 | 0.0617234 | <0.001 |
| 209 | rs3846855 | 0.6166981 | 0.0616525 | <0.001 |
| 210 | rs3850107 | 0.6103732 | 0.0617558 | <0.001 |
| 211 | rs3950296 | 0.6104286 | 0.0618061 | <0.001 |
| 212 | rs397187 | 0.6097231 | 0.0617297 | <0.001 |
| 213 | rs4074672 | 0.6106139 | 0.0617625 | <0.001 |
| 214 | rs410867 | 0.6069581 | 0.0621230 | <0.001 |
| 215 | rs412884 | 0.6128941 | 0.0622039 | <0.001 |
| 216 | rs41313381 | 0.6099163 | 0.0617460 | <0.001 |
| 217 | rs4142528 | 0.5951752 | 0.0623226 | <0.001 |
| 218 | rs4148757 | 0.6092331 | 0.0616943 | <0.001 |
| 219 | rs4149909 | 0.6092695 | 0.0617008 | <0.001 |
| 220 | rs4236746 | 0.6106974 | 0.0617813 | <0.001 |
| 221 | rs4240624 | 0.6095884 | 0.0617444 | <0.001 |
| 222 | rs4280242 | 0.6143089 | 0.0618095 | <0.001 |
| 223 | rs4310436 | 0.6103059 | 0.0617580 | <0.001 |
| 224 | rs4347868 | 0.6116638 | 0.0617169 | <0.001 |
| 225 | rs4409785 | 0.6119047 | 0.0617239 | <0.001 |
| 226 | rs45577137 | 0.6137986 | 0.0618544 | <0.001 |
| 227 | rs460631 | 0.6101958 | 0.0617527 | <0.001 |
| 228 | rs4618204 | 0.6083315 | 0.0617241 | <0.001 |
| 229 | rs4652560 | 0.6104062 | 0.0617646 | <0.001 |
| 230 | rs4675190 | 0.6116977 | 0.0617289 | <0.001 |
| 231 | rs4680250 | 0.6104501 | 0.0617661 | <0.001 |
| 232 | rs4703589 | 0.6127825 | 0.0616875 | <0.001 |
| 233 | rs4703730 | 0.6105167 | 0.0617741 | <0.001 |
| 234 | rs4721559 | 0.6119021 | 0.0617534 | <0.001 |
| 235 | rs473739 | 0.6107323 | 0.0617582 | <0.001 |
| 236 | rs4746153 | 0.6109803 | 0.0617559 | <0.001 |
| 237 | rs4849903 | 0.6098866 | 0.0617975 | <0.001 |
| 238 | rs4870977 | 0.6118310 | 0.0617284 | <0.001 |
| 239 | rs4908835 | 0.6085426 | 0.0616811 | <0.001 |
| 240 | rs4931002 | 0.6056712 | 0.0614607 | <0.001 |
| 241 | rs495149 | 0.6129513 | 0.0617244 | <0.001 |
| 242 | rs496475 | 0.6099189 | 0.0619151 | <0.001 |
| 243 | rs547211157 | 0.6108598 | 0.0617572 | <0.001 |
| 244 | rs556063 | 0.6100224 | 0.0617465 | <0.001 |
| 245 | rs55868524 | 0.6081049 | 0.0616012 | <0.001 |
| 246 | rs55879743 | 0.6115791 | 0.0618662 | <0.001 |
| 247 | rs55977204 | 0.6119877 | 0.0616738 | <0.001 |
| 248 | rs56117721 | 0.6142330 | 0.0624293 | <0.001 |
| 249 | rs56179563 | 0.6114010 | 0.0617760 | <0.001 |
| 250 | rs56268488 | 0.6109497 | 0.0617461 | <0.001 |
| 251 | rs56330463 | 0.6112206 | 0.0619754 | <0.001 |
| 252 | rs574183 | 0.6091581 | 0.0617132 | <0.001 |
| 253 | rs5747308 | 0.6089339 | 0.0617245 | <0.001 |
| 254 | rs57633475 | 0.6142044 | 0.0616535 | <0.001 |
| 255 | rs57834782 | 0.6118261 | 0.0622409 | <0.001 |
| 256 | rs58745116 | 0.6121629 | 0.0617265 | <0.001 |
| 257 | rs58833930 | 0.6138234 | 0.0616542 | <0.001 |
| 258 | rs594479 | 0.6123530 | 0.0616835 | <0.001 |
| 259 | rs60175411 | 0.6107949 | 0.0618106 | <0.001 |
| 260 | rs60600003 | 0.6127075 | 0.0618225 | <0.001 |
| 261 | rs6080761 | 0.6115552 | 0.0617815 | <0.001 |
| 262 | rs6103572 | 0.6090530 | 0.0617891 | <0.001 |
| 263 | rs6139104 | 0.6093096 | 0.0616901 | <0.001 |
| 264 | rs6141755 | 0.6085496 | 0.0617025 | <0.001 |
| 265 | rs61426394 | 0.6133000 | 0.0615225 | <0.001 |
| 266 | rs61798836 | 0.6096254 | 0.0617238 | <0.001 |
| 267 | rs62006172 | 0.6096284 | 0.0617424 | <0.001 |
| 268 | rs62011287 | 0.6090674 | 0.0616923 | <0.001 |
| 269 | rs62061733 | 0.6077713 | 0.0616819 | <0.001 |
| 270 | rs62086903 | 0.6082349 | 0.0618254 | <0.001 |
| 271 | rs62105489 | 0.6109831 | 0.0617381 | <0.001 |
| 272 | rs62117160 | 0.6072876 | 0.0615852 | <0.001 |
| 273 | rs62183994 | 0.6090960 | 0.0616629 | <0.001 |
| 274 | rs62308111 | 0.6118416 | 0.0617277 | <0.001 |
| 275 | rs62385501 | 0.6128119 | 0.0616439 | <0.001 |
| 276 | rs62395833 | 0.6065599 | 0.0615058 | <0.001 |
| 277 | rs62408224 | 0.6004648 | 0.0616501 | <0.001 |
| 278 | rs62420764 | 0.6050377 | 0.0612656 | <0.001 |
| 279 | rs62473720 | 0.6088415 | 0.0616896 | <0.001 |
| 280 | rs634534 | 0.6047585 | 0.0617771 | <0.001 |
| 281 | rs637064 | 0.6141881 | 0.0617429 | <0.001 |
| 282 | rs6479336 | 0.6143209 | 0.0617604 | <0.001 |
| 283 | rs6490291 | 0.6146888 | 0.0617707 | <0.001 |
| 284 | rs6496717 | 0.6082141 | 0.0616957 | <0.001 |
| 285 | rs6540985 | 0.6064175 | 0.0616087 | <0.001 |
| 286 | rs6556313 | 0.6088321 | 0.0617536 | <0.001 |
| 287 | rs6573020 | 0.6082042 | 0.0617462 | <0.001 |
| 288 | rs668248 | 0.6118709 | 0.0617524 | <0.001 |
| 289 | rs6684992 | 0.6144202 | 0.0617642 | <0.001 |
| 290 | rs6691839 | 0.6092389 | 0.0616983 | <0.001 |
| 291 | rs6731125 | 0.6101618 | 0.0617763 | <0.001 |
| 292 | rs6750754 | 0.6167365 | 0.0622317 | <0.001 |
| 293 | rs67856193 | 0.6075877 | 0.0617426 | <0.001 |
| 294 | rs6787336 | 0.6134428 | 0.0618736 | <0.001 |
| 295 | rs6904506 | 0.6098812 | 0.0618474 | <0.001 |
| 296 | rs6924350 | 0.6059047 | 0.0619643 | <0.001 |
| 297 | rs6924387 | 0.6072417 | 0.0616110 | <0.001 |
| 298 | rs6956283 | 0.6087305 | 0.0616765 | <0.001 |
| 299 | rs6971710 | 0.6128008 | 0.0617937 | <0.001 |
| 300 | rs6979947 | 0.6117431 | 0.0617275 | <0.001 |
| 301 | rs6986109 | 0.6074090 | 0.0615683 | <0.001 |
| 302 | rs6989099 | 0.6094893 | 0.0617534 | <0.001 |
| 303 | rs699664 | 0.6113874 | 0.0617615 | <0.001 |
| 304 | rs6999452 | 0.6108171 | 0.0617544 | <0.001 |
| 305 | rs7026022 | 0.6096524 | 0.0617224 | <0.001 |
| 306 | rs7080536 | 0.6091095 | 0.0617187 | <0.001 |
| 307 | rs708776 | 0.6076068 | 0.0614161 | <0.001 |
| 308 | rs7141943 | 0.6121926 | 0.0617391 | <0.001 |
| 309 | rs71429414 | 0.6123542 | 0.0617096 | <0.001 |
| 310 | rs71508968 | 0.6133315 | 0.0614850 | <0.001 |
| 311 | rs7158239 | 0.6068408 | 0.0615328 | <0.001 |
| 312 | rs71628184 | 0.6105553 | 0.0617530 | <0.001 |
| 313 | rs7215391 | 0.6098570 | 0.0617241 | <0.001 |
| 314 | rs7220649 | 0.6104379 | 0.0617398 | <0.001 |
| 315 | rs725613 | 0.6067101 | 0.0620371 | <0.001 |
| 316 | rs7257 | 0.6193335 | 0.0616700 | <0.001 |
| 317 | rs72766638 | 0.6068198 | 0.0616491 | <0.001 |
| 318 | rs72834751 | 0.6090456 | 0.0617672 | <0.001 |
| 319 | rs72844043 | 0.6121395 | 0.0616964 | <0.001 |
| 320 | rs72987040 | 0.6157547 | 0.0613777 | <0.001 |
| 321 | rs72998585 | 0.5888733 | 0.0621050 | <0.001 |
| 322 | rs73049239 | 0.6111624 | 0.0617403 | <0.001 |
| 323 | rs73072498 | 0.6098268 | 0.0617221 | <0.001 |
| 324 | rs73118830 | 0.6122992 | 0.0618142 | <0.001 |
| 325 | rs73176183 | 0.6121819 | 0.0617002 | <0.001 |
| 326 | rs73176685 | 0.6103527 | 0.0618031 | <0.001 |
| 327 | rs73187852 | 0.6110129 | 0.0617755 | <0.001 |
| 328 | rs73202462 | 0.6106208 | 0.0617359 | <0.001 |
| 329 | rs73203442 | 0.6091006 | 0.0622525 | <0.001 |
| 330 | rs73232881 | 0.6183290 | 0.0620841 | <0.001 |
| 331 | rs73238201 | 0.6100820 | 0.0617503 | <0.001 |
| 332 | rs73272842 | 0.6107789 | 0.0617813 | <0.001 |
| 333 | rs7327960 | 0.6124617 | 0.0616836 | <0.001 |
| 334 | rs73322872 | 0.6085800 | 0.0616271 | <0.001 |
| 335 | rs73428834 | 0.6099658 | 0.0617849 | <0.001 |
| 336 | rs7382061 | 0.6227715 | 0.0620495 | <0.001 |
| 337 | rs73963711 | 0.6113379 | 0.0618027 | <0.001 |
| 338 | rs7423615 | 0.6114529 | 0.0617657 | <0.001 |
| 339 | rs74299961 | 0.6108959 | 0.0617536 | <0.001 |
| 340 | rs743002 | 0.6104165 | 0.0618187 | <0.001 |
| 341 | rs74331768 | 0.6114326 | 0.0617297 | <0.001 |
| 342 | rs7441808 | 0.6122655 | 0.0617031 | <0.001 |
| 343 | rs74480102 | 0.6114688 | 0.0618224 | <0.001 |
| 344 | rs74612091 | 0.6173643 | 0.0617255 | <0.001 |
| 345 | rs746550 | 0.6106165 | 0.0617750 | <0.001 |
| 346 | rs7569084 | 0.6119884 | 0.0617683 | <0.001 |
| 347 | rs7636495 | 0.6094616 | 0.0617756 | <0.001 |
| 348 | rs7646283 | 0.6070911 | 0.0617478 | <0.001 |
| 349 | rs7646695 | 0.6119877 | 0.0617306 | <0.001 |
| 350 | rs76474320 | 0.6130292 | 0.0616492 | <0.001 |
| 351 | rs7649812 | 0.6099112 | 0.0617560 | <0.001 |
| 352 | rs76639817 | 0.6040251 | 0.0616222 | <0.001 |
| 353 | rs76793172 | 0.6139261 | 0.0618367 | <0.001 |
| 354 | rs76830965 | 0.6126234 | 0.0616785 | <0.001 |
| 355 | rs7687708 | 0.6128140 | 0.0616394 | <0.001 |
| 356 | rs76908370 | 0.6095211 | 0.0617394 | <0.001 |
| 357 | rs76981581 | 0.6084431 | 0.0616980 | <0.001 |
| 358 | rs7700687 | 0.6068528 | 0.0619359 | <0.001 |
| 359 | rs77625297 | 0.6083734 | 0.0616231 | <0.001 |
| 360 | rs778756 | 0.6122483 | 0.0617412 | <0.001 |
| 361 | rs7797428 | 0.6079565 | 0.0615679 | <0.001 |
| 362 | rs783646 | 0.6056684 | 0.0614543 | <0.001 |
| 363 | rs7839946 | 0.6102150 | 0.0617483 | <0.001 |
| 364 | rs7840212 | 0.6128969 | 0.0619412 | <0.001 |
| 365 | rs7846314 | 0.6128790 | 0.0618193 | <0.001 |
| 366 | rs7868130 | 0.6013583 | 0.0615394 | <0.001 |
| 367 | rs78691875 | 0.6128569 | 0.0614006 | <0.001 |
| 368 | rs7897422 | 0.6085714 | 0.0617471 | <0.001 |
| 369 | rs79716587 | 0.6110329 | 0.0617619 | <0.001 |
| 370 | rs7975 | 0.6102943 | 0.0617523 | <0.001 |
| 371 | rs7986796 | 0.6123397 | 0.0618071 | <0.001 |
| 372 | rs8 | 0.6097867 | 0.0617801 | <0.001 |
| 373 | rs80054178 | 0.6145036 | 0.0615297 | <0.001 |
| 374 | rs80066203 | 0.6102986 | 0.0617411 | <0.001 |
| 375 | rs8012643 | 0.6098617 | 0.0617477 | <0.001 |
| 376 | rs8020739 | 0.6067296 | 0.0617093 | <0.001 |
| 377 | rs8026803 | 0.6075784 | 0.0617933 | <0.001 |
| 378 | rs8044920 | 0.6143844 | 0.0613848 | <0.001 |
| 379 | rs8050508 | 0.6144814 | 0.0616714 | <0.001 |
| 380 | rs8061729 | 0.6090684 | 0.0616761 | <0.001 |
| 381 | rs8076052 | 0.6129584 | 0.0616919 | <0.001 |
| 382 | rs8108623 | 0.6110033 | 0.0617797 | <0.001 |
| 383 | rs8142080 | 0.6119566 | 0.0617376 | <0.001 |
| 384 | rs884634 | 0.6081526 | 0.0615929 | <0.001 |
| 385 | rs911603 | 0.6155053 | 0.0616917 | <0.001 |
| 386 | rs925966 | 0.6128317 | 0.0617257 | <0.001 |
| 387 | rs9349322 | 0.6152792 | 0.0615401 | <0.001 |
| 388 | rs9389268 | 0.6247374 | 0.0616447 | <0.001 |
| 389 | rs9392525 | 0.6086025 | 0.0616644 | <0.001 |
| 390 | rs941616 | 0.6079052 | 0.0615344 | <0.001 |
| 391 | rs954954 | 0.6110987 | 0.0618199 | <0.001 |
| 392 | rs962993 | 0.5984072 | 0.0615414 | <0.001 |
| 393 | rs964184 | 0.6130503 | 0.0617514 | <0.001 |
| 394 | rs9666598 | 0.6098716 | 0.0617588 | <0.001 |
| 395 | rs9675999 | 0.6097383 | 0.0617597 | <0.001 |
| 396 | rs9815874 | 0.6050121 | 0.0616034 | <0.001 |
| 397 | rs9818987 | 0.6125785 | 0.0616919 | <0.001 |
| 398 | rs9835307 | 0.6074515 | 0.0617684 | <0.001 |
| 399 | rs9837045 | 0.6123905 | 0.0616729 | <0.001 |
| 400 | rs9840310 | 0.6095334 | 0.0617125 | <0.001 |
| 401 | rs9872485 | 0.6109170 | 0.0617724 | <0.001 |
| 402 | rs9880192 | 0.6234598 | 0.0616095 | <0.001 |
| 403 | rs9894839 | 0.6130543 | 0.0616129 | <0.001 |
| 404 | rs9939774 | 0.6188046 | 0.0615598 | <0.001 |
| 405 | rs9979383 | 0.6066233 | 0.0617681 | <0.001 |
| 406 | All | 0.6107223 | 0.0616575 | <0.001 |

SNPs: single-nucleotide polymorphisms; se: standard error; EOS: eosinophil
